# Hypothermia for moderate or severe neonatal encephalopathy in low-income and middle-income countries (HELIX): a randomised controlled trial in India, Sri Lanka, and Bangladesh

**DOI:** 10.1016/S2214-109X(21)00264-3

**Published:** 2021-08-03

**Authors:** Sudhin Thayyil, Stuti Pant, Paolo Montaldo, Deepika Shukla, Vania Oliveira, Phoebe Ivain, Paul Bassett, Ravi Swamy, Josephine Mendoza, Maria Moreno-Morales, Peter J Lally, Naveen Benakappa, Prathik Bandiya, Indramma Shivarudhrappa, Jagadish Somanna, Usha B Kantharajanna, Ankur Rajvanshi, Sowmya Krishnappa, Poovathumkal K Joby, Kumutha Jayaraman, Rema Chandramohan, Chinnathambi N Kamalarathnam, Monica Sebastian, Indumathi A Tamilselvam, Usha D Rajendran, Radhakrishnan Soundrarajan, Vignesh Kumar, Harish Sudarsanan, Padmesh Vadakepat, Kavitha Gopalan, Mangalabharathi Sundaram, Arasar Seeralar, Prakash Vinayagam, Mohamed Sajjid, Mythili Baburaj, Kanchana D Murugan, Babu P Sathyanathan, Elumalai S Kumaran, Jayashree Mondkar, Swati Manerkar, Anagha R Joshi, Kapil Dewang, Swapnil M Bhisikar, Pavan Kalamdani, Vrushali Bichkar, Saikat Patra, Kapil Jiwnani, Mohammod Shahidullah, Sadeka C Moni, Ismat Jahan, Mohammad A Mannan, Sanjoy K Dey, Mst N Nahar, Mohammad N Islam, Kamrul H Shabuj, Ranmali Rodrigo, Samanmali Sumanasena, Thilini Abayabandara-Herath, Gayani K Chathurangika, Jithangi Wanigasinghe, Radhika Sujatha, Sobhakumar Saraswathy, Aswathy Rahul, Saritha J Radha, Manoj K Sarojam, Vaisakh Krishnan, Mohandas K Nair, Sahana Devadas, Savitha Chandriah, Harini Venkateswaran, Constance Burgod, Manigandan Chandrasekaran, Gaurav Atreja, Pallavi Muraleedharan, Jethro A Herberg, W K Kling Chong, Neil J Sebire, Ronit Pressler, Siddarth Ramji, Seetha Shankaran

**Affiliations:** a Centre for Perinatal Neuroscience, Imperial College London, London, UK; b Section of Paediatric Infectious Disease, Imperial College London, London, UK; c Statsconsultancy, Amersham, London, UK; d Perinatal Pathology, National Institute for Health Research Biomedical Research Centre, Great Ormond Street Hospital for Children, University College London, London, UK; e Department of Neurophysiology, Great Ormond Street Hospital, London, UK; f Department of Neuroradiology, Great Ormond Street Hospital, London, UK; g Perinatal Epidemiology Unit, Bengaluru, Karnataka, India; h Indira Gandhi Institute of Child Health, Bengaluru, Karnataka, India; i Institute of Child Health, Madras Medical College, Chennai, India; j Institute of Obstetrics and Gynaecology and Government Hospital for Women and Children, Madras Medical College, Chennai, India; k Barnard Institute of Radiology, Madras Medical College, Chennai, India; l Lokmanya Tilak Municipal Medical College, Mumbai, Maharashtra, India; m Sree Avittom Thirunal Hospital and Government Medical College, Thiruvananthapuram, Kerala, India; n Institute of Maternal and Child Health, Government Medical College, Kozhikode, Kerala, India; o Vanivilas Hospital, Bangalore Medical College and Research Institute, Karnataka, India; p Maulana Azad Medical College, New Delhi, India; q Bangabandhu Sheikh Mujib Medical University, Dhaka, Bangladesh; r National Institute of Neurosciences, Dhaka, Bangladesh; s University of Kelaniya, Colombo, Sri Lanka; t University of Colombo, Colombo, Sri Lanka; u Neonatal-Perinatal Medicine, Wayne State University, Detroit, MI, USA

## Abstract

**Background:**

Although therapeutic hypothermia reduces death or disability after neonatal encephalopathy in high-income countries, its safety and efficacy in low-income and middle-income countries is unclear. We aimed to examine whether therapeutic hypothermia alongside optimal supportive intensive care reduces death or moderate or severe disability after neonatal encephalopathy in south Asia.

**Methods:**

We did a multicountry open-label, randomised controlled trial in seven tertiary neonatal intensive care units in India, Sri Lanka, and Bangladesh. We enrolled infants born at or after 36 weeks of gestation with moderate or severe neonatal encephalopathy and a need for continued resuscitation at 5 min of age or an Apgar score of less than 6 at 5 min of age (for babies born in a hospital), or both, or an absence of crying by 5 min of age (for babies born at home). Using a web-based randomisation system, we allocated infants into a group receiving whole body hypothermia (33·5°C) for 72 h using a servo-controlled cooling device, or to usual care (control group), within 6 h of birth. All recruiting sites had facilities for invasive ventilation, cardiovascular support, and access to 3 Tesla MRI scanners and spectroscopy. Masking of the intervention was not possible, but those involved in the magnetic resonance biomarker analysis and neurodevelopmental outcome assessments were masked to the allocation. The primary outcome was a combined endpoint of death or moderate or severe disability at 18–22 months, assessed by the Bayley Scales of Infant and Toddler Development (third edition) and a detailed neurological examination. Analysis was by intention to treat. This trial is registered with ClinicalTrials.gov, NCT02387385.

**Findings:**

We screened 2296 infants between Aug 15, 2015, and Feb 15, 2019, of whom 576 infants were eligible for inclusion. After exclusions, we recruited 408 eligible infants and we assigned 202 to the hypothermia group and 206 to the control group. Primary outcome data were available for 195 (97%) of the 202 infants in the hypothermia group and 199 (97%) of the 206 control group infants. 98 (50%) infants in the hypothermia group and 94 (47%) infants in the control group died or had a moderate or severe disability (risk ratio 1·06; 95% CI 0·87–1·30; p=0·55). 84 infants (42%) in the hypothermia group and 63 (31%; p=0·022) infants in the control group died, of whom 72 (36%) and 49 (24%; p=0·0087) died during neonatal hospitalisation. Five serious adverse events were reported: three in the hypothermia group (one hospital readmission relating to pneumonia, one septic arthritis, and one suspected venous thrombosis), and two in the control group (one related to desaturations during MRI and other because of endotracheal tube displacement during transport for MRI). No adverse events were considered causally related to the study intervention.

**Interpretation:**

Therapeutic hypothermia did not reduce the combined outcome of death or disability at 18 months after neonatal encephalopathy in low-income and middle-income countries, but significantly increased death alone. Therapeutic hypothermia should not be offered as treatment for neonatal encephalopathy in low-income and middle-income countries, even when tertiary neonatal intensive care facilities are available.

**Funding:**

National Institute for Health Research, Garfield Weston Foundation, and Bill & Melinda Gates Foundation.

**Translations:**

For the Hindi, Malayalam, Telugu, Kannada, Singhalese, Tamil, Marathi and Bangla translations of the abstract see Supplementary Materials section.

## Introduction

Neonatal encephalopathy is the cause of 1 million deaths worldwide every year, of which 99% occur in low-income and middle-income countries (LMICs).^1^ The pooled data from clinical trials in high-income countries suggest that death or disability is reduced at 18 months through use of induced hypothermia for moderate or severe neonatal encephalopathy.^2^, ^3^, ^4^, ^5^

Although several trials have suggested that there are beneficial effects of hypothermia in LMICs, these studies have been small or poorly designed, or both, and systematic reviews have reported conflicting results.^6^, ^7^ The International Liaison Committee Resuscitation guidelines in 2015 recommended therapeutic hypothermia as the standard of care in LMICs for neonatal encephalopathy, but acknowledged that the existing evidence was weak.^8^ Nevertheless, hypothermia therapy has been extensively implemented in most LMICs as the standard care and adopted into their national guidelines.^9^

We examined whether therapeutic hypothermia that is initiated within 6 h of birth and continued for 72 h reduces death or disability at 18–22 months when compared with usual care, in full-term babies with moderate or severe neonatal encephalopathy who have been admitted to tertiary neonatal units in LMICs.

## Methods

### Study design and participants

The hypothermia for encephalopathy in low-income and middle-income countries (HELIX) trial was an open-label, multicountry, randomised controlled trial that recruited newborn infants from seven large public sector tertiary neonatal intensive care units in India (five sites: Lokmanya Tilak Municipal General Hospital and Medical College, Mumbai, Maharashtra; Indira Gandhi Institute of Child Health, Bangalore, Karnataka; Institute of Child Health, Madras Medical College, Chennai, Tamil Nadu; Institute of Obstetrics and Gynaecology, Madras Medical College, Chennai, Tamil Nadu; and Trivandrum Medical College, Kerala), Sri Lanka (Kelaniya University, Colombo), and Bangladesh (Bangabandhu Sheikh Mujib Medical University Hospital, Dhaka).

**Research in context**

**Evidence before this study**

One Cochrane review and three other systematic reviews have reported that therapeutic hypothermia reduces death or disability after neonatal encephalopathy, but these meta-analyses were primarily focused on clinical trials from high-income countries. Given the massive encephalopathy burden in low-income and middle-income countries (LMICs), the International Liaison Committee on Resuscitation guidelines (2015) recommended that therapeutic hypothermia using low-cost cooling devices could be offered in these settings, but acknowledged that the underlying evidence was weak and called for adequately powered randomised controlled trials to be done. Before the development of the hypothermia for neonatal encephalopathy in low-income and middle-income countries (HELIX) trial protocol, we did a systematic review to identify all randomised controlled trials of therapeutic hypothermia in LMICs. We searched MEDLINE, Embase, and Cochrane Central Register of Controlled Trials (from Jan 1, 1995, to Nov 30, 2012) with the keywords “hypoxic ischemic encephalopathy”, “therapeutic hypothermia”, and “LMIC”. We excluded trials done in high-income countries. After the completion of the HELIX trial, we updated the search in Dec 1, 2020. No language restrictions were applied. We identified 15 randomised controlled trials, of which nine were from India, three from China, and one each from Uganda, Turkey, and Egypt. Five trials used selective head cooling devices, and the other ten administered whole-body cooling using low-cost methods such as frozen gel packs (five trials), phase change material (four trials), and water bottles (one trial). Although the pooled data from these studies, involving a total of 1249 infants, found a significant reduction in death during neonatal hospitalisation with cooling therapy (risk ratio 0·64, 95% CI 0·51–0·81), the individual trials were small pilot studies, and of poor quality. Few data were available for long-term neurodevelopmental outcomes.

**Added value of this study**

To our knowledge, HELIX is the largest neonatal cooling trial undertaken to date and the only rigorously done multicountry randomised controlled clinical trial that is adequately powered to examine the safety and efficacy of therapeutic hypothermia in LMICs. Therapeutic hypothermia did not reduce the combined outcome of death or disability at 18 months, but significantly increased mortality compared with a control group who received supportive intensive care alone. Among survivors, no difference was seen in severe disability, microcephaly, or survival without disability between the hypothermia and control groups. We found no difference in brain injury between the groups using conventional MRI scores, proton MRI spectroscopy peak area metabolite ratios of lactate:N-acetylaspartate (NAA), NAA:choline, and NAA:creatine; absolute concentrations of NAA; or whole brain white matter fractional anisotropy. However, most infants had a white-matter injury suggesting intermittent hypoxic injury. Most infants had clinical seizures within a few hours of birth, indicating a much earlier onset of fetal brain injury than in high-income countries. These data provide conclusive evidence for an absence of neuroprotection and harm from therapeutic hypothermia for neonatal encephalopathy in LMICs, even when optimal tertiary neonatal care, ventilatory support, and servo-controlled cooling devices were available.

**Implications of all the available evidence**

The absence of hypothermic neuroprotection might be related to the subacute nature of brain injury in LMICs. Although the HELIX trial was done in well resourced south Asian tertiary neonatal units, the trial results are likely to be widely applicable to all LMICs, because a therapy that is unsafe and ineffective in a well resourced setting is unlikely to be beneficial in sub-Saharan Africa. Future research should focus on understanding the origins and timing of brain injury in these settings and in preventing neonatal encephalopathy.

All participating neonatal units had facilities for assisted ventilation, cardiovascular monitoring, and support; access to 3 Tesla MRI scanners; and were managed by a two-tier system of trainee doctors and neonatal consultants who were experienced in dealing with therapeutic hypothermia. At each site, one neonatal intensive care nurse cared for two to four babies at a time. In addition, we appointed neonatal research nurses and research fellows at each recruiting site for the trial. Only one centre had a facility for inhaled nitric oxide. Two participating centres admitted only infants who were born at locations outside the participating hospital (including other health-care facilities or those born at home), one admitted only babies who were born within the participating hospital, and the other four admitted both.

All infants born at 36 weeks of gestation or later with a birthweight of 1·8 kg or more who were admitted to the neonatal unit within 6 h of birth were screened for eligibility and recruited to the trial if they met both of the following two criteria of eligibility: first, a need for continued resuscitation at 5 min of age or an Apgar score of less than 6 at 5 min of age (for babies born in a hospital), or both, or an absence of crying by 5 min of age (for babies born at home); and second, evidence of moderate or severe encephalopathy at any time between 1 h and 6 h of age based on a structured clinical examination using modified Sarnat staging done by a certified examiner after admission to the neonatal unit.^10^ The stage of encephalopathy was established based on the predominant degree of neurological abnormality (none, mild, moderate, or severe) across the six categories.^10^ We excluded infants who had no heart rate at 10 min of age despite adequate resuscitation, those with major life-threatening congenital malformations, or if the parents were unable to attend follow-up assessments.

The research ethics committees at Imperial College London and the participating sites approved the trial.^11^ The trial was done in accordance with the principles of the Declaration of Helsinki. Informed verbal and written parental consent in local languages was obtained from parents before recruitment and the audio-visual records of the consenting process were regularly audited for quality assurance.^12^

### Randomisation and masking

We randomly assigned infants either to the group given hypothermia with supportive intensive care or the group given usual intensive care alone (control group) with a ratio of 1:1 using a web-based randomisation system (Sealed Envelope). The randomisation sequence was computer generated. The minimisation method was used to control for encephalopathy stage and study centre, with a probability of 0·85 of being assigned to the treatment group that minimised an imbalance in the number of infants assigned to each group within each prognostic factor. One of the clinical team members approached the parents for trial participation. Once parental consent was obtained, a study personnel at the site entered the basic clinical details, including date and time of birth and the stage of encephalopathy, into the randomisation system (Sealed Envelope) using a secure login. Each randomisation generated an automated e-mail to the team at Imperial College London, who then cross-checked the date and time of birth of the infant and adherence with the assigned group.

Infants who were randomly assigned to the control group had usual neonatal intensive care including invasive ventilation and inotropic support, avoidance of iatrogenic hyperthermia by restricting the use of overhead radiant warmers during resuscitation where possible, setting lower (36·0°C) thresholds in servo-controlled warmers, close monitoring of clinical and laboratory variables, and prompt correction of any metabolic or coagulation derangement.

Babies who had been allocated to the hypothermia group had a controlled reduction of their rectal temperature to 33·5°C for 72 h, followed by automated re-warming at 0·5°C per h with the use of a servo-controlled whole-body cooling device (Tecotherm Neo; Inspiration Health Care, Crawley, UK) in addition to the supportive intensive care. However, passive cooling before neonatal unit admission was not permitted.

Masking of the intervention was not possible, but those involved in the magnetic resonance (MR) biomarker analysis and neurodevelopmental outcome assessments were masked to the allocation.

### Procedures

We measured rectal temperatures in all babies every hour until 96 h of age, followed by measurements every 8 h during the first week. The clinical protocols for supportive intensive care, including inotropes, sedation, blood products, and anticonvulsants, were standardised to reduce variability across sites. Haemoglobin, leukocyte counts, C-reactive protein, glucose concentrations, coagulation profile, and blood gas measurements were obtained once per day for the first 4 days after birth. Blood cultures (measured using BD Bactec, NJ, USA) were obtained at the time of admission and repeated after 72 h in all infants. A single perinatal pathologist who was masked to the treatment allocations examined all umbilical cords for funisitis, defined by the presence of polymorphonuclear leukocytes.^13^ Non-evidence-based therapies, including steroids or mannitol, were not permitted. We maintained a low threshold for antibiotic therapy, and for blood product replacement for coagulopathies. A certified examiner (NB, PBan, JS, UBK, ARaj, SK, CNK, IAT, UDR, RSo, VKu, HS, PVa, KG, MSu, AS, PVi, MSa, KDM, JMo, SM, KD, SMB, PK, VB, SPat, MSh, SCM, IJ, MAM, SKD, RR, RSu, SSa, and ARah) did daily encephalopathy staging until day 4 after birth, and again on day 7 after birth and at discharge.

Infants undergoing hypothermia were sedated if they were distressed (shivering or unexplained tachycardia, or both). Hypothermia was discontinued if the neonate showed refractory hypotension despite optimal inotropic and volume support, or a life-threatening or massive haemorrhage, or if there was a parental or clinician request to stop cooling.

We did cerebral MRI 1–2 weeks after birth on a 3 Tesla scanner, which was a Philips scanner (Amsterdam, Netherlands) at one site (Lokmanya Tilak Municipal General Hospital and Medical College, Mumbai, Maharashtra, India), a Siemens scanner (Munich, Germany) at five sites, or a GE Healthcare scanner (Chicago, IL, USA) at one site (Trivandrum Medical College, Kerala, India), with the same protocol. The MR protocol (acquisition time) comprised T_1_-weighted and T_2_-weighted MRI (15 min) and diffusion tensor MRI (DTI; 7 min), proton MR spectroscopy metabolite peak area ratios (7 min), and metabolite absolute concentrations (13 min).^14^ We acquired magnetic resonance spectroscopy in a single 15 × 15 × 15 mm^3^ voxel centred on the left thalamus (appendix 9 pp 11–17).

Pseudonymised raw MR data were centrally post-processed after a predefined quality check to exclude poor-quality data before the data analysis. MR spectroscopy data were analysed using LCModel (version 6.3), and DTI data were analysed using Functional MRI of the Brain Software Library (version 6.0). Voxel-wise statistical analysis of the fractional anisotropy data was done using tract-based spatial statistics to evaluate the differences in whole brain white matter diffusivity between the hypothermic and control group.^14^

All MR images were reported jointly by two neonatal neurologists (ST and PMo) with more than 10 years of experience with MR imaging using a previously validated scoring system,^14^, ^15^ masked to all clinical information, with additional input from a paediatric neuroradiologist (WKKC).

Dedicated research nurses (IS, MSe, MB, and SJR) maintained regular contact with the families after discharge. A neurodevelopmental paediatrician (SM, RC, IAT, RSw, SSu, SCM, and IJ), masked to the allocation, assessed each infant at 18–22 months using the Bayley Scales of Infant Development (third edition) and did a detailed neurological examination. We administered the Bayley Scales in the appropriate local languages (Hindi, Marathi, Kannada, Tamil, Malayalam, Singhalese, Telugu, or Bangla).

We used search teams to trace families who were lost to follow-up, with input from local health workers, the British Red Cross Society, village heads, mobile phone network-based location tracing, and local police. The assessment team travelled long distances, using air travel and overnight stays, to assess families who had relocated to other parts of the country and hence were unable to attend hospital appointments. During the COVID-19 pandemic, we did the neurodevelopmental assessments in the open air or in ventilated rooms using appropriate personal protective equipment.

Finally, for infants who could not be assessed in person by any of the aforementioned means, we contacted the parents over telephone and collected clinical information using the Ages & Stages Questionnaires (third edition). Because disability cannot be sufficiently characterised using these questionaries, we used these data only for the assessment of death.

### Outcomes

The primary outcome was death or a moderate or severe disability. We defined severe disability as any one of the following: a cognitive composite score of less than 70 based on the Bayley Scales of Infant and Toddler Development third edition (Bayley-III), a gross motor function classification system level 3–5,^16^ a profound hearing impairment requiring hearing aids or a cochlear implant, or blindness. We defined moderate disability as a cognitive composite score (Bayley-III) of 70–84 and one or more of the following: a gross motor function classification system level 2, a hearing impairment with no amplification, or a persistent seizure disorder, assessed between 18–22 months.

Short-term secondary outcomes were death before hospital discharge, a major intracranial haemorrhage on cranial ultrasonography, gastric bleeds, persistent hypotension, a pulmonary haemorrhage, persistent pulmonary hypertension, prolonged blood coagulation requiring blood products (abnormal increase in coagulation tests as per the local laboratory standards), culture-proven early-onset sepsis, necrotising enterocolitis, cardiac arrhythmia, severe thrombocytopenia, persistent metabolic acidosis, pneumonia, renal failure, subcutaneous fat necrosis, an abnormal neurological examination at discharge, and duration of the hospital stay, all assessed at the time of hospital discharge. Longer-term secondary outcomes were death from any cause at 18 months, severe disability among those who survived, and microcephaly, assessed at 18–22 months.

Exploratory MRI biomarker outcomes included brain injury scores on a conventional MRI; thalamic N-acetyl aspartate (NAA) concentrations; lactate:NAA, NAA:creatine, and NAA:choline peak area ratios; and whole brain white matter fractional anisotropy. Exploratory neurodevelopmental and growth outcomes included moderate disability; survival without disability; Bayley-III cognitive, motor, and language composite scores; persistent seizure disorder; disabling cerebral palsy; gross motor function classification system level; blindness; hearing impairment; severe microcephaly; wasting; and stunting. The local site investigators also reported all other serious adverse outcomes that were not part of the predefined trial outcomes to the HELIX trial manager and to the Independent Data Monitoring Committee within 24 h of the occurrence using a standard proforma.

### Statistical analysis

We calculated the sample size required to detect a 30% relative risk reduction in the primary outcome from 50% in the control group to 35% in the intervention group with an 80% power and two-sided 5% significance level. Allowing for a 10% loss to follow-up, the required sample size was 204 infants per group.

We did the primary analysis on an intention-to-treat basis. We analysed categorical outcomes using the χ^2^ test or Fisher's exact test for rarer outcomes. The differences in outcomes between the study groups were quantified as risk ratios (RRs) with corresponding 95% CIs. We used the Mann-Whitney test to analyse continuous outcomes with a skewed distribution and the log-rank test to compare survival times between the groups. Ordinal logistic regression analysis was used to compare brain injury between the hypothermia and control groups. Stata version 15.1 was used for data analysis.

An Independent Data Monitoring Committee masked to the treatment allocation monitored the progress of the trial every 6 months during the study. The trial is registered with ClinicalTrials.gov, NCT02387385.

### Role of funding source

The funders of the study had no role in study design, data collection, data analysis, data interpretation, or writing of the report.

## Results

**Figure 1:**
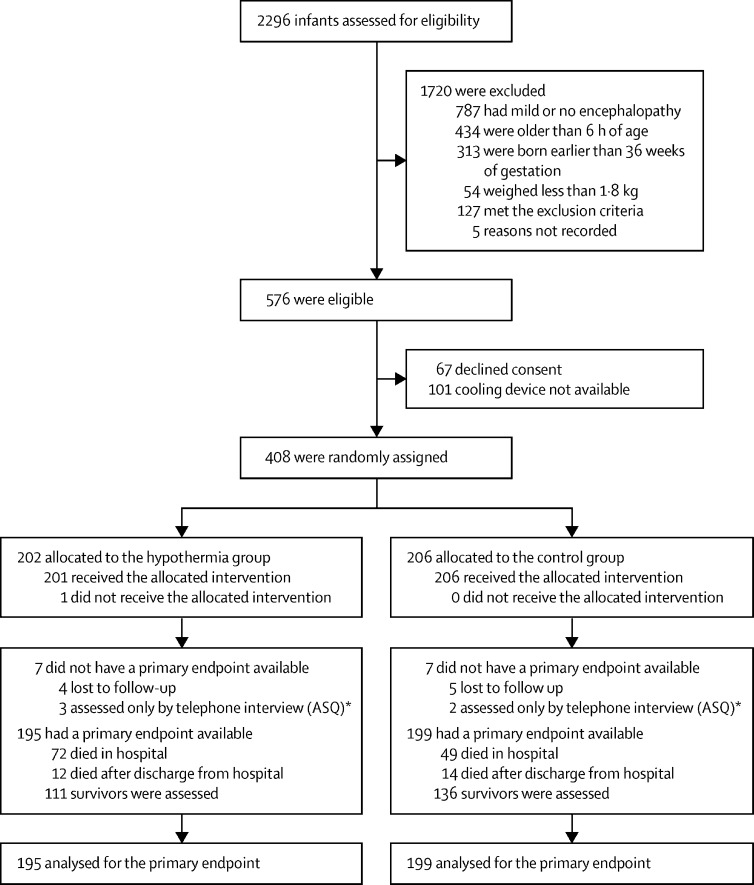
Trial profile ASQ=Ages and Stages Questionnaire. *We used the ASQ data for the assessment of mortality only and not for the assessment of disability.

Of the 2296 infants screened for eligibility between Aug 15, 2015, and Feb 15, 2019, 576 infants were considered eligible for inclusion and 1720 infants were excluded (mostly for mild or no encephalopathy, prematurity, severe growth restriction, aged older than 6 h, imminent death, other associated malformations, or if the parents were unable to come back for follow-up; figure 1). After 168 exclusions, 408 were randomly assigned to groups at the seven hospitals in India (n=347), Bangladesh (n=33), and Sri Lanka (n=28). 202 infants were allocated to the hypothermia group and 206 infants were allocated to the control group. The neurodevelopmental paediatricians did the neurological examinations between Jan 6, 2017, and Sept 27, 2020, when the infants were aged 18–22 months.

Baseline characteristics are presented in table 1. The mean maternal age was 24·5 years (SD 4·6) in the hypothermia group, versus 24·2 years (4·6) in the control group. Infants were a mean gestation of 38·9 weeks (SD 1·3) in the hypothermia group and 39·0 (1·3) in the control group. Of the infants included, 132 (65%) were male and 70 (35%) were female in the hypothermia group, versus 135 (66%) male and 71 (34%) female infants in the control group. Ten (2%) infants, all at the Bangladesh site, were born at home, and subsequently admitted to the neonatal unit; all had moderate encephalopathy and only one infant required invasive ventilation. Additional malformations were identified in one infant in the hypothermia group (complex congenital heart disease) and three infants in the control group (two had complex congenital heart disease, one had eventration of the diaphragm) during the neonatal period. None of the infants had an alternative metabolic, genetic, or infectious cause for encephalopathy.

**Table 1:** Baseline characteristics

		**Hypothermia group (n=202)**	**Control group (n=206)**
Maternal age, year		24·5 (4·6)	24·2 (4·6)
Booked pregnancies*		196 (97%)	190 (92%)
Gravida			
	1	118 (58%)	117 (57%)
	2	55 (27%)	54 (26%)
	3	22 (11%)	22 (11%)
	4	6 (3%)	11 (5%)
	5	0	2 (0%)
	Data missing	1 (0%)	0
Parity			
	0	71 (35%)	69 (33%)
	1	102 (50%)	87 (42%)
	2	22 (11%)	39 (19%)
	3	6 (3%)	8 (4%)
	Data missing	1 (0%)	3 (1%)
Complications of pregnancy (events not mutually exclusive)			
	Pregnancy-induced hypertension	9 (4%)	4 (2%)
	Others†	4 (2%)	2 (1%)
	Maternal pyrexia	3 (1%)	5 (3%)
	Long-term rupture of membranes >24 h	1 (0%)	3 (1%)
	Meconium-stained liquor	60 (30%)	50 (24%)
	Reduced fetal movements	16 (8%)	8 (4%)
	Fetal heart rate decelerations	14 (7%)	9 (4%)
	Funisitis	37 (18%)	29 (14%)
	Missing data	33 (16%)	40 (19%)
Perinatal sentinel events (events not mutually exclusive)		17 (8%)	26 (13%)
	Cord prolapse	4 (2%)	6 (3%)
	Cord around neck	2 (1%)	6 (3%)
	Lengthened second stage	2 (1%)	9 (4%)
	Obstructed labour	2 (1%)	3 (1%)
	Shoulder dystocia	1 (0%)	0
	Antepartum haemorrhage‡	6 (3%)	2 (1%)
Mode of delivery			
	Unassisted vaginal birth	133 (66%)	150 (73%)
	Instrumental delivery	21 (10%)	19 (9%)
	Elective caesarean delivery	1 (0%)	2 (1%)
	Emergency caesarean delivery	46 (23%)	34 (17%)
	Data missing	1 (0%)	1 (0%)
Place of delivery			
	Born at the participating hospital	62 (31%)	61 (30%)
	Born at another hospital§	136 (67%)	139 (67%)
	Born at home¶	4 (2%)	6 (3%)
Sex of neonate			
	Male	132 (65%)	135 (66%)
	Female	70 (35%)	71 (34%)
Age of neonate			
	Gestation, weeks	38·9 (1·3)	39·0 (1·3)
Condition at birth			
	Cord blood pH‖	6·94 (0·25)	6·97 (0·21)
	Apgar score at 5 min**	5 (4–6)	5 (4–5)
	Apgar score at 10 min**	6 (4–7)	6 (4–7)
	Endotracheal ventilation at birth	89 (44%)	89 (43%)
	Missing data	2 (1%)	5 (2%)
Infant size			
	Birthweight, g	2844 (450)	2939 (455)
	Birthweight <2 SD from the mean	36 (18%)	27 (13%)
	Head circumference, cm††	34·1 (1·5)	34·3 (1·5)
	Head circumference <2 SD from the mean††	8 (4%)	6 (3%)
	Head circumference <3 SD from the mean††	2 (1%)	2 (1%)
	Missing data	1 (0%)	1 (0%)
Age at admission to the cooling centre, min		126 (55–217)	160 (60–238)
	Born at the participating hospital	31 (15–46)	30 (18–50)
	Referred to the participating hospital	180 (120–240)	189 (139–255)
Stage of encephalopathy at randomisation			
	Moderate encephalopathy	161 (80%)	167 (81%)
	Severe encephalopathy	41 (20%)	39 (19%)
Rectal temperature at admission, °C		35·7 (1·2)	36·4 (0·7)
	Among infants with moderate encephalopathy	35·8 (1·1)	36·4 (0·7)
	Among infants with severe encephalopathy	35·3 (1·4)	36·2 (0·9)
Clinical seizures at random assignment		149 (74%)	150 (73%)

Data are number of patients (%), mean (SD), or median (IQR).

* Antenatal follow-up visits at the recruiting hospital or any other health-care facility.

† In the hypothermia group, two infants had chronic hypertension, one had epilepsy, and one had diabetes; in the control group, one had thyroid disorder and one had diabetes.

‡ In the hypothermia group, five had placental abruption and one had placenta previa; in the control group, one had placental abruption and one had placenta previa.

§ Hospitals referring the infants to the HELIX trial recruiting sites were other tertiary medical college hospitals (65 infants), secondary district hospitals (124 infants), primary care centres (44 infants), private hospitals (40 infants), and unknown (two infants).

¶ All home deliveries occurred at the site in Dhaka, Bangladesh.

‖ Cord pH was available in only 22 infants in the hypothermic group and 24 infants in the control group.

** Apgar score at 5 min was available in only 137 infants in hypothermia group and 135 infants in the control group. Apgar score at 10 min was available in only 65 infants in the hypothermia group and 67 infants in the control group.

†† WHO child growth charts 2009.^17^

In infants allocated to cooling, we started therapeutic hypothermia at a median age of 4·30 h (IQR 1·09–6·00). Between 1 h and 72 h after randomisation, the mean rectal temperature was 33·5°C (SD 0·10) in the hypothermic group and 36·7°C (0·06) in the control group; mean heart rate was 118 beats per min (1·4) in the hypothermic group and 138 beats per minute (1·3) in the control group. Mean blood pressure between 1 h and 72 h was 50·6 mm Hg (SD 6·4) in the hypothermic group and 51·8 mm Hg (6·5) in the control group (appendix 9 p 10).

Daily haematological and biochemical variables until day 4 are given in the appendix 9 (pp 2–5). At 24 h, more infants in the hypothermic group had hyperglycaemia and required more inotropic support than did infants in the control group, and at 48 h and 72 h more infants in the hypothermic group had metabolic acidosis and deranged coagulation as well. Although all infants who died were more acidotic than were those who survived in both groups at 24 h, metabolic acidosis persisted in infants in the hypothermic group who died (appendix 9 p 7).

Protocol deviations were reported in three cases allocated to the hypothermia group: one infant did not receive hypothermia therapy, another was over-cooled to 29·0°C for 3 h because of a machine malfunction, and a third had discontinuation of hypothermia because of suspected venous thrombosis and refractory hypotension. At least one episode of a rectal temperature of more than 39°C was recorded in three infants in the control group (all died) and between 38°C and 39°C in seven control infants (five died and two survived with a disability).

In the hypothermia group, 182 (90%) infants had clinical seizures with a median age at onset of 2·0 h (IQR 1·0–4·0). In the control group, 189 (92%) had clinical seizures with a median age at onset of 2·5 h (1·4–5·0). Median seizure duration, defined as the time between first and last seizure, was 20·3 h (1·0–24·0) in the hypothermia group and 19·0 h (3·0–24·0) in the control group (p=0·72). Anticonvulsant use was not different between the two groups. In the hypothermia group, 130 (64%) required only one anticonvulsant, 33 (16%) required two anticonvulsants, eight (4%) required three anticonvulsants, and one required four anticonvulsants. In the control group, 122 (59%) required only one anticonvulsant, 43 (21%) two anticonvulsants, 12 (6%) three anticonvulsants, and three (1%) four anticonvulsants (appendix 9 p 9).

Primary outcome data were available for 195 (97%) of the 202 infants in the hypothermia group, who were assessed at a mean age of 19·8 months (SD 3·8), and 199 (97%) of the 206 control infants, who were assessed at a mean age of 20·0 months (3·6). Death or moderate or severe disability occurred in 98 (50%) infants in the hypothermia group and 94 (47%) infants in the control group (RR 1·06; 95% CI 0·87–1·30; p=0·55; table 2).

**Table 2:** Outcomes at 18–22 months of age for infants

		**Hypothermia (n=202 or n=114 who survived)**	**Control (n=206 or n=138 who survived)**	**Risk ratio (95% CI)**	**p value**
**Primary outcome**					
Death or moderate or severe disability		98/195 (50%)	94/199 (47%)	1·06 (0·87–1·30)	0·55
**Predefined secondary outcomes**					
Death up until 18 months		84/198 (42%)	63/201 (31%)	1·35 (1·04–1·76)	0·022
Severe disability among those who survived		14/112 (13%)	28/136 (21%)	0·61 (0·34–1·11)	0·10
Microcephaly		33/110 (30%)	37/135 (27%)	1·09 (0·74–1·62)	0·66
**Exploratory outcomes among those who survived**					
Moderate disability		0/111	3/135 (2%)	..	..
Survival without neurodisability*		47/111 (42%)	47/136 (35%)	1·23 (0·89–1·68)	..
Bayley cognitive composite score					
	≥85	72/108 (67%)	87/133 (65%)	1 (ref)	..
	70–84	25/108 (23%)	22/133 (17%)	1·28 (0·77–2·11)	..
	<70	11/108 (10%)	24/133 (18%)	0·61 (0·32–1·18)	..
Bayley motor composite score					
	≥85	91/108 (84%)	100/133 (75%)	1 (ref)	..
	70–84	6/108 (6%)	7/133 (5%)	0·95 (0·33–2·72)	..
	<70	11/108 (10%)	26/133 (20%)	0·52 (0·27–1·00)	..
Bayley language composite score					
	≥85	61/108 (56%)	59/133 (44%)	1 (ref)	..
	70–84	29/108 (27%)	47/133 (35%)	0·73 (0·50–1·05)	..
	<70	18/108 (17%)	27/133 (20%)	0·73 (0·43–1·21)	..
Persistent seizure disorder		3/111 (3%)	9/133 (7%)	0·40 (0·11–1·44)	..
Disabling cerebral palsy†		12/111 (11%)	28/136 (21%)	0·53 (0·28–0·98)	..
GMFCS, median (IQR)		0 (0 to 0)	0 (0 to 1)	0	..
Blindness‡		5/111 (5%)	10/135 (7%)	0·61 (0·21–1·72)	..
Hearing impairment‡		3/112 (3%)	6/136 (4%)	0·60 (0·16–2·37)	..
Severe microcephaly§		19/110 (17%)	23/135 (17%)	1·01 (0·58–1·76)	..
Wasting¶		41/110 (37%)	48/134 (36%)	1·04 (0·75–1·45)	..
Stunting¶		61/111 (55%)	66/133 (50%)	1·11 (0·87–1·41)	..

Data are number of patients (%), unless otherwise indicated**.** Percentages are based on the number infants for whom data were available. GMFCS=gross motor function classification system. Median difference (95% CI) in GMFCS score between groups reported.

* Survival without neurodisability indicates infants with Bayley compositive cognitive, motor, and language scores of more than 84, a GMFCS score of 0, and no persistent seizure disorder or hearing or visual loss.

† Infants with cerebral palsy who had a GMFCS score of 2 or more.

‡ All infants with blindness or a hearing impairment also had a Bayley Scales of Infant and Toddler Development (third edition) cognitive composite score of less than 70 or GMFCS score of more than 3.

§ The term microcephaly indicates a head circumference of smaller than 2 SDs less than the mean for their age, and severe microcephaly indicates a head circumference of smaller than 3 SDs less than the mean for their age, based on WHO child growth charts in 2009.^17^

¶ The term wasting indicates a weight less than the fifth centile for their age and stunting indicates a height (length) less than the fifth centile for their age, based on WHO child growth charts in 2009.^17^

Of the 17 predefined secondary outcomes, eight differed significantly between groups (table 3); all were worse in the hypothermia group, including death before hospital discharge.

**Table 3:** Predefined secondary outcomes during neonatal hospitalisation

	**Hypothermia (n=202)**	**Control (n=206)**	Risk ratio*(95% CI)	**p value**
Intracranial haemorrhage	2 (1%)	4 (2%)	0·51 (0·09–2·75)	0·68
Gastric bleeding	62 (31%)	34 (17%)	1·86 (1·28–2·69)	<0·00070
Persistent hypotension	45 (22%)	25 (12%)	1·84 (1·17–2·88)	0·0066
Pulmonary haemorrhage	42 (21%)	28 (14%)	1·53 (0·99–2·37)	0·054
Persistent pulmonary hypertension	24 (12%)	16 (8%)	1·53 (0·84–2·79)	0·16
Prolonged blood coagulation	79 (39%)	52 (25%)	1·55 (1·16–2·07)	0·0027
Culture-positive early-onset sepsis†	12 (6%)	10 (5%)	1·22 (0·54–2·77)	0·63
Necrotising enterocolitis	5 (2%)	1 (0%)	5·10 (0·60–43·2)	0·12
Cardiac arrhythmia	5 (2%)	0	..	0·029
Severe thrombocytopenia	33 (16%)	15 (7%)	2·24 (1·26–4·00)	0·0045
Persistent metabolic acidosis	46 (23%)	24 (12%)	1·95 (1·24–3·08)	0·0029
Renal failure	22 (11%)	16 (8%)	1·40 (0·76–2·59)	0·28
Pneumonia	26 (13%)	25 (12%)	1·06 (0·63–1·77)	0·82
Subcutaneous fat necrosis	1 (0%)	0	..	..
Hospital stay,‡ days, median (IQR)	16·1 (12·9–23·2)	13·9 (11·0–18·8)	2·20 (0·70–3·80)¶	0·0044
Death before discharge	72 (36%)	49 (24%)	1·50 (1·10–2·04)	0·0087
Neurological examination at discharge§	50 (39%)	65 (41%)	0·93 (0·70–1·24)	0·61

Data are number of patients (%), unless otherwise indicated. There were no missing data. Intracranial haemorrhage refers to a major parenchymal or intraventricular bleed on cranial ultrasound. Gastric bleeding refers to more than 5 mL fresh blood produced from the nasogastric tube. Persistent hypotension refers to a mean blood pressure of less than 25 mm Hg, despite maximum inotropic support. Pulmonary haemorrhage refers to copious bloody secretions with clinical deterioration requiring change(s) in ventilatory management. Persistent pulmonary hypertension refers to severe hypoxaemia disproportionate to the severity of lung disease with a significant pre-ductal and post-ductal saturation difference on pulse oximetry. Long-term blood coagulation refers to abnormal coagulation times requiring the administration of blood products. Culture proven early-onset sepsis refers to the isolation and identification of a pathogenic organism from blood or cerebrospinal fluid, or both, along with clinical evidence of sepsis within 72 h of birth. Necrotising enterocolitis refers to abdominal distension, increased gastric aspirates, or blood in stools, or a combination, together with an abdominal x-ray showing bowel oedema, pneumatosis or pneumoperitoneum. Cardiac arrythmias included ventricular arrythmias and ectopic heartbeats requiring treatment. Severe thrombocytopenia refers to a platelet count of less than 25 000 per μL or less than 50 000 per μL with active bleeding. Persistent metabolic acidosis refers to a blood pH of less than 7·15 for more than 12 h with a normal partial pressure of carbon dioxide. Renal failure refers to anuria lasting longer than 48 h with elevated creatinine. Pneumonia refers to infiltrates on a chest x-ray consistent with infection or aspiration.

* Group difference expressed as the risk ratio of occurrence in hypothermia group relative to the control group.

† In the hypothermia group, seven infants had *Klebsiella pneumoniae* infections, two had *Pseudomonas* spp, one had *Enterobacter* spp, one had *Escherichia coli*, and one had non-fermenting Gram-negative bacilli isolated from blood culture; eight were born at the participating hospital and four were referred to the participating hospital. Among the infants in the control group, five had *K pneumoniae* infections, three had *E coli*, and two had non-fermenting Gram-negative bacilli isolated from blood culture; four were born at the participating hospital and six were referred to the participating hospital.

‡ Figures based 130 hypothermia babies, 156 control babies who survived to discharge (1 missing value for surviving babies in control group, 0 missing in hypothermia group).

§ Figures based on babies surviving to discharge only. Figures based 130 hypothermia babies, 157 control babies.

¶ Median difference (95% CI).

In the hypothermia group, 72 infants died before hospital discharge from the following causes: asphyxial brain injury-related complications (n=50 [69%]), persistent pulmonary hypertension (n=12 [17%]), subgaleal or intracranial bleed (n=5 [7%]), sepsis or pneumonia (n=4 [6%]), and complex congenital heart disease (n=1 [1%]). In the control group, 49 infants died before hospital discharge from the following causes: asphyxial brain injury-related complications (n=35 [71%]), persistent pulmonary hypertension (n=9 [18%]), subgaleal or intracranial bleed (n=3 [6%]), sepsis or pneumonia (n=1 [2%]), and complex congenital heart disease (n=1 [2%]). None of the deaths were related to the withdrawal of life support, although three infants in the hypothermia group and seven infants in the control group were discharged against medical advice.

**Figure 2:**
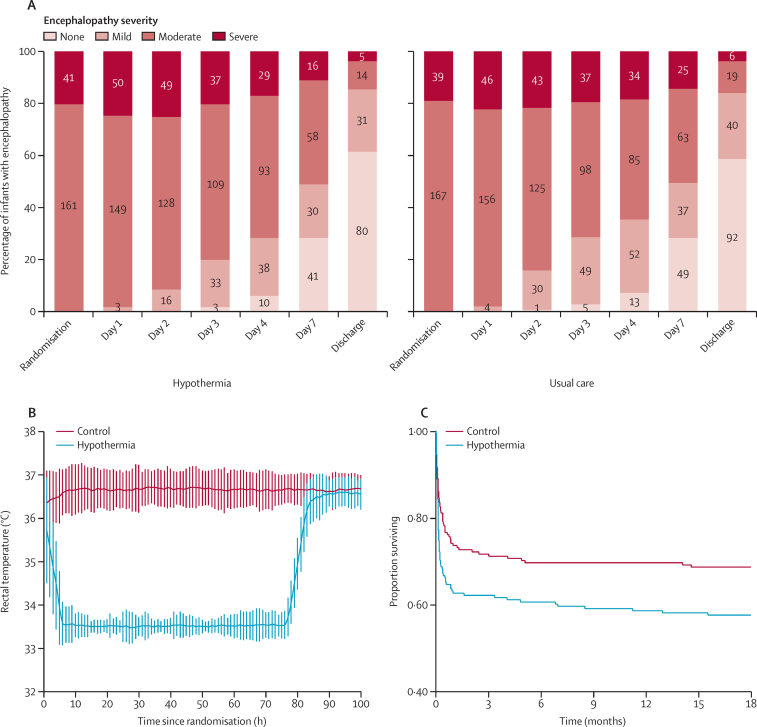
Changes in encephalopathy severity (A), temperature profile (B), and Kaplan-Meier estimates of survival until 18 months (C) Error bars indicate mean (SD) of hourly rectal temperatures in the hypothermia group and control group

After hospital discharge, a further 12 infants in the hypothermia group died by 18 months of age: three from neurological sequelae of brain injury and epilepsy (25%), three of pneumonia (25%), and six from unknown causes (50%). In the control group, 14 infants died after hospital discharge by 18 months of age: five of neurological sequelae and epilepsy (36%), three of pneumonia (21%), and six from unknown causes (43%). 84 (42%) infants in the hypothermia group and 63 (31%) in the control group died by 18 months (table 2). Survival analysis showed a significant difference between groups, with hypothermia increasing the risk of death at any time by 47·0% (hazard ratio 1·47; 95% CI 1·06–2·04; p=0·020; figure 2). One infant in the control group who had hearing loss and blindness died when they were older than 18 months. The data for this infant were not included in the mortality analysis but were included in the primary outcome analysis. The other two predefined secondary outcomes—namely, severe disability and microcephaly among those who survived—did not show a difference between the two groups (table 2). Survival without neurodisability did not significantly differ between the two groups.

Of the 408 infants included, 114 died and eight left the hospital against medical advice before the MRI scan, and two parents refused MRI (appendix 9 p 11). Thus, MRI was acquired in 284 (70%), of which data for 267 infants were available for analysis. Median age at the scan was 14 days (IQR 11–19). Among the infants who had an MRI, primary outcome data were available from 271. Death, moderate or severe disability occurred in 26 (21%) of the 123 infants in the hypothermic group and 44 (30%) of the 148 infants in the control group (RR 0·71, 95% CI 0·47–1·08; p=0·11).

26 (21%) of 122 infants in the hypothermic group and 37 (26%) of 145 in the control group had basal ganglia or thalamic injury (p=0·56), 100 (82%) in the hypothermic group and 112 (77%) in the control group had white-matter injury (p=0·44), and 31 (25%) in the hypothermic group and 46 (32%) in the control group had cortical injury (p=0·29; appendix 9 p 6). 29 (24%) of the hypothermic infants and 28 (19%) of the control group infants had small subdural bleeds (p=0·37).

**Figure 3:**
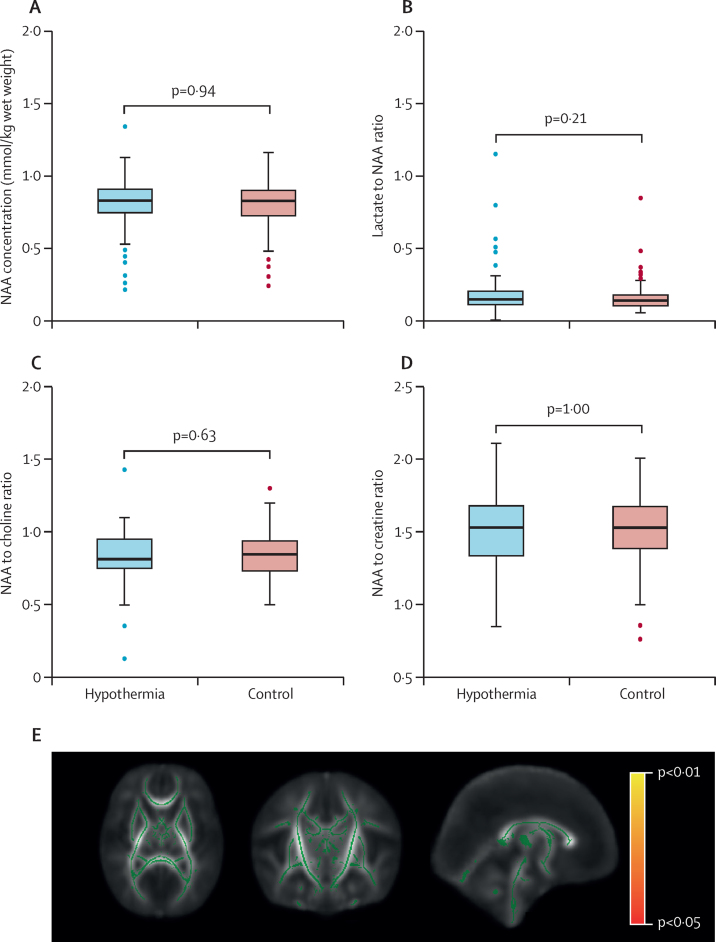
Thalamic NAA concentrations, and metabolite peak area ratios (A–D) and tract-based spatial statistics of white matter fractional anisotropy (E) (A–D) Medians are indicated by horizontal lines, boxes outline the upper and lower quartiles, and the whiskers indicate 1·5 times the IQR from the upper and lower quartiles. Outliers are indicated with dots lying beyond the whiskers. (E) Green indicates the mean fractional anisotropy tract skeleton, with a threshold range of 0·15 (lower) and 1·0 (upper) over regions with no statistically significant (p<0·05) groupwise difference in fractional anisotropy between groups. Regions in red and yellow indicate regions of increasing statistical significance (p<0·05 in red, p<0·01 in yellow), corrected for multiple comparisons over whole brain analysis. There are no areas in red or yellow indicating that the whole brain white matter fractional anisotropy in infants in the hypothermia group is not significantly different to that of the control infants. NAA=N-acetyl aspartate.

Good quality MR spectroscopy data for metabolite peak area ratio were available in 174 (81/109 [74%] hypothermia, 93/135 [69%] control) infants and for absolute quantification in 159 (72/110 [65%] hypothermia and 87/135 [64%] control) infants. The mean thalamic NAA concentration was 8·06 (SD 1·8) in infants in the hypothermic group, and 8·04 (1·6) in infants in the control group (p=0·59). The median lactate:NAA ratio was 0·14 (IQR 0·106–0·200) in the hypothermic group and 0·14 (IQR 0·099–0·175) in the control group; the mean NAA:choline ratio was 0·83 (SD 0·18) in the hypothermic group and 0·85 (SD 0·16) in the control group; and the NAA:creatine ratio was 1·51 (SD 0·29) in the hypothermic group and 1·51 (SD 0·26) in the control group (all p>0·05; figure 3).

Good quality DTI data from 140 infants (70/111 [63%] hypothermia, 70/102 [69%] control), all scanned on Siemens 3 Tesla scanners, were available for tract-based spatial statistics. Whole-brain white matter fractional anisotropy, analysed by tract-based spatial statistics, did not show any difference between the hypothermia group and control group (figure 3). The mean fractional anisotropy values over posterior limbs of the internal capsule did not significantly differ between the hypothermic (0·325 [SD 0·05]) and control (0·328 [0·06]) groups (p=0·90).

Five serious adverse events were reported: three in the hypothermia group (one hospital readmission relating to pneumonia, one septic arthritis, and one suspected venous thrombosis), and two in the control group (both related to MRI; one related to desaturations during MRI and the other because of endotracheal tube displacement during transport for MRI). No adverse events were considered causally related to the study intervention.

In the hypothermia group, the number of infants with severe encephalopathy increased from 41 (20%) at the time of random assignment to 50 (25%) at 24 h and 49 (24%) at 48 h from randomisation. In the control group, the number of infants with severe encephalopathy increased from 39 (19%) at randomisation to 46 (22%) at 24 h and 43 (21%) at 48 h (figure 2). The recovery of encephalopathy as indicated by the proportion of infants with mild or no encephalopathy was slower in cooled infants than in those in the control group (appendix 9 p 8).

Infants in the hypothermia group had more coagulopathy, gastric bleeds, cardiac arrythmia, severe thrombocytopenia, persistent metabolic acidosis, persistent hypotension, and a longer hospital stay. The need for invasive ventilation (122 [60%] *vs* 106 [52%]; RR=1·17, 95% CI 0·99–1·40; p=0·069) was similar, but inotropic support (161 [80%] *vs* 126 [61%]; RR=1·30, 95% CI 1·15–1·48; p<0·0001) was significantly higher in the hypothermia group. The rates of culture-positive late-onset sepsis (14 [7%] *vs* 11 [5%]; p=0·56), and subgaleal bleeds (seven [3%] *vs* 11 [5%]; p=0·39) were not significantly different between the hypothermia and control groups.

Culture-positive early-onset sepsis occurred in 12 (10%) of the 123 infants born in the participating hospitals and ten (4%) of the 285 infants born elsewhere and later referred to the participating hospitals. Amongst infants born in the participating hospitals, culture-positive early-onset sepsis occurred in eight (13%) of the 62 infants in the hypothermia group and four (7%) of the 61 infants in the control group (RR 1·97, 95% CI 0·62–6·20, p=0·24). Amongst infants born elsewhere and later referred to the participating hospitals, culture-positive early-onset sepsis occurred in four (3%) of the 140 infants in the hypothermia group and six (4%) of the 145 infants in the control group (RR 0·69, 95% CI 0·20–2·39, p=0·56)

## Discussion

In this multicountry, randomised controlled trial done in tertiary neonatal intensive care units in India, Sri Lanka, and Bangladesh, therapeutic hypothermia alongside optimal neonatal intensive care did not reduce the composite primary outcome of death or moderate or severe disability, but increased death alone. The HELIX trial enrolled a large number of babies from three countries using centralised random assignment, the groups were well balanced at baseline, protocol deviations were low, outcome assessors were masked to group allocations, and the loss to follow-up was minimal. The HELIX trial findings are therefore likely to be robust, and they do not support the use of hypothermia for neonatal encephalopathy in LMICs.

Apart from being the largest neonatal cooling trial reported to date, to our knowledge, HELIX is the largest prospective systematic clinical evaluation in neonatal encephalopathy using advanced 3 Tesla MRI biomarkers that has been undertaken. A rigorous approach to research governance was adhered to, including extensive training of the local teams to ensure informed parental consent without coercion, a centralised audit of audio-visual consent records, and continuous engagement with the parents and their extended families after discharge.^12^ These processes ensured that a high (97%) follow-up rate was achieved, despite frequent changes in addresses and telephone numbers.

The primary outcome measure of the HELIX trial and the event rates in the control group were similar to previous cooling trials undertaken in high-income countries.^2^, ^3^, ^4^ A composite primary outcome was chosen to address the competing effects of death and disability, because any intervention increasing the risk of death in infants who would otherwise survive with disability might falsely appear to have a neuroprotective effect. Hence, the HELIX sites with higher rates of death had lower rates of neurodisability (appendix 9 p 22).

Although the HELIX trial protocol was similar to the NICHD cooling trial,^2^ we used 5-min rather than 10-min Apgar scores, and we did not mandate umbilical cord blood acidosis as an inclusion criterion to increase the generalisability in LMICs, particularly for infants born outside of hospitals that offer cooling therapy. Despite these changes, none of the infants had mild encephalopathy, nor had any alternative metabolic or genetic causes for encephalopathy or perinatal stroke. Furthermore, the blood gas analysis at admission confirmed metabolic acidosis (appendix 9 p 2) and the MRI results were consistent with hypoxic ischaemic brain injury. We also excluded infants who had no heartbeat at 10 min of age despite adequate resuscitation, because these infants are unlikely to survive in LMICs.

The unexpected absence of neuroprotection and increased death seen in this study is in contrast with previous trials from high-income countries.^2^, ^3^, ^4^ Three factors could influence hypothermic neuroprotection: suboptimal supportive care during cooling,^18^ ineffective cooling devices,^19^ and differences in the population.^19^ The HELIX trial was carefully designed to eliminate the first two factors.

First, the HELIX trial was done in tertiary neonatal units with facilities for invasive ventilation including high-frequency oscillation, and all infants were cared for by a team of senior neonatologists, neonatal junior doctors, and nurses trained in cooling therapy. All recruiting sites were monitored closely by the Imperial College London team, who have extensive experience of therapeutic hypothermia, using real-time video conferencing to discuss clinical status daily, in addition to site visits every 3–4 months and training during the recruitment period. Although these units did not have advanced therapies such as inhaled nitric oxide and extracorporeal membrane oxygenation available, these are rarely required during hypothermic neuroprotection. Unlike the original cooling trials,^2^, ^4^ most infants undergoing hypothermia in the UK at present do not require invasive ventilation or substantial cardiovascular support.^14^

Second, hypothermia was administered using the same servo-controlled cooling device used in the UK, and the core temperatures were closely within the target range of 33·0–34·0°C. We sedated infants who were distressed, and almost all cooled infants were on sedative anticonvulsants, although the role of routine sedation during cooling is unclear.^20^, ^21^ Unlike the trials from high-income countries,^2^, ^4^ few infants in the control group of HELIX trial had hyperthermia, which might have diluted the treatment effect.^22^

Despite the steps taken to ensure optimal care and effective cooling, hypothermia was uniformly ineffective and increased the incidence of death at all trial sites, irrespective of the place of delivery (born at a participating hospital or referred from another hospital), perinatal sentinel events, growth restriction, or coexistent sepsis (appendix 9 pp 18–22). Several secondary outcomes were worse in the cooled infants; specifically, hypothermia increased persistent hypotension, length of hospital stay, metabolic acidosis, thrombocytopenia, prolonged blood coagulation, and gastric bleeding. Hypothermia also increased the need for inotropic support. The increased incidence of death in this group might be related to the hypothermia-induced suppression of hepatic metabolism in an already compromised liver, leading to persistent metabolic acidosis and myocardial depression.^23^ Although coexistent Gram-negative sepsis negates hypothermic neuroprotection in preclinical models,^24^ we did not find any relation of bloodstream-positive sepsis and hypothermic neuroprotection. Previous antibiotic therapy might have reduced culture-positive early-onset sepsis among infants referred from other hospitals (4%); however, the early-onset sepsis among infants born at the participating sites in the HELIX trial (10%) in which blood cultures were obtained before antibiotics was similar to the incidence of sepsis reported in cooling trials from high-income countries.^2^, ^3^, ^4^

To our knowledge, HELIX is the first clinical trial to systematically acquire and use 3 Tesla MRI scanners in a large group (n=284) of infants globally, including spectroscopy and DTI using validated cross-platform sequences^14^ with careful harmonisation of the MRI scanners using phantom studies and adult volunteers before the start of recruitment. The MRI biomarker data do not suggest any beneficial effect of hypothermia on brain injury. Again, these results contrast with both the NICHD trial^25^ (n=136) and the TOBY trial (n=131) data,^15^ in which hypothermia reduced brain injury on conventional MRI in a subgroup of infants who had 1·5 or 3 Tesla MRI scans as a part of clinical care.

Although none of the infants in the HELIX trial had an established brain injury, around 80% had a white-matter injury, suggesting a subacute or partial prolonged hypoxia,^26^ which might explain the absence of hypothermic neuroprotection. Basal ganglia injury, which is suggestive of an acute intrapartum hypoxic event, was seen only in 25% of the HELIX trial infants, as opposed to 79% in the TOBY trial^19^ and 48% in the NICHD trial.^15^ Furthermore, acute perinatal sentinel events were reported in less than 13% of the cases recruited to the HELIX trial, as opposed to more than 60% in the NICHD trial.^15^

Most infants in the HELIX trial had clinical seizures within a few hours of birth, suggesting that an intrapartum hypoxic injury might have occurred several hours before birth. In preclinical models, the onset of seizures indicates a secondary energy failure,^27^ and hypothermia might not be neuroprotective once seizures are well established.^28^ In the CoolCap trial, no neuroprotection was seen in infants who had seizures on amplitude-integrated electroencephalography at random assignment.^3^ In the HELIX trial, we did not use electroencephalography, and hence clinical seizures might have been over-reported.

In the HELIX trial, many infants were born outside of hospital and hence we were unable to evaluate the quality of intrapartum, resuscitation, and preadmission neonatal care, particularly when the infants were referred from small nursing homes. Although the trial was done in south Asian tertiary neonatal units with good resources, the trial results are likely to be widely applicable to all LMICs; a therapy that is unsafe and ineffective in a well resourced setting is unlikely to be beneficial in sub-Saharan Africa. Finally, we examined only umbilical cords for funisitis, and, hence, we cannot exclude chorioamnionitis and fetal inflammation. Mechanistic studies using multiomics approaches might provide further insights into absence of hypothermic neuroprotection in LMICs.^29^

Given the subacute nature of fetal brain injury, addressing the socioeconomic and nutritional risk factors of encephalopathy alongside high-quality intrapartum monitoring for early identification of a subtle fetal compromise might be important in reducing the encephalopathy burden in these settings. Preclinical models of acute hypoxic insult or dual hit infection and hypoxic insult^30^ are unlikely to capture the complexity and clinical heterogeneity of encephalopathy in LMICs and might be misleading. Future research exploring the origins and timing of birth-related brain injury might help to develop effective neuroprotective interventions. Neuro-regenerative therapies such as erythropoietin^31^ might be more effective in these settings than therapies with an acute neuroprotective effect alone.

In summary, the HELIX trial data suggest that therapeutic hypothermia alongside optimal tertiary neonatal intensive care do not reduce brain injury on MRI, nor improve the combined outcomes of death or disability after neonatal encephalopathy in LMICs, but significantly increase the incidence of death relative to a control group.

## Data sharing

The trial protocol, statistical analysis plan, and case report forms are available from the corresponding author for a period of 10 years from the publication of this manuscript. Individual de-identified participant data (including data dictionaries) will be available from the corresponding author and will be shared with individuals and institutions with appropriate expertise for additional analysis, once sub-studies from the HELIX trial are reported.

## Declaration of interests

JW reports a grant from the National Institutes for Health (number 1R21HD093563-01) outside the submitted work. RP reports personal fees from UCB and Kephala outside the submitted work. All other authors declare no competing interests.

## Supplementary Materials

Hindi translation of the abstract

Malayalam translation of the abstract

Telugu translation of the abstract

Kannada translation of the abstract

Singhalese translation of the abstract

Tamil translation of the abstract

Marathi translation of the abstract

Bangla translation of the abstract
