# Correction to *Lancet Glob Health* 2021; 9: e1273–85

**DOI:** 10.1016/S2214-109X(21)00397-1

**Published:** 2021-08-24

*Thayyil S, Pant S, Montaldo P, et al. Hypothermia for moderate or severe neonatal encephalopathy in low-income and middle-income countries (HELIX): a randomised controlled trial in India, Sri Lanka, and Bangladesh.* Lancet Glob Health *2021;* 9: *e1273–85*—The appendix of this Article has been corrected to include the list of members of the HELIX consortium group as of Aug 24, 2021.

