# Supplementary material for: Hypothermia for moderate or severe neonatal encephalopathy in low-income and middle-income countries (HELIX): a randomised controlled trial in India, Sri Lanka, and Bangladesh
Source: Lancet Glob Health. 2021 Aug 3;9(9):e1273–85. doi: 10.1016/S2214-109X(21)00264-3 (PMC8371331; doi:10.1016/S2214-109X(21)00264-3)
Supplement: Hindi translation of the abstract [file mmc1.pdf]

THE LANCET
Global Health

Supplementary appendix 1

This translation in Hindi was submitted by the authors and we reproduce it as supplied. It has not been peer reviewed. *The Lancet*'s editorial processes have only been applied to the original in English, which should serve as reference for this manuscript.

हिंदी में यह अनुवाद लेखकों द्वारा प्रस्तुत किया गया था और हम इसे जैसे उपलब्ध कराया गया वैसे पुन: पेश करते हैं। इस पर सहकर्मी की समीक्षा नहीं की गई है। लैंसेट की संपादकीय प्रक्रियाओं को केवल अंग्रेजी में मूल पर लागू किया गया है, जो इस पांडुलिपि के संदर्भ के रूप में काम आना चाहिए।

Supplement to: Thayyil S, Pant S, Montaldo P, et al. Hypothermia for moderate or severe neonatal encephalopathy in low-income and middle-income countries (HELIX): a randomised controlled trial in India, Sri Lanka, and Bangladesh. *Lancet Glob Health* 2021; published online Aug 3. http://dx.doi.org/10.1016/S2214-109X(21)00264-3.

सारांश

पृष्ठभूमि

यद्यपि उच्च-आय वाले देशों में नवजात मस्तिष्क विकृति (एन्सेफैलोपैथी) के बाद चिकित्सीय अल्पताप (हाइपोथर्मिया) मृत्यु या विकलांगता को कम करता है, लेकिन निम्न और मध्यम-आय वाले देशों में इसकी सुरक्षा और प्रभावकारिता अस्पष्ट बनी हुई है। हमने यह जांचने का लक्ष्य रखा है कि दक्षिण एशिया में नवजात एन्सेफैलोपैथी के बाद युक्ततम सहायक गहन देखभाल के साथ चिकित्सीय हाइपोथर्मिया मृत्यु या मध्यम या गंभीर विकलांगता को कम करता है या नहीं।

प्रणाली

हमने भारत, श्रीलंका और बांग्लादेश में सात तृतीयक नवजात गहन देखभाल इकाइयों में एक बहु-देशीय ओपन-लेबल, यादृच्छिक नियंत्रित परीक्षण (Randomised Controlled Trial) किया। हमने 36 सप्ताह के गर्भ में या उसके बाद मध्यम या गंभीर नवजात एन्सेफैलोपैथी के साथ और 5 मिनट की उम्र में निरंतर पुनर्जीवन की आवश्यकता या 5 मिनट की उम्र में 6 से कम का अपगार स्कोर (अस्पताल में पैदा हुए बच्चों के लिए), या दोनों, या 5 मिनट की उम्र तक रोने की अनुपस्थिति (घर पर पैदा हुए बच्चों के लिए) के साथ पैदा हुए शिशुओं को नामांकित किया। वेब-आधारित रैंडमाइजेशन सिस्टम का उपयोग करते हुए, हमने शिशुओं को जन्म के ६ घंटे के भीतर एक सर्वो-नियंत्रित शीतलन उपकरण का उपयोग करके पूरे शरीर के हाइपोथर्मिया (33·50 डिग्री सेल्सियस) प्राप्त करने वाले समूह में, या सामान्य देखभाल का उपयोग करके (नियंत्रण समूह) में आवंटित किया। सभी भर्ती केन्द्रों में आक्रामक संवातक, हृदय संबंधी सहायता और 3 टेस्ला एमआरआई स्कैनर और स्पेक्ट्रोस्कोपी तक पहुंच की सुविधा थी। हस्तक्षेप की गुप्तता संभव नहीं थी, लेकिन चुंबकीय अनुनाद बायोमार्कर विश्लेषण और न्यूरोडेवलपमेंटल परिणाम आकलन में शामिल लोगों को आवंटन से अनभिज्ञ रखा गया था। प्राथमिक परिणाम 18-22 महीनों में मृत्यु या मध्यम या गंभीर विकलांगता का एक संयुक्त समापन बिंदु था, जिसका मूल्यांकन शिशु विकास के बेली स्केल (संस्करण 3) और एक विस्तृत न्यूरोलॉजिकल परीक्षा द्वारा किया गया था। विश्लेषण के इलाज के इरादे से किया गया था। यह परीक्षण ClinicalTrials.gov, NCT02387385 के साथ पंजीकृत है।

जाँच - परिणाम

हमने 15 अगस्त, 2015 और 15 फरवरी, 2019 के बीच 2296 संभावित पात्र शिशुओं की जांच की, जिनमें से 576 शिशु शामिल होने के योग्य थे। अपवर्जन के बाद, हमने 408 योग्य शिशुओं की भर्ती की और हमने 202 को हाइपोथर्मिया समूह और 206 को नियंत्रण समूह में समुनदेशित किया। प्राथमिक परिणाम डेटा हाइपोथर्मिया समूह में 202 शिशुओं में से 195 (97%) और 206 नियंत्रण समूह शिशुओं के 199 (97%) के लिए उपलब्ध थे। हाइपोथर्मिया समूह में 98 (50%) शिशु और नियंत्रण समूह में 94 (47%) शिशुओं की मृत्यु हो गई अथवा उनमें मध्यम या गंभीर विकलांगता पायी गई (जोखिम अनुपात 1.06; 95% सीआई 0·87–1·30; पी = 0·55)। हाइपोथर्मिया समूह में 84 शिशुओं (42%) और नियंत्रण समूह में 63 (31%; पी = 0.022) शिशुओं की मृत्यु हुई, जिनमें से 72 (36%) और 49 (24%; पी = 0.0087) की मृत्यु नवजात अस्पताल में भर्ती के दौरान हुई। पांच गंभीर प्रतिकूल घटनाओं की सूचना दी गई: हाइपोथर्मिया समूह में तीन (निमोनिया से संबंधित एक अस्पताल में पठन-पाठन, एक सेप्टिक गठिया, और एक संदिग्ध शिरापरक घनास्त्रता), और दो नियंत्रण समूह में (एक एमआरआई के दौरान असंतृप्ति से संबंधित और अन्य एमआरआई के लिए परिवहन के दौरान एंडोट्रैचियल

ट्यूब विस्थापन के कारण)। अध्ययन हस्तक्षेप से संबंधित किसी भी प्रतिकूल घटना को यथोचित रूप से संबंधित नहीं माना गया।

व्याख्या

चिकित्सीय हाइपोथर्मिया ने निम्न-आय और मध्यम-आय वाले देशों में नवजात एन्सेफैलोपैथी के 18 महीने बाद मृत्यु या विकलांगता के संयुक्त परिणाम को कम नहीं किया, लेकिन अकेले मृत्यु में काफी वृद्धि हुई। चिकित्सीय हाइपोथर्मिया को निम्न-आय और मध्यम आय वाले देशों में नवजात एन्सेफैलोपैथी के उपचार के रूप में पेश नहीं किया जाना चाहिए, भले ही तृतीयक नवजात गहन देखभाल सुविधाएं उपलब्ध हों।

अनुदान

नेशनल इंस्टीट्यूट फॉर हेल्थ रिसर्च, गारफील्ड वेस्टन फाउंडेशन और बिल एंड मेलिंडा गेट्स फाउंडेशन।
