# Supplementary material for: Hypothermia for moderate or severe neonatal encephalopathy in low-income and middle-income countries (HELIX): a randomised controlled trial in India, Sri Lanka, and Bangladesh
Source: Lancet Glob Health. 2021 Aug 3;9(9):e1273–85. doi: 10.1016/S2214-109X(21)00264-3 (PMC8371331; doi:10.1016/S2214-109X(21)00264-3)
Supplement: Malayalam translation of the abstract [file mmc2.pdf]

THE LANCET
Global Health

Supplementary appendix 2

This translation in Malayalam was submitted by the authors and we reproduce it as supplied. It has not been peer reviewed. *The Lancet*'s editorial processes have only been applied to the original in English, which should serve as reference for this manuscript.

“ഇവിടെ ഭാഷ ചേർക്കുക] -ലെ ഈ വിവർത്തനം റൈറ്റേഴ്സ് സമർപ്പിച്ചതാണ്, നൽകപ്പെട്ടതുപോലെ ഞങ്ങൾ അത് പുനർനിർമ്മിക്കുന്നു. ഇത് സമഗ്രമായി അവലോകനം ചെയ്തിട്ടില്ല. ലാൻസെറ്റിന്റെ എഡിറ്റോറിയൽ പ്രോസസ്സുകൾ ഇംഗ്ലീഷിലെ ഒറിജിനലിൽ മാത്രമേ പ്രയോഗിച്ചിട്ടുള്ളൂ, അത് ഈ കൈയെഴുത്തുപ്രതിയുടെ റഫറൻസായി വർത്തിക്കും"

Supplement to: Thayyil S, Pant S, Montaldo P, et al. Hypothermia for moderate or severe neonatal encephalopathy in low-income and middle-income countries (HELIX): a randomised controlled trial in India, Sri Lanka, and Bangladesh. *Lancet Glob Health* 2021; published online Aug 3. http://dx.doi.org/10.1016/S2214-109X(21)00264-3.

സംഗ്രഹം

പശ്ചാത്തലം

ഉയർന്ന വരുമാനമുള്ള രാജ്യങ്ങളിൽ ഹൈപോതെർമിയ ചികിത്സ (therapeutic hypothermia) നവജാതശിശുക്കളുടെ തലച്ചോറിനുള്ള ക്ഷതം (നവജാത എൻസെഫലോപ്പതി) ഉണ്ടായ ശേഷം മരണമോ വൈകല്യമോ കുറയ്ക്കുന്നുണ്ടെങ്കിലും, താഴ്ന്ന-ഇടത്തരം വരുമാനമുള്ള രാജ്യങ്ങളിൽ (എൽഎംഐസി) അതിന്റെ സുരക്ഷയും കാര്യക്ഷമതയും വ്യക്തമല്ല. തീവ്ര പരിചരണത്തിന്റെ കൂടെ ഹൈപോതെർമിയ ചികിത്സ മിതമായ(moderate) മുതൽ കഠിനമായ(severe) എൻസെഫാലോപ്പതിക്ക് തെക്കേ ഏഷ്യയിൽ ഫലപ്രദമാകുമോ എന്ന് ഞങ്ങൾ ഈ പഠനത്തിലൂടെ ലക്ഷ്യമിടുന്നു.

രീതികൾ

2015 ആഗസ്റ്റ് മുതൽ 2020 സെപ്റ്റംബർ വരെ ഇന്ത്യ, ശ്രീലങ്ക, ബംഗ്ലാദേശ് എന്നിവിടങ്ങളിലെ ഏഴ് തൃതീയ നവജാതശിശു തീവ്രപരിചരണ വിഭാഗങ്ങൾ ഉൾപ്പെടുന്ന ഒരു മൾട്ടി-കൺട്രി ഓപ്പൺ ലേബൽ റാൻഡമൈസ്ഡ് കൺട്രോൾ ട്രയൽ ഞങ്ങൾ നടത്തി. 36 ആഴ്ച ഗർഭകാലത്തോ അതിനുശേഷമോ ജനിച്ചതിനു ശേഷം, 5 മിനുട്ടോ അതിൽ അധികമോ കരയാതിരിക്കുകയോ, 5 മിനിറ്റ് നീളം കുട്ടിക്ക് കൃത്രിമശ്വാസം കൊടുക്കയോ അഥവാ APGAR സ്കോർ 5 മിനുറ്റിൽ 6 ഇൽ താഴെ ഉള്ള ശിശുക്കളെ ആണ് പഠനത്തിന് വിധേയമാക്കിയത്. മിതമായ അല്ലെങ്കിൽ കഠിനമായ നവജാത എൻസെഫലോപ്പതിയുള്ള ശിശുക്കൾ ജനിച്ച് ആറ് മണിക്കൂറിനുള്ളിൽ രണ്ട് ഗ്രൂപ്പുകളായി തിരിച്ചു - ഒരു സെർവോ നിയന്ത്രിത കൂളിംഗ് ഉപകരണം ഉപയോഗിച്ച് 72 മണിക്കൂർ മുഴുവൻ ശരീരം ഹൈപോതെർമിയ (33 · 5 സി ഡിഗ്രി സെൽഷ്യസ്) നടത്തുന്ന ഗ്രൂപ്പ്, അല്ലെങ്കിൽ സാധാരണ പരിചരണം നടത്തുന്ന ഗ്രൂപ്പ് (നിയന്ത്രണ ഗ്രൂപ്പ്). പങ്കെടുത്ത എല്ലാ ആശുപത്രികളിലും വെന്റിലേറ്റർ, തീവ്രപരിചരണം, 3 ടെസ്ല MRI, സ്പെക്ട്രോസ്കോപ്പി എന്നീ സംവിധാനങ്ങൾ ഉണ്ടായിരുന്നു. ഹൈപോതെർമിയ ചികിത്സയുടെ മാസ്കിങ് ചെയ്യാൻ സാധ്യമായില്ലെങ്കിലും, MRI റിപ്പോർട്ടിങ് ചെയ്യുന്നവരും, കുട്ടികളുടെ വൈകല്യങ്ങൾ പരിശോധിച്ചവരും മാസ്കിങ് നു വിധേയമായിരുന്നു. 18 മുതൽ 22 മാസം വരെ പ്രായമുള്ള ശിശുക്കളിലെ മരണമോ, ബെയ്‌ലി സ്കെയിൽ ഓഫ് ഇൻഫന്റ് ഡെവേലോപ്മെന്റ് സ്കെയിൽ (വേർഷൻ 3) പരിശോധനയുടെ അടിസ്ഥാനത്തിൽ മിതമായതോ കഠിനമായതോ ആയ വൈകല്യമോ ആയിരുന്നു പ്രാഥമിക ഫലം.

കണ്ടെത്തലുകൾ

ഓഗസ്റ്റ് 15, 2015 ഉം ഫെബ്രുവരി 15 , 2019 ഇനും ഇടയിൽ 2296 പഠനത്തിന് അർഹരായ ശിശുക്കളിൽ നിന്നും 576 ശിശുക്കളെ ഉൾപ്പെടുത്തി. അതിൽ യോഗ്യരായ 408 ശിശുക്കളിൽ, ഞങ്ങൾ 202 ശിശുക്കളെ ഹൈപ്പോതെർമിയയ്ക്കും 206 പേരെ നിയന്ത്രണ ഗ്രൂപ്പിലേക്കും നിയോഗിച്ചു. പ്രാഥമിക ഫലം ഹൈപോതെർമിയ വിഭാഗത്തിലെ 202 കുഞ്ഞുങ്ങളിൽ 195 (97 %) കുഞ്ഞുങ്ങൾക്കാണ് ലഭിച്ചത് . നിയന്ത്രണ വിഭാഗത്തിൽ ഉൾപ്പെടുത്തിയ 206 കുഞ്ഞുങ്ങളിൽ 199 (97%) കുഞ്ഞുങ്ങൾക്കും പ്രാഥമിക ഫലം ലഭിച്ചു. 98 (50%) ശിശുക്കൾ ഹൈപ്പോതെർമിയയിലും, 94 (47%) ശിശുക്കൾ കൺട്രോൾ ഗ്രൂപ്പിലും മരണത്തിനോ മിതമായതോ കഠിനമായതോ ആയ വൈകല്യത്തിനോ വിധേയരായി (റിസ്ക് റേഷ്യോ (RR) 1·06; 95% കോൺഫിഡൻസ് ഇന്റർവെൽ (സിഐ) 0·87 - 1·30 (പി = 0·55)). ഹൈപ്പോതെർമിയ ഗ്രൂപ്പിലെ 84 ശിശുക്കളും (42%) കൺട്രോൾ കെയർ ഗ്രൂപ്പിലെ 63 (31%) (പി = 0·022) ശിശുക്കളും മരിച്ചു, ഇവരിൽ 72 ഉം (36%), 49 ഉം (24%) (പി = 0·0087) ശിശുക്കൾ ആശുപത്രി വാസത്തിനിടയിൽ മരിച്ചു. 5 കഠിനമായ പ്രതികൂലഫലങ്ങൾ കാണപ്പെട്ടു; അതിൽ 3 എണ്ണം ഹൈപോതെർമിയ വിഭാഗത്തിലും (1 ന്യൂമോണിയ ബാധിച്ചു വീണ്ടും ആശുപത്രിയിൽ പ്രവേശിപ്പിച്ചത് , 1 സെപ്റ്റിക് ആർത്രൈറ്റിസ്, 1 സംശയാസ്പദമായ വീനസ് ത്രോംബോസിസ്), 2 എണ്ണം കണ്ട്രോൾ വിഭാഗത്തിലും (1 MRI ചെയ്യുന്നതിനിടെയുള്ള ഓക്സിജൻ കുറവ്, 1 MRI ചെയ്യാൻ പോവുന്നതിനിടെ ഉണ്ടായ എൻഡോട്രക്കിയൽ ട്യൂബ് പുറത്തായത്). ഒരു പ്രതികൂലഫലവും പഠനത്തിന്റെ ഭാഗമായുള്ള ഇടപെടൽ കാരണം ആയിരുന്നില്ല.

വ്യാഖ്യാനം

താഴ്ന്ന അഥവാ ഇടത്തരം വരുമാനമുള്ള രാജ്യങ്ങളിൽ നവജാത ശിശുക്കളെ ബാധിക്കുന്ന ഇൻസെഫലോപ്പതി ക്ക് ഹൈപോതെർമിയ ചികിത്സ 18 മാസത്തിൽ മരണത്തെയോ വൈകല്യത്തെയോ ഗണ്യമായി കുറക്കുന്നില്ല. മറിച്, ഹൈപോതെർമിയ മരണത്തെ വർധിപ്പിക്കുന്നു. ആയതിനാൽ, താഴ്ന്ന അഥവാ ഇടത്തരം വരുമാനമുള്ള രാജ്യങ്ങളിൽ തീവ്ര പരിചരണ സംവിധാനങ്ങൾ ഉണ്ടെങ്കിൽ പോലും ശിശുക്കളുടെ ഇൻസെഫലോപ്പതി യുടെ ചികിത്സക്ക് ഹൈപോതെർമിയ ഉപയോഗിക്കാൻ പാടുള്ളതല്ല.
