# Supplementary material for: Hypothermia for moderate or severe neonatal encephalopathy in low-income and middle-income countries (HELIX): a randomised controlled trial in India, Sri Lanka, and Bangladesh
Source: Lancet Glob Health. 2021 Aug 3;9(9):e1273–85. doi: 10.1016/S2214-109X(21)00264-3 (PMC8371331; doi:10.1016/S2214-109X(21)00264-3)
Supplement: Telugu translation of the abstract [file mmc3.pdf]

THE LANCET
Global Health

Supplementary appendix 3

This translation in Telugu was submitted by the authors and we reproduce it as supplied. It has not been peer reviewed. *The Lancet*'s editorial processes have only been applied to the original in English, which should serve as reference for this manuscript.

ఈ తెలుగులోని అనువాదం రచయితల ద్వారా సబ్మిట్ చేయబడింది మరియు ఎలా ఇవ్వబడిందో అలా మేం ఇచ్చాం. ఇది పీర్ రివ్యూ చేయబడలేదు. లాన్సెంట్ ఎడిటోరియల్ ప్రాసెస్‌లు ఇంగ్లిష్‌లో ఉన్న ఒరిజినల్‌కు మాత్రమే వర్తిస్తుంది. ఈ మ్యానుస్క్రిప్ట్ కొరకు రిఫరెన్స్ వలే పనిచేయాలి.

Supplement to: Thayyil S, Pant S, Montaldo P, et al. Hypothermia for moderate or severe neonatal encephalopathy in low-income and middle-income countries (HELIX): a randomised controlled trial in India, Sri Lanka, and Bangladesh. *Lancet Glob Health* 2021; published online Aug 3. http://dx.doi.org/10.1016/S2214-109X(21)00264-3.

సారాంశం

నేపథ్య

అల్పోష్ణస్థితి చికిత్సా (**Therapeutic hypothermia**) అధిక ఆదాయ దేశాలలో నియోనాటల్ ఎన్సెఫలోపతి తరువాత మరణం లేదా వైకల్యాన్ని తగ్గిస్తున్నప్పటికీ, తక్కువ-ఆదాయ మరియు మధ్య-ఆదాయ దేశాలలో దాని భద్రత మరియు సమర్థత అస్పష్టంగా ఉంది. దక్షిణ ఆసియాలో నియోనాటల్ ఎన్సెఫలోపతి తర్వాత, ఆప్టిమల్ సపోర్టివ్ ఇంటెన్సివ్ కేర్‌తో పాటు అల్పోష్ణస్థితి చికిత్సా మరణాన్ని తగ్గిస్తుందా లేదా మితమైన లేదా తీవ్రమైన వైకల్యాన్ని తగ్గిస్తుందా అని పరిశీలించాలని మేము లక్ష్యంగా చేసుకున్నాము.

పద్ధతులు

మేము భారతదేశం, శ్రీలంక మరియు బంగ్లాదేశ్‌లోని ఏడు తృతీయ నియోనాటల్ ఇంటెన్సివ్ కేర్ యూనిట్లలో బహుళ-దేశ ఓపెన్-లేబుల్, రాండమైజ్డ్ కంట్రోల్డ్ ట్రయల్ చేసాము. మేము 36 వారాల గర్భధారణ సమయంలో లేదా తరువాత జన్మించిన శిశువులను మితమైన లేదా తీవ్రమైన నియోనాటల్ ఎన్సెఫలోపతితో చేర్చుకున్నాము మరియు 5 నిమిషాల వయస్సులో నిరంతరాయంగా పునరుజ్జీవనం చేయాల్సిన అవసరం లేదా 5 నిమిషాల వయస్సులో 6 కంటే తక్కువ ఎపిగార్ స్కోరు (ఆసుపత్రిలో జన్మించిన శిశువులకు), లేదా రెండూ, లేదా 5 నిమిషాల వయస్సులో ఏడుపు లేకపోవడం (ఇంట్లో జన్మించిన శిశువులకు). వెబ్-ఆధారిత రాండమైజేషన్ వ్యవస్థను ఉపయోగించి, శిశువులను మొత్తం శరీర అల్పోష్ణస్థితిని (33 • 5 ° C) 72 గంటలకు సర్వో-నియంత్రిత శీతలీకరణ పరికరాన్ని ఉపయోగించి లేదా సాధారణ సంరక్షణకు (నియంత్రణ సమూహం) పుట్టిన 6 గంటలలోపు కేటాయించాము.

అన్ని నియామక సైట్లలో ఇన్వాసివ్ వెంటిలేషన్, కార్డియోవాస్కులర్ సపోర్ట్ మరియు 3 టెస్లా MRI స్కానర్లు మరియు స్పెక్ట్రోస్కోపీకి సదుపాయాలు ఉన్నాయి. జోక్యం యొక్క మాస్కింగ్ సాధ్యం కాదు, కానీ మాగ్నెటిక్ రెసొనెన్స్ బయోమార్కర్ విశ్లేషణ మరియు న్యూరో డెవలప్‌మెంటల్ ఫలితాల అంచనాలలో పాల్గొన్నవారు కేటాయింపుకు ముసుగు వేయబడ్డారు. ప్రాథమిక ఫలితం 18-22 నెలల్లో మరణం లేదా మితమైన లేదా తీవ్రమైన వైకల్యం యొక్క సంయుక్త ముగింపు స్థానం, బేలీ స్కేల్స్ ఆఫ్ ఇన్ఫాంట్ డెవలప్‌మెంట్ (వెర్షన్ 3) మరియు ఒక వివరణాత్మక నాడీ పరీక్ష ద్వారా అంచనా వేయబడింది. చికిత్స చేయాలనే ఉద్దేశ్యంతో విశ్లేషణ జరిగింది. ClinicalTrials.gov, NCT02387385 లో నమోదు చేయబడింది.

అన్వేషణలు

మేము ఆగస్టు 15, 2015 మరియు ఫిబ్రవరి 15, 2019 మధ్య 2296 మంది అర్హతగల శిశువులను పరీక్షించాము, వీరిలో 576 మంది శిశువులు చేర్చడానికి అర్హులు. మినహాయింపుల తరువాత, మేము 408 అర్హతగల శిశువులను నియమించాము మరియు మేము అల్పోష్ణస్థితి సమూహానికి 202 మరియు నియంత్రణ సమూహానికి 206 ని కేటాయించాము. అల్పోష్ణస్థితి సమూహంలోని 202 మంది శిశువులలో 195 (97%) మందికి మరియు 206 నియంత్రణ సమూహ శిశువులలో 199 (97%) మందికి ప్రాథమిక ఫలిత డేటా అందుబాటులో ఉంది. అల్పోష్ణస్థితి సమూహం లో 98 (50 %) మరియు నియంత్రణ సమూహం లో 94 (47 %) శిశువులకు మరణం లేదా మితమైన లేదా తీవ్రమైన వైకల్యం సంభవించింది. (రిస్క్ రేషియో (RR) 1 · 06; 95% (CI) 0 · 87-1·30 (p = 0 · 55).

అల్పోష్ణస్థితి సమూహంలో 84 శిశువులు (42 %) మరియు నియంత్రణ సంరక్షణ సమూహంలో 63 (31 %) (p = 0 · 022) శిశువులు మరణించారు, వీరిలో 72 (36 %) మరియు 49 (24 %) (p = 0 · 0087) ఆసుపత్రిలో మరణించారు.

ఐదు తీవ్రమైన ప్రతికూల సంఘటనలు నివేదించబడ్డాయి: అల్పోష్ణస్థితి సమూహంలోమూడు (న్యుమోనియాకు సంబంధించిన ఒక ఆసుపత్రి పునఃప్రవేశం, ఒక సెప్టిక్ ఆర్థరైటిస్, మరియు ఒక అనుమానాస్పద సిరల త్రంబోసిస్), మరియు నియంత్రణ సమూహంలో రెండు (ఒకటి ఎంఆర్ఐ సమయంలో డీశాచ్యురేషన్ లకు సంబంధించినది మరియు మరొకటి ఎంఆర్ఐ కోసం రవాణా సమయంలో ఎండోట్రాక్హైల్ ట్యూబ్ స్థానభ్రంశం కారణంగా). అధ్యయన జోక్యానికి సంబంధించిన ప్రతికూల ఘటనలు ఏవీ పరిగణించబడలేదు.

వ్యాఖ్యానం

తక్కువ ఆదాయం మరియు మధ్య-ఆదాయ దేశాలలో నియోనాటల్ ఎన్సెఫలోపతి తర్వాత అల్పోష్ణస్థితి చికిత్స వలన 18 నెలల వద్ద మరణం లేదా వైకల్యం యొక్క సమ్మిళిత ఫలితాన్ని తగ్గించలేదు, కాని మరణాలు గణనీయంగా పెరిగాయి. తృతీయ నియోనాటల్ ఇంటెన్సివ్ కేర్ సదుపాయాలు అందుబాటులో ఉన్నప్పటికీ, తక్కువ ఆదాయ మరియు మధ్య ఆదాయ దేశాలలో నియోనాటల్ ఎన్సెఫలోపతికి చికిత్సగా చికిత్సా అల్పోష్ణస్థితిని అందించకూడదు.

నిధులు

నేషనల్ ఇన్స్టిట్యూట్ ఫర్ హెల్త్ రీసెర్చ్, గార్ఫీల్డ్ వెస్టన్ ఫౌండేషన్ మరియు బిల్ & మెలిండా గేట్స్ ఫౌండేషన్.
