# Supplementary material for: Hypothermia for moderate or severe neonatal encephalopathy in low-income and middle-income countries (HELIX): a randomised controlled trial in India, Sri Lanka, and Bangladesh
Source: Lancet Glob Health. 2021 Aug 3;9(9):e1273–85. doi: 10.1016/S2214-109X(21)00264-3 (PMC8371331; doi:10.1016/S2214-109X(21)00264-3)
Supplement: Kannada translation of the abstract [file mmc4.pdf]

THE LANCET
Global Health

Supplementary appendix 4

This translation in Kannada was submitted by the authors and we reproduce it as supplied. It has not been peer reviewed. *The Lancet*'s editorial processes have only been applied to the original in English, which should serve as reference for this manuscript.

'[ಕನ್ನಡ ಭಾಷೆಯ] ಈ ಅನುವಾದವನ್ನು ಲೇಖಕರು ಸಲ್ಲಿಸಿದ್ದಾರೆ ಮತ್ತು ನಾವು ಅದನ್ನು ಸರಬರಾಜು ಮಾಡಲಾಗಿರುವಂತೆತೆ ಪುನರುತ್ಪಾದಿಸುತ್ತಿದ್ದೇವೆ. ಇದನ್ನು ಸಮಾನಸ್ಕಂದರು ಪರಿಶೀಲಿಸಿಲ್ಲ. ಲ್ಯಾನ್ಸೆಟ್‌ನ ಸಂಪಾದಕೀಯ ಪ್ರಕ್ರಿಯೆಗಳನ್ನು ಇಂಗ್ಲಿಷ್‌ನಲ್ಲಿನ ಮೂಲಕ್ಕೆ ಮಾತ್ರ ಅನ್ವಯಿಸಲಾಗಿದೆ, ಅದು ಈ ಹಸ್ತಪ್ರತಿಗೆ ಉಲ್ಲೇಖವಾಗಿ ಕಾರ್ಯನಿರ್ವಹಿಸುತ್ತದೆ. 'ಕಕ

Supplement to: Thayyil S, Pant S, Montaldo P, et al. Hypothermia for moderate or severe neonatal encephalopathy in low-income and middle-income countries (HELIX): a randomised controlled trial in India, Sri Lanka, and Bangladesh. *Lancet Glob Health* 2021; published online Aug 3. http://dx.doi.org/10.1016/S2214-109X(21)00264-3.

ಸಾರಾಂಶ:

ಹಿನ್ನೆಲೆ:

ಚಿಕಿತ್ಸಕ ಲಘೂಷ್ಣತೆ ಹೆಚ್ಚಿನ ಆದಾಯದ ದೇಶಗಳಲ್ಲಿ ನವಜಾತ ಎನ್ಸೆಫಲೋಪತಿಯ ನಂತರ ಸಾವು ಅಥವಾ ಅಂಗವೈಕಲ್ಯವನ್ನು ಕಡಿಮೆಗೊಳಿಸಿದರೂ, ಕಡಿಮೆ-ಆದಾಯ ಮತ್ತು ಮಧ್ಯಮ-ಆದಾಯದ ದೇಶಗಳಲ್ಲಿ ಅದರ ಸುರಕ್ಷತೆ ಮತ್ತು ಪರಿಣಾಮಕಾರಿತ್ವವು ಸ್ಪಷ್ಟವಾಗಿಲ್ಲ. ದಕ್ಷಿಣ ಏಷ್ಯಾದಲ್ಲಿ ನವಜಾತ ಎನ್ಸೆಫಲೋಪತಿಯ ನಂತರ ಚಿಕಿತ್ಸಕ ಲಘೂಷ್ಣತೆ ಸಾವು ಕಡಿಮೆಯಾಗುತ್ತದೆಯೇ ಅಥವಾ ಬೆಂಬಲ ಅಥವಾ ತೀವ್ರವಾದ ಆರೈಕೆಯೊಂದಿಗೆ ತೀವ್ರವಾದ ಅಂಗವೈಕಲ್ಯವನ್ನು ಪರೀಕ್ಷಿಸಲು ನಾವು ಗುರಿ ಹೊಂದಿದ್ದೇವೆ.

ವಿಧಾನಗಳು

ನಾವು ಏಳು ತೃತೀಯ ನವಜಾತ ತೀವ್ರ ನಿಗಾ ಘಟಕಗಳಲ್ಲಿ (i.e ಭಾರತ, ಶ್ರೀಲಂಕಾ ಮತ್ತು ಬಾಂಗ್ಲಾದೇಶ) ರಾಂಡಮಾಯ್ಜ್ಡ್ ನಿಯಂತ್ರಿತ ಪ್ರಯೋಗವನ್ನು ಮಾಡಿದ್ದೇವೆ. ಮಧ್ಯಮ ಅಥವಾ ತೀವ್ರವಾದ ನವಜಾತ ಎನ್ಸೆಫಲೋಪತಿಯೊಂದಿಗೆ 36 ವಾರಗಳ ಗರ್ಭಾವಸ್ಥೆಯಲ್ಲಿ ಅಥವಾ ನಂತರ ಜನಿಸಿದ ಶಿಶುಗಳನ್ನು ನಾವು ದಾಖಲಿಸಿದ್ದೇವೆ ಮತ್ತು 5 ನಿಮಿಷ ವಯಸ್ಸಿನಲ್ಲಿ ಮುಂದುವರಿದ ಪುನರುಜ್ಜೀವನದ ಅಗತ್ಯತೆ ಅಥವಾ 5 ನಿಮಿಷ ವಯಸ್ಸಿನಲ್ಲಿ 6 ಕ್ಕಿಂತ ಕಡಿಮೆ ಎಪಿಗರ್ ಸ್ಕೋರ್ (ಆಸ್ಪತ್ರೆಯಲ್ಲಿ ಜನಿಸಿದ ಶಿಶುಗಳಿಗೆ), ಅಥವಾ ಎರಡೂ, ಅಥವಾ 5 ನಿಮಿಷ ವಯಸ್ಸಿನವರೆಗೆ ಅಳುವ ಅನುಪಸ್ಥಿತಿ (ಮನೆಯಲ್ಲಿ ಜನಿಸಿದ ಶಿಶುಗಳಿಗೆ). ವೆಬ್-ಆಧಾರಿತ ರಾಂಡಮಾಯ್ಜ್ಡ್ ವ್ಯವಸ್ಥೆಯನ್ನು ಬಳಸಿಕೊಂಡು, ನಾವು ಶಿಶುಗಳನ್ನು ಇಡೀ ದೇಹದ ಲಘೂಷ್ಣತೆ (33 · 5 ° C) ಅನ್ನು 72 ಗಂಟೆ ಸರ್ವೋ-ನಿಯಂತ್ರಿತ ಕೂಲಿಂಗ್ ಸಾಧನವನ್ನು ಬಳಸಿ ಅಥವಾ ಸಾಮಾನ್ಯ ಆರೈಕೆಗೆ (ನಿಯಂತ್ರಣ ಗುಂಪು), ಜನನದ 6 ಗಂಟೆಯೊಳಗೆ ನಿಯೋಜಿಸಿದ್ದೇವೆ. ಎಲ್ಲಾ ನೇಮಕಾತಿ ತಾಣಗಳಲ್ಲಿ ಆಕ್ರಮಣಕಾರಿ ವಾತಾಯನ, ಹೃದಯರಕ್ತನಾಳದ ಬೆಂಬಲ ಮತ್ತು 3 ಟೆಸ್ಲಾ ಎಂಆರ್ಐ ಸ್ಕ್ಯಾನರ್‌ಗಳು ಮತ್ತು ಸ್ಪೆಕ್ಟ್ರೋಸ್ಕೋಪಿಗೆ ಪ್ರವೇಶಿಸಲು ಸೌಲಭ್ಯಗಳಿವೆ. ಹಸ್ತಕ್ಷೇಪದ ಮರೆಮಾಚುವಿಕೆ ಸಾಧ್ಯವಾಗಲಿಲ್ಲ, ಆದರೆ ಮ್ಯಾಗ್ನೆಟಿಕ್ ರೆಸೋನೆನ್ಸ್ ಬಯೋಮಾರ್ಕರ್ ವಿಶ್ಲೇಷಣೆ ಮತ್ತು ನ್ಯೂರೋ ಡೆವಲಪ್ಮೆಂಟಲ್ ಫಲಿತಾಂಶದ ಮೌಲ್ಯಮಾಪನಗಳಲ್ಲಿ ಭಾಗಿಯಾಗಿರುವವರನ್ನು ಹಂಚಿಕೆಗೆ ಮರೆಮಾಡಲಾಗಿದೆ. ಪ್ರಾಥಮಿಕ ಫಲಿತಾಂಶವೆಂದರೆ 18-22 ತಿಂಗಳುಗಳಲ್ಲಿ ಸಾವಿನ ಅಥವಾ ಮಧ್ಯಮ ಅಥವಾ ತೀವ್ರ ಅಂಗವೈಕಲ್ಯದ ಸಂಯೋಜಿತ ಅಂತಿಮ ಬಿಂದು, ಇದನ್ನು ಬೇಲಿ ಸ್ಕೇಲ್ಸ್ ಆಫ್ ಶಿಶು ಅಭಿವೃದ್ಧಿ (ಆವೃತ್ತಿ 3) ಮತ್ತು ವಿವರವಾದ ನರವೈಜ್ಞಾನಿಕ ಪರೀಕ್ಷೆಯಿಂದ ನಿರ್ಣಯಿಸಲಾಗುತ್ತದೆ.

ಚಿಕಿತ್ಸೆಯ ಉದ್ದೇಶದಿಂದ ವಿಶ್ಲೇಷಣೆ ಆಗಿತ್ತು. ಈ ಪ್ರಯೋಗವನ್ನು ಕ್ಲಿನಿಕಲ್ ಟ್ರಯಲ್ಸ್.ಗೊವ್‌ನಲ್ಲಿ ನೋಂದಾಯಿಸಲಾಗಿದೆ NCT02387385.

ಸಂಶೋಧನೆಗಳು

ಆಗಸ್ಟ್ 15, 2015 ಮತ್ತು ಫೆಬ್ರವರಿ 15, 2019 ರ ನಡುವೆ 2296 ಸಂಭಾವ್ಯ ಅರ್ಹ ಶಿಶುಗಳನ್ನು ನಾವು ಪರೀಕ್ಷಿಸಿದ್ದೇವೆ, ಅವರಲ್ಲಿ 576 ಶಿಶುಗಳು ಸೇರ್ಪಡೆಗೊಳ್ಳಲು ಅರ್ಹರಾಗಿದ್ದಾರೆ. ಹೊರಗಿಟ್ಟ ನಂತರ, ನಾವು 408 ಅರ್ಹ ಶಿಶುಗಳನ್ನು ನೇಮಿಸಿಕೊಂಡಿದ್ದೇವೆ ಮತ್ತು ನಾವು ಲಘೂಷ್ಣತೆ ಗುಂಪಿಗೆ 202 ಮತ್ತು ನಿಯಂತ್ರಣ ಗುಂಪಿಗೆ 206 ಅನ್ನು ನಿಯೋಜಿಸಿದ್ದೇವೆ. ಲಘೂಷ್ಣತೆ ಗುಂಪಿನಲ್ಲಿರುವ 202 ಶಿಶುಗಳಲ್ಲಿ 195 (97%) ಮತ್ತು 206 ನಿಯಂತ್ರಣ ಗುಂಪು ಶಿಶುಗಳಲ್ಲಿ 199 (97%) ಗೆ ಪ್ರಾಥಮಿಕ ಫಲಿತಾಂಶದ ಡೇಟಾ ಲಭ್ಯವಿದೆ. ಲಘೂಷ್ಣತೆ ಗುಂಪಿನಲ್ಲಿ 98 (50%) ಶಿಶುಗಳು ಮತ್ತು ನಿಯಂತ್ರಣ ಗುಂಪಿನಲ್ಲಿ 94 (47%) ಶಿಶುಗಳು ಸತ್ತರು ಅಥವಾ ಮಧ್ಯಮ ಅಥವಾ ತೀವ್ರ ಅಂಗವೈಕಲ್ಯ ಹೊಂದಿದ್ದರು (ಅಪಾಯ ಅನುಪಾತ 1 · 06; 95% ಸಿಐ 0 · 87–1 · 30; ಪು = 0 · 55). ಲಘೂಷ್ಣತೆ ಗುಂಪಿನಲ್ಲಿ 84 ಶಿಶುಗಳು (42%) ಮತ್ತು ನಿಯಂತ್ರಣ ಗುಂಪಿನಲ್ಲಿ 63 (31%; ಪು = 0 · 022) ಶಿಶುಗಳು ಸಾವನ್ನಪ್ಪಿದರು, ಅವರಲ್ಲಿ 72 (36%) ಮತ್ತು 49 (24%; ಪು = 0 · 0087) ನವಜಾತ ಆಸ್ಪತ್ರೆ. ಐದು ಗಂಭೀರ ಪ್ರತಿಕೂಲ ಘಟನೆಗಳು ವರದಿಯಾಗಿವೆ: ಲಘೂಷ್ಣತೆ ಗುಂಪಿನಲ್ಲಿ ಮೂರು (ನ್ಯುಮೋನಿಯಾಕ್ಕೆ ಸಂಬಂಧಿಸಿದ ಒಂದು ಆಸ್ಪತ್ರೆ ಓದುವಿಕೆ, ಒಂದು ಸೆಪ್ಟಿಕ್ ಸಂಧಿವಾತ, ಮತ್ತು ಒಂದು ಸಿರೆಯ ಥ್ರಂಬೋಸಿಸ್ ಎಂದು ಶಂಕಿಸಲಾಗಿದೆ), ಮತ್ತು ನಿಯಂತ್ರಣ ಗುಂಪಿನಲ್ಲಿ ಎರಡು (ಒಂದು ಎಂಆರ್ಐ ಸಮಯದಲ್ಲಿ ಮತ್ತು ಇತರವು ಎಂಡೋಟ್ರಾಶಿಯಲ್ ಟ್ಯೂಬ್‌ನಿಂದಾಗಿ ಎಂಆರ್ಐಗೆ ಸಾಗಣೆಯ ಸಮಯದಲ್ಲಿ ಸ್ಥಳಾಂತರ). ಯಾವುದೇ ಪ್ರತಿಕೂಲ ಘಟನೆಗಳನ್ನು ಅಧ್ಯಯನದ ಹಸ್ತಕ್ಷೇಪಕ್ಕೆ ಕಾರಣವೆಂದು ಪರಿಗಣಿಸಲಾಗಿಲ್ಲ.

ವ್ಯಾಖ್ಯಾನ

ಚಿಕಿತ್ಸಕ ಲಘೂಷ್ಣತೆ ಕಡಿಮೆ ಆದಾಯ ಮತ್ತು ಮಧ್ಯಮ-ಆದಾಯದ ದೇಶಗಳಲ್ಲಿ ನವಜಾತ ಎನ್ಸೆಫಲೋಪತಿಯ ನಂತರ 18 ತಿಂಗಳುಗಳಲ್ಲಿ ಸಾವು ಅಥವಾ ಅಂಗವೈಕಲ್ಯದ ಒಟ್ಟು ಫಲಿತಾಂಶವನ್ನು ಕಡಿಮೆ ಮಾಡಲಿಲ್ಲ, ಆದರೆ ಸಾವನ್ನು ಮಾತ್ರ ಗಮನಾರ್ಹವಾಗಿ ಹೆಚ್ಚಿಸಿತು. ತೃತೀಯ ನವಜಾತ ತೀವ್ರ ನಿಗಾ ಸೌಲಭ್ಯಗಳು ಲಭ್ಯವಿದ್ದರೂ ಸಹ, ಕಡಿಮೆ-ಆದಾಯ ಮತ್ತು ಮಧ್ಯಮ ಆದಾಯದ ದೇಶಗಳಲ್ಲಿ ನವಜಾತ ಎನ್ಸೆಫಲೋಪತಿಗೆ ಚಿಕಿತ್ಸೆಯಾಗಿ ಲಘೂಷ್ಣತೆಯನ್ನು ನೀಡಬಾರದು.

ಧನಸಹಾಯ

ನ್ಯಾಷನಲ್ ಇನ್ಸ್ಟಿಟ್ಯೂಟ್ ಫಾರ್ ಹೆಲ್ತ್ ರಿಸರ್ಚ್, ಗಾರ್ಫೀಲ್ಡ್ ವೆಸ್ಟನ್ ಫೌಂಡೇಶನ್, ಮತ್ತು ಬಿಲ್ & ಮೆಲಿಂಡಾ ಗೇಟ್ಸ್ ಫೌಂಡೇಶನ್.
