# Supplementary material for: Hypothermia for moderate or severe neonatal encephalopathy in low-income and middle-income countries (HELIX): a randomised controlled trial in India, Sri Lanka, and Bangladesh
Source: Lancet Glob Health. 2021 Aug 3;9(9):e1273–85. doi: 10.1016/S2214-109X(21)00264-3 (PMC8371331; doi:10.1016/S2214-109X(21)00264-3)
Supplement: Singhalese translation of the abstract [file mmc5.pdf]

THE LANCET
Global Health

Supplementary appendix 5

This translation in Sinhala was submitted by the authors and we reproduce it as supplied. It has not been peer reviewed. *The Lancet*'s editorial processes have only been applied to the original in English, which should serve as reference for this manuscript.

මෙම සිංහල භාෂා පරිවර්තනය කතුවරුන් විසින් යොමුකරන ලද අතර අපි එය සපයා ඇති පරිදි ඉදිරිපත් කරන්නෙමු. මේ සඳහා තුල්‍යසමාලෝචනයක් සිදුකර නොමැත. ලැන්සෙට්හි සංස්කරණ ක්‍රියාවලීන් යෙදී ඇත්තේ මේ සඳහා යොමු කිරීමක් වන මෙහි ඉංග්‍රීසි මුල් පිටපතට පමණි.

Supplement to: Thayyil S, Pant S, Montaldo P, et al. Hypothermia for moderate or severe neonatal encephalopathy in low-income and middle-income countries (HELIX): a randomised controlled trial in India, Sri Lanka, and Bangladesh. *Lancet Glob Health* 2021; published online Aug 3. http://dx.doi.org/10.1016/S2214-109X(21)00264-3.

සාරාංශය

පසුබිම

ඉහල ආදායම් ලාභී රටවල (H.M.I.Cs) නවජන්ම මස්තිෂ්ක ආසාදනයෙන් පසුව හටගන්නා මරණ හා අබාධිතතාවයන් ශරීර උෂ්ණත්වය ඌන කිරීමේ ප්‍රතිකර්මය (therapeutic hypothermia) මගින් අවම කරගනු ලබේ. නමුත් එම ක්‍රමය අඩු සහ මධ්‍යම ආදායම්ලාභී රටවල (L .M .I .Cs) සිදු කිරීම කෙතරම් දුරට ආරක්ෂිත හෝ කාර්යක්ෂම දැයි මෙතෙක් පැහැදිලි නැත.

ක්‍රමවේදය

ඉන්දියාව ,ශ්‍රී ලංකාව සහ බංග්ලාදේශය යන රටවල් කිහිපයක තෘතීයික නවජන්ම දැඩි සත්කාර ඒකක 7 ක ඒකාබද්ධතාවයකින් යුතුව සිදුකරන ලද බහු දේශී, විවෘත නාමික, අහඹු ලෙස පාලනය කළ අත්හදා බැලීම් (multi-country open label randomized controlled trial) ඔස්සේ අප විසින් මෙම පරීක්ෂණය සිදු කළෙමු. මෙම පර්යේෂණය සඳහා, සති 36 හෝ වැඩි ගර්භණී කාලයකින් පසුව උපත ලැබූ මධ්‍යම හෝ අධික නවජන්ම මස්තිෂ්ක ආසාදන තත්වය සහිත වූ සහ උපතින් මිනිත්තු 5ක අවදියේදි අඛණ්ඩ පුනර්ජීව (resuscitation) සහායට ලක්ව හෝ ඇප්ගාර් (Apgar) අගය උපතින් මිනිත්තු 5 දී 6ට අඩු (රෝහලේ උපත ලද දරුවන්) හෝ ඉහත සඳහන් ලක්ෂණ දෙකම සහිත වීම හෝ ඉපදී මිනිත්තු 5ක් තුළදී නොහඩා සිටිය (නිවසෙදී උපත ලද දරුවන්) ළදරුවන් අප විසින් පර්යේෂණය සඳහා බඳවා ගන්නා ලදී. මෙම ළදරුවන් වෙබ් පදනම් කරගත් අහඹු ලෙස වෙන් කරන ක්‍රියවලියක් (web-based randomization system) භාවිතයෙන් කාණ්ඩ ගත කරන ලදී. උපත් වේලවේ සිට පැය 6ක කාලයක් තුළදී සර්වෝ-පාලිත සිසිලන උපකරණයක් (servo-controlled cooling device) භාවිතයෙන් පැය 72ක් පුරා පූර්ණ ශරීර උෂ්ණත්වය ඌන කිරීමේ ප්‍රතිකර්මයට (35.5 C) හෝ සුපුරුදු ප්‍රතිකර්ම ක්‍රමය (පාලක කණ්ඩායම) සඳහා යොමු කරන ලදී. මෙම පර්යේෂණ මධ්‍යස්ථානයන්හි කෘතීම ස්වසන අධාරක පද්ධති (invasive ventilation) සහ හෘද වාහිනී ආධාරක පහසුකම් සහිත විය. තවද 3 ටෙස්ලා චුම්භක අනුනාද රූපක (3 Tesla MRI scanner) සහ වර්ණාවලීක්ෂය (spectroscopy) යන පහසුකම් මෙම සියලු මධ්‍යස්ථානයන් සඳහා ලබාගත හැකි විය. මැදිහත් වීම සිදු කිරිමේදී අනු කාණ්ඩ දෙක පිළිබඳව අනාවරණය සිදු කළද චුම්භක අනුනාද ජෛව සලකුණු විශ්ලේෂණය සහ ස්නායු සංවර්ධනය ඇගයීම් සිදුකල පර්යේෂකයින්ට, පර්යේෂණ අනු කාණ්ඩ පිළිබඳව අනාවරණය නොකරන ලදී.

මෙම පර්යේෂණයේ ප්‍රාථමික ප්‍රතිඵලය ලෙස සලකනු ලැබුවේ මරණය හෝ මාස 18-22 වයසේදී, බේලි ළදරුවන්ගේ සහ කුඩා දරුවන්ගේ සංවර්ධන පරිමාණයට (Bayley Scales of Infant and Toddler Development) (3 වන පිටපත) අනුව අගයන ලද සහ විස්තරාත්මකව ස්නායු පද්ධතිය පරික්ෂාවට ලක් කිරීමෙන් තහවුරු කරන ලද, මධ්‍යම හෝ අධික ආබාධිතතාවයක් යන එකාබද්ධ අවසාන ඉලක්කයන්ය. ප්‍රතිඵල විශ්ලේෂණය පදනම් වූයේ ප්‍රතිකර්මය සඳහා වූ අභිප්‍රායයන් මතය. මෙම අත් හදා බැලීම ClinicalTrials.gov, NCT02387385 ලෙස ලියා ලියාපදිංචි වී ඇත.

සොයා ගැනීම්

සුදුසුකම් සහිත ළදරුවන් 2296ක් වසර 2015 අගෝස්තු 15 වනදා සිට 2019 පෙබරවාරි 15 වනදා දක්වා පිරික්සීමට ලක් කිරීමෙන් අනතුරුව ළදරුවන් 576 දෙනෙකු පර්යේෂණයට අන්තර්ගත කිරීමට යෝග්‍ය ලෙස හඳුනා ගන්නා ලදී. බැහැර කිරීම් අවසානයේ යෝග්‍යතා සහිත ළදරුවන් 408ක් බඳවා ගත් අතර ශරීර උෂ්ණත්වය ඌන කිරීමේ කාණ්ඩයට ළදරුවන් 202ක් ද පාලිත කාණ්ඩයට 206ක් ද වෙන් කරන ලදී. උෂ්ණත්වය ඌන කිරීමේ කණ්ඩායමට අයත් ළදරුවන් 202 අතුරින් 195 (97%) සහ පාලක කාණ්ඩයට අයත් ළදරුවන් 206 අතුරින් 199 (97%) ළදරුවන්ගේ ප්‍රාථමික ප්‍රතිඵල දත්ත ලබාගත හැකිය. ශරීර උෂ්ණත්වය ඌන කිරීමේ ප්‍රතිකාර ලැබූ ළදරුවන් 98 (50%) සහ පාලක කන්ඩායමේ 94 (47%) දෙනකු , මරණය හෝ මධ්‍යස්ථ හෝ අධික ආබාධිත තත්වයට ලක්විය (අවදානම් අනුපාතය (RR) 1.06; 95% විශ්වාසනීය අන්තරයන් (CI) 0.87 සිට 1.30; (p =0.55) දක්වා. ශරීර උෂ්ණත්වය ඌන කිරීමේ ප්‍රතිකාර කාණ්ඩයේ ළදරුවන් අසූ හතර (84) දෙනෙකු (42%) සහ පාලක කණ්ඩායමේ ළදරුවන් 63 (31%; p = 0.022) මිය ගියහ. ඒ අතුරින් ළදරුවන් 72 (36%) සහ 49 (24%; p= 0.0087) රෝහල තුළදී මිය ගියහ.

බරපතල අහිතකර සිදුවීම් පහක් වාර්ථා විය: ශරීර උෂ්ණත්වය ඌණ කිරීමේ කණ්ඩායමෙන් තුනක්ද (නියුමෝනියාව හේතුවෙන් නැවත රෝහල්ගත වීමක් සහිත එක ළදරුවෙකු, සන්ධියකට විශබීජ ඇතුලුවීමෙන් හටගත් සෙප්ටික් ආතරයිටීස් තත්වය සහිත එක ළදරුවෙකු සහ රුධිරය කැටි ගැසීම අනුමාන කළ තව ළදරුවෙකු) පාලක කණ්ඩායමෙන් දෙකක්ද (MRI පරීක්ෂණය කරන වේලාව තුළදී ඔක්සිජන් මද වීම සහ MRI පර්යේෂණය සඳහා ගමන් ගන්නා විට ශ්වසන අධාර නාලය (endotracheal tube) විස්ථාපනය වීම) වාර්ථා විය. කිසිදු අහිතකර සිදුවීමක් පර්යේෂණ මැදිහත් වීම හේතුවෙන් සිදු නොවන ලදී.

අර්ථ නිරූපණය

අඩු සහ මධ්‍යම ආදායම් ලාභී රටවල (LMICs), ශරීර උෂ්ණත්වය ඌන කිරීමේ ප්‍රතිකර්මය (therapeutic hypothermia) මගින් නවජන්ම මස්තිෂ්ක ආසාදනය (neonatal encephalopathy) හේතුවෙන් හටගන්නා මරණය හෝ මාස 18දී අබාධිතතාවය යන එකාබද්ධිත ප්‍රතිඵලය අවම කිරීමක් දක්නට නොලැබුනි. එනමුත් සැලකිය යුතු ලෙස මරණ සංඛ්‍යාව පමණක් වැඩිවිය. එහෙයින් අඩු සහ මධ්‍යම ආදායම් ලාභී රටවල නවජන්ම දැඩි සත්කාර පහසුකම් ඇති අවස්ථාවන්වලදී වුවද නවජන්ම මස්තිෂ්ක ආසාධනය සහිත ළදරුවන් සඳහා ශරීර උෂ්ණත්වය ඌන කිරීමේ ප්‍රතිකර්මය ලබා දීම සිදු නොකල යුතුය.
