# Supplementary material for: Hypothermia for moderate or severe neonatal encephalopathy in low-income and middle-income countries (HELIX): a randomised controlled trial in India, Sri Lanka, and Bangladesh
Source: Lancet Glob Health. 2021 Aug 3;9(9):e1273–85. doi: 10.1016/S2214-109X(21)00264-3 (PMC8371331; doi:10.1016/S2214-109X(21)00264-3)
Supplement: Tamil translation of the abstract [file mmc6.pdf]

THE LANCET
Global Health

Supplementary appendix 6

This translation in Tamil was submitted by the authors and we reproduce it as supplied. It has not been peer reviewed. *The Lancet*'s editorial processes have only been applied to the original in English, which should serve as reference for this manuscript.

தமிழில் இந்த மொழிபெயர்ப்பு ஆசிரியர்களால் சமர்ப்பிக்கப்பட்டது, நாங்கள் அதை வழங்கியபடி மீண்டும் உருவாக்குகிறோம்.இது மதிப்பாய்வு செய்யப்படவில்லை. லான்செட்டின் தலையங்க செயல்முறைகள் ஆங்கிலத்தில் அசலுக்கு மட்டுமே பயன்படுத்தப்பட்டுள்ளன, இது இந்த கையெழுத்துப் பிரதிக்கான குறிப்பாக செயல்பட வேண்டும்.

Supplement to: Thayyil S, Pant S, Montaldo P, et al. Hypothermia for moderate or severe neonatal encephalopathy in low-income and middle-income countries (HELIX): a randomised controlled trial in India, Sri Lanka, and Bangladesh. *Lancet Glob Health* 2021; published online Aug 3. http://dx.doi.org/10.1016/S2214-109X(21)00264-3.

சுருக்கம்

பின்னணி

உயர் வருமான நாடுகளில் பிறந்த குழந்தை என்செபலோபதிக்குப் பிறகு மரணம் அல்லது இயலாமை, சிகிச்சையளிக்கும் தாழ்வெப்பநிலையால் குறைக்கிறது என்றாலும், குறைந்த மற்றும் நடுத்தர வருமான நாடுகளில் (எல்எம்ஜசி) அதன் பாதுகாப்பு மற்றும் செயல்திறன் தெளிவாக இல்லை. தெற்காசியாவில் பிறந்த குழந்தை என்செபலோபதிக்குப் பிறகு சிகிச்சையளிக்கும் தாழ்வெப்பநிலை உடன் உகந்த ஆதரவான தீவிர சிகிச்சையால் மரணம் அல்லது மிதமான அல்லது கடுமையான இயலாமை ஆகியவற்றைக் குறைக்கிறதா என்பதை ஆராய்வதை நோக்கமாகக் கொண்டோம்.

முறைகள்

இந்தியா, இலங்கை மற்றும் வங்கதேசத்தில் உள்ள 7 மூன்றாம் நிலை பிறந்த குழந்தை தீவிர சிகிச்சைப் பிரிவுகளில் பன்னாட்டு திறந்த லேபிள் சீரற்ற கட்டுப்பாட்டு சோதனையை நாங்கள் நடத்தினோம். நாங்கள் சேர்த்துள்ள குழந்தைகள், பிரசவத்தில் கர்ப்பகால வயது 36 அல்லது அதுக்கு மேல் உள்ள மிதமான அல்லது தீவீர என்செபாலோபதி உள்ள குழந்தைகள் மற்றும் பிறந்து 5 நிமிடமே ஆன தொடர்ந்து உயிரூட்டுதல் சிகிச்சை தேவைபடும் குழந்தை அல்லது 5 நிமிட வயதில் 6 க்கும் குறைவான அப்கார் மதிப்பெண்(மருத்துவமனையில் பிறந்த குழந்தைகளுக்கு) அல்லது இரண்டும், அல்லது 5 நிமிட வயதிலேயே அழாதது (வீட்டில் பிறந்த குழந்தைகளுக்கு). இணைய அடிப்படையிலான சீரற்றமயமாக்கல் முறையைப் பயன்படுத்தி, குழந்தைகளை முழு உடல் தாழ்வெப்பநிலை (33 · 5 ° C) பெறும் குழுவில் 72 மணிநேரத்திற்கு ஒரு சர்வோ-கட்டுப்படுத்தப்பட்ட குளிரூட்டும் கருவியைப் பயன்படுத்தி அல்லது வழக்கமான கவனிப்புக்கு (கட்டுப்பாட்டு குழு), பிறந்த 6 மணி நேரத்திற்குள் ஒதுக்கினோம். அனைத்து ஆட்சேர்ப்பு தளங்களிலும் இன்வாஸிவ் வென்டிலேஷன், கார்டியோ வாஸ்குலர் சப்போர்ட் மற்றும் 3 டெஸ்லா எம்ஆர்ஐ ஸ்கேனர்கள் மற்றும் ஸ்பெக்ட்ரோஸ்கோபி ஆகியவற்றை அணுகுவதற்கான வசதிகள் இருந்தன. தலையீட்டை மறைப்பது சாத்தியமில்லை, ஆனால் காந்த அதிர்வு பயோமார்க்ஸ் பகுப்பாய்வு மற்றும் நரம்பியல் வளர்ச்சி விளைவு மதிப்பீடுகளில் ஈடுபட்டவர்கள் ஒதுக்கீட்டில் மறைக்கப்பட்டனர். முதன்மை விளைவு 18-22 மாதங்களில் மரணம் அல்லது மிதமான அல்லது கடுமையான இயலாமை ஆகியவற்றின் ஒருங்கிணைந்த இறுதிப் புள்ளியாகும், இது பேய்லி ஸ்கேல்ஸ் ஆஃப் சிசு டெவலப்மென்ட் (பதிப்பு 3) மற்றும் விரிவான நரம்பியல் பரிசோதனை ஆகியவற்றால் மதிப்பிடப்பட்டது. சிகிச்சையளிக்கும் நோக்கத்தினால் பகுப்பாய்வு செய்யப்பட்டது. இந்த சோதனை ClinicalTrials.gov, NCT02387385 இல் பதிவு செய்யப்பட்டுள்ளது.

கண்டுபிடிப்புகள்

ஆகஸ்ட் 15, 2015 மற்றும் பிப்ரவரி 15, 2019 க்கு இடையில் 2296 தகுதி வாய்ந்த குழந்தைகளை நாங்கள் திரையிட்டோம், அவர்களில் 576 குழந்தைகளும் சேர்க்க தகுதியுடையவர்கள். விலக்குகளுக்குப் பிறகு, நாங்கள் 408 தகுதி வாய்ந்த குழந்தைகளை ஒதுக்கினோம் நாங்கள் 202 ஐ தாழ்வெப்பநிலை குழுவிற்கும் 206 கட்டுப்பாட்டு குழுவிற்கும் ஒதுக்கினோம். தாழ்வெப்பநிலை குழுவில் உள்ள 202 குழந்தைகளில் 195 (97%) மற்றும் 206 கட்டுப்பாட்டு குழு குழந்தைகளில் 199 (97%) முதன்மை விளைவு தரவு கிடைத்தது. தாழ்வெப்பநிலை குழுவில் 98 (50%) குழந்தைகளும், கட்டுப்பாட்டு குழுவில் 94 (47%) குழந்தைகளும் இறந்தனர் அல்லது மிதமான அல்லது கடுமையான இயலாமை கொண்டிருந்தனர் (ஆபத்து விகிதம் 1·06; 95% சிஐ 0·87-1 · 30; ப = 0·55). தாழ்வெப்பநிலை குழுவில் 84 குழந்தைகளும் (42%) மற்றும் கட்டுப்பாட்டு குழுவில் 63 (31%; ப = 0·022) குழந்தைகளும் இறந்தனர், அவர்களில் 72 (36%) மற்றும் 49 (24%; ப = 0·0087) மருத்துவமனையில் உள்ள பிறந்த குழந்தை தீவிர சிகிச்சை பிரிவில் அனுமதிக்கப்படும்பொழுதே இறந்தனர். ஐந்து கடுமையான பாதகமான நிகழ்வுகள் பதிவாகியுள்ளன: தாழ்வெப்பநிலை குழுவில் மூன்று (நிமோனியா தொடர்பான ஒரு மருத்துவமனை வாசிப்பு, ஒரு செப்டிக் ஆர்த்ரிடிஸ், மற்றும் ஒரு வீனஸ் த்ரோம்போசிஸ் என சந்தேகிக்கப்படுகிறது), மற்றும் கட்டுப்பாட்டுக் குழுவில் இரண்டு (ஒன்று எம்.ஆர்.ஐ.யின் போது ஆக்ஸிஜன் குறைபாட்டால் தொடர்பானது மற்றும் மற்றொன்று எம்.ஆர்.ஐ.க்கான போக்குவரத்தின் போது எண்டோட்ரோகீயல் குழாய் இடப்பெயர்ச்சி காரணமாக).எந்தவொரு எதிர்மறையான நிகழ்வுகளும் ஆய்வு தலையீட்டோடு தொடர்புடையதாக கருதப்படவில்லை

விளக்கம்

சிகிச்சையளிக்கும் தாழ்வெப்பநிலை குறைந்த வருமானம் மற்றும் நடுத்தர வருமான நாடுகளில் பிறந்த குழந்தை என்செபலோபதிக்குப் பிறகு 18 மாதங்களில் மரணம் அல்லது இயலாமை ஆகியவற்றின் ஒருங்கிணைந்த விளைவைக் குறைக்கவில்லை, ஆனால் இறப்பை மட்டும் கணிசமாக அதிகரித்தது. மூன்றாம் நிலை பிறந்த குழந்தை தீவிர சிகிச்சை வசதிகள் கிடைக்கும்போது கூட, குறைந்த வருமானம் மற்றும் நடுத்தர வருமான நாடுகளில் பிறந்த குழந்தை என்செபலோபதிக்கு சிகிச்சையாக சிகிச்சையளிக்கும் தாழ்வெப்பநிலை வழங்கப்படக்கூடாது

நிதி

தேசிய சுகாதார ஆராய்ச்சி நிறுவனம், கார்பீல்ட் வெஸ்டன் அறக்கட்டளை மற்றும் பில் & மெலிண்டா கேட்ஸ் அறக்கட்டளை.
