# Supplementary material for: Hypothermia for moderate or severe neonatal encephalopathy in low-income and middle-income countries (HELIX): a randomised controlled trial in India, Sri Lanka, and Bangladesh
Source: Lancet Glob Health. 2021 Aug 3;9(9):e1273–85. doi: 10.1016/S2214-109X(21)00264-3 (PMC8371331; doi:10.1016/S2214-109X(21)00264-3)
Supplement: Marathi translation of the abstract [file mmc7.pdf]

THE LANCET
Global Health

Supplementary appendix 7

This translation in Marathi was submitted by the authors and we reproduce it as supplied. It has not been peer reviewed. *The Lancet*'s editorial processes have only been applied to the original in English, which should serve as reference for this manuscript.

'हे भाषांतर [इथे भाषेचा समावेश करा] लेखकांनी सादर केले होते आणि आम्ही जसे मिळाले तसे ते प्रकाशित करत आहोत. याचे सावधानीपूर्वक परीक्षण केले गेले नाही. लॅन्सेटच्या संपादकीय प्रक्रिया फक्त इंग्रजीतील मूळ लेखावरच लागू केल्या आहेत, ज्याचा या हस्तलिखिताकरिता संदर्भ म्हणून वापर केला पाहिजे.'

Supplement to: Thayyil S, Pant S, Montaldo P, et al. Hypothermia for moderate or severe neonatal encephalopathy in low-income and middle-income countries (HELIX): a randomised controlled trial in India, Sri Lanka, and Bangladesh. *Lancet Glob Health* 2021; published online Aug 3. http://dx.doi.org/10.1016/S2214-109X(21)00264-3.

सारांश

संदर्भ

उच्च उत्पन्न असलेल्या देशांमध्ये नवजात एन्सेफॅलोपॅथीनंतर उपचारात्मक हायपोथर्मियामुळे मृत्यू किंवा अपंगत्व कमी होते, परंतु कमी उत्पन्न आणि मध्यम उत्पन्न असलेल्या देशांमध्ये त्याची सुरक्षा आणि कार्यक्षमता अस्पष्ट आहे.दक्षिण आशियात नवजात एन्सेफॅलोपॅथीनंतर इष्टतम सहाय्यक गहन काळजीसह उपचारात्मक हायपोथर्मिया मृत्यू किंवा मध्यम किंवा गंभीर अपंगत्व कमी करते की नाही हे तपासण्याचे आमचे लक्ष्य आहे.

पद्धती

आम्ही भारत, श्रीलंका आणि बांगलादेशमधील सात तृतीय नवजात गहन काळजीवाहू विभागात एक मल्टी-कंट्री ओपन-लेबल, यादृच्छिक नियंत्रित चाचणी केली. आम्ही मध्यम किंवा गंभीर नवजात एन्सेफॅलोपॅथीसह गर्भधारणेच्या 36 आठवड्यांनंतर किंवा नंतर जन्मलेल्या नवजात मुलांची नोंदणी केली आणि 5 व्या वर्षी किंवा रुग्णालयात जन्मलेल्या मुलांसाठी 6 वर्षांपेक्षा कमी वयाच्या अपगर स्कोअरसाठी सतत पुनरुत्थान आवश्यक होता, किंवा दोन्ही, किंवा 5 मिनिटांनी (घरी जन्मलेल्या मुलांसाठी) रडण्याची अनुपस्थिती.वेब-बेस्ड रँडमाइझेशन सिस्टमचा वापर करून, आम्ही जन्माच्या 6 तासांच्या आत, सर्वो-नियंत्रित शीतलक डिव्हाइसचा वापर करून 72 तासांसाठी संपूर्ण शरीर हायपोथर्मिया (33 · 5 डिग्री सेल्सियस) प्राप्त करणा एका गटामध्ये अर्भकांचे वाटप केले.सर्व भरती साइट्समध्ये आक्रमक वायुवीजन, हृदय व रक्तवाहिन्यासंबंधी आधार आणि 3 टेस्ला एमआरआय स्कॅनर आणि स्पेक्ट्रोस्कोपीमध्ये प्रवेश करण्याची सुविधा होती. हस्तक्षेपाचे मुखवटा काढून टाकणे शक्य नव्हते, परंतु चुंबकीय अनुनाद बायोमार्कर विश्लेषण आणि न्यूरो डेव्हलपेलमेंटल परिणामांच्या मूल्यांकनात गुंतलेल्या लोकांचे वाटप करण्यात मुखवटा घातलेले होते. प्राथमिक परिणाम हा मृत्यू किंवा मध्यम किंवा गंभीर अपंगत्वाचा संयुक्त शेवटचा बिंदू होता, १-२२ महिन्यांत, शिशु विकासाचे बायले स्केल (आवृत्ती)) आणि सविस्तर न्यूरोलॉजिकल तपासणीद्वारे मूल्यांकन.विश्लेषण उपचार करण्याचा हेतू होता. ही चाचणी ClinicalTrials.gov, NCT02387385 वर नोंदणीकृत आहे.

निष्कर्ष

आम्ही 15 ऑगस्ट 2015 ते 15 फेब्रुवारी 2019 दरम्यान 2296 संभाव्य अर्भकांचे स्क्रिनिंग केले, त्यापैकी 576 शिशु समावेशास पात्र होते.बहिष्कारानंतर आम्ही 408 पात्र अर्भकांची भरती केली आणि आम्ही हायपोथर्मिया ग्रुपला 202 आणि कंट्रोल ग्रुपला 206 नेमले.हायपोथर्मिया ग्रुपमधील 202 शिशुंपैकी 195 (97%) आणि 206 कंट्रोल ग्रुप शिशुंपैकी 199 (97%) प्राथमिक निकाल डेटा उपलब्ध होता. हायपोथर्मिया ग्रुपमधील 98 (50%) आणि नियंत्रण गटातील 94 (47%) लहान मुले मरण पावली किंवा मध्यम किंवा गंभीर अपंगत्व (जोखीम प्रमाण 1.06; 95% सीआय 0.87-1.30; पी = 0.55). हायपोथर्मिया ग्रुपमधील 84 शिशु (42%) आणि नियंत्रण गटातील (63 (31%; पी = 0.022) मरण पावले, त्यापैकी 72(36%) आणि 49 (24%; पी = 0.0087) दरम्यान मरण पावले नवजात रुग्णालयात दाखल. पाच गंभीर प्रतिकूल घटना नोंदविण्यात आल्या: हायपोथर्मिया ग्रुपमधील तीन (न्यूमोनिया संबंधित एक हॉस्पिटल रीडमिशन, एक सेप्टिक आर्थरायटिस आणि एक संशयित शिरासंबंधीचा थ्रोम्बोसिस), आणि दोन कंट्रोल ग्रुपमध्ये (एक एमआरआय दरम्यान असुरक्षिततेशी संबंधित आणि इतर एंडोस्ट्रॅक्शन ट्यूबमुळे एमआरआय वाहतुकीदरम्यान विस्थापन).कोणतीही प्रतिकूल घटना अभ्यासाच्या हस्तक्षेपाशी संबंधित नाहीत.

व्याख्या

अल्प-उत्पन्न आणि मध्यम उत्पन्न असलेल्या देशांमध्ये नवजात एन्सेफॅलोपॅथी नंतर 18 महिन्यांनंतर उपचारात्मक हायपोथर्मियाने मृत्यू किंवा अपंगत्वाचा एकत्रित परिणाम कमी केला नाही, परंतु केवळ मृत्यूमुळेच लक्षणीय वाढ झाली. अल्प-उत्पन्न आणि मध्यम उत्पन्न असणाऱ्या देशांमध्ये नवजात एन्सेफॅलोपॅथीवर उपचारात्मक हायपोथर्मिया देऊ नये, तर तृतीय स्तरीय नवजात गहन काळजीची सुविधा उपलब्ध असतानाही.

निधी

नॅशनल इन्स्टिट्यूट फॉर हेल्थ रिसर्च, गारफिल्ड वेस्टन फाउंडेशन आणि बिल अँड मेलिंडा गेट्स फाउंडेशन.
