# Supplementary material for: Hypothermia for moderate or severe neonatal encephalopathy in low-income and middle-income countries (HELIX): a randomised controlled trial in India, Sri Lanka, and Bangladesh
Source: Lancet Glob Health. 2021 Aug 3;9(9):e1273–85. doi: 10.1016/S2214-109X(21)00264-3 (PMC8371331; doi:10.1016/S2214-109X(21)00264-3)
Supplement: Bangla translation of the abstract [file mmc8.pdf]

THE LANCET
Global Health

Supplementary appendix 8

This translation in Bangla was submitted by the authors and we reproduce it as supplied. It has not been peer reviewed. *The Lancet*'s editorial processes have only been applied to the original in English, which should serve as reference for this manuscript.

'এই [বাংলায়] অনুবাদটি লেখকরা জমা দিয়েছিলেন এবং এটি যেমনভাবে দেওয়া হয়েছে আমরা সেইভাবেই পুনরায় বর্ণনা করছি। এটি কোনো সমকক্ষ ব্যক্তি পর্যালোচনা করেননি। দ্য ল্যানসেট-এর সম্পাদকীয় প্রক্রিয়াগুলি শুধুমাত্র মূল ইংরেজিতে প্রয়োগ করা হয়েছে, যা এই পাণ্ডুলিপির রেফারেন্স হিসাবে কাজ করবে।'

Supplement to: Thayyil S, Pant S, Montaldo P, et al. Hypothermia for moderate or severe neonatal encephalopathy in low-income and middle-income countries (HELIX): a randomised controlled trial in India, Sri Lanka, and Bangladesh. *Lancet Glob Health* 2021; published online Aug 3. http://dx.doi.org/10.1016/S2214-109X(21)00264-3.

নিম্ন ও মধ্যম আয়ের দেশগুলিতে পেরিন্যাটাল এ্যাসফাইক্সিয়া বা জন্মকালীন শ্বাসকষ্টজনিত রোগে থেরাপিউটিক হাইপোথার্মিয়ার কার্যকারিতা

পটভূমিঃ উচ্চ আয়ের দেশগুলিতে নবজাতক এনসেফালোপ্যাথিতে থেরাপিউটিক হাইপোথার্মিয়া মৃত্যু বা অক্ষমতা কমাতে পারলেও নিম্ন ও মধ্যম আয়ের দেশগুলিতে এর সুরক্ষা ও কার্যকারিতা অস্পষ্ট। সর্বোত্তম নিবিড় যত্নের পাশাপাশি হাইপোথার্মিয়া দক্ষিণ এশিয়ার নবজাতকের এনসেফালোপ্যাথি পরবর্তী মৃত্যু বা মাঝারি বা গুরুতর অক্ষমতা কমাতে পারে কিনা তা পরীক্ষা করাই ছিল আমাদের লক্ষ্য।

গবেষণা কার্যক্রমঃ আমরা ভারত, শ্রীলংকা এবং বাংলাদেশের সাতটি তৃতীয় স্তরের নবজাতক নিবিড় পরিচর্যা কেন্দ্র যুক্ত বহু দেশীয়, ওপেন লেভেল, বিচ্ছিন্নকরণ নিয়ন্ত্রিত গবেষণা ট্রায়াল পরিচালনা করি। আমরা গর্ভকালীন সপ্তাহ ৩৬ বা তার বেশি পূর্ণ হওয়া মাঝারি বা গুরুতর এনসেফালোপ্যাথিতে আক্রান্ত যাদের জন্মের পরবর্তী ৫ মিনিটের বেশি শ্বাসে সহায়তা দরকার হয় অথবা ৫ মিনিট বয়সে আপগার স্কোর ৬ এর নিচে থাকে(হাসপাতালে জন্ম নেয়ার ক্ষেত্রে) অথবা জন্মের ৫ মিনিট পরেও যদি না কাঁদে (বাড়িতে জন্ম নেয়ার ক্ষেত্রে) - সেই সব নবজাতকদের তালিকাভুক্ত করেছি। ওয়েব ভিত্তিক প্রক্রিয়ায় মাধ্যমে আমরা জন্মের ৬ ঘন্টার মধ্যে বিচ্ছিন্ন ভাবে একটি গোষ্ঠীকে নিয়ন্ত্রিত শীতলিকরণ বা হাইপোথার্মিয়া (৭২ ঘন্টা ধরে শরীরের তাপমাত্রা ৩৩.৫^{0} সে বজায় রাখা) অথবা স্বাভাবিক যত্ন (নিয়ন্ত্রণ গোষ্ঠী) এই দুই ভাগে চিকিৎসা অব্যাহত রাখি। সব অংশগ্রহনকারী কেন্দ্রে কৃত্রিম শ্বাস প্রশ্বাস, হৃদযন্ত্রের ক্রিয়া সচল রাখার চিকিৎসা এবং ৩ তেসলা এম আর আই মেশিনের সুবিধা ছিলো। গবেষণায় ব্যবহৃত হস্তক্ষেপটি মাস্কিং করা সম্ভব না হলেও এম আর আই পরীক্ষা এবং স্নায়ু- বিকাশ পর্যবেক্ষণে জড়িত রা গোষ্ঠীর পরিচয় সম্পর্কে অবহিত ছিলেন না। প্রাথমিক ফলাফল হিসেবে আমরা নবজাতকের ১৮ থেকে ২২ মাস বয়সে সম্মিলিত ভাবে মৃত্যু বা মাঝারি বা গুরুতর অক্ষমতা যা বেইলি স্কেল ভার্শন ৩ এবং বিস্তারিত স্নায়ুর পরীক্ষা দ্বারা দেখা হয়। ইন্টেনশন টু ট্রিট প্রক্রিয়ায় সমস্ত বিশ্লেষণ করা হয়। ট্রায়াল রেজি নাম্বার ক্লিনিক্যাল ট্রায়াল. গভ, এন সি টি ০২৩৮৭৩৮৫।

অনুসন্ধানের ফলাফলঃ আমরা ১৫ই আগস্ট ২০১৫ ইং হতে ১৫ই ফেব্রুয়ারি ২০১৯ ইং এর মধ্যে ২২৯৬ সম্ভাব্য যোগ্য নবজাতকের স্ক্রিনিং করেছি, যার মধ্যে ৫৭৬ টি শিশু মূল গবেষণায় অন্তর্ভুক্ত করা হয়েছিল। বাতিলকরনের পরে, আমরা ৪০৮ টি যোগ্য শিশুকে নিয়োগ দেই এবং হাইপোথার্মিয়া গোষ্ঠী তে ২০২ এবং নিয়ন্ত্রণ গোষ্ঠীতে চিকিৎসা অব্যাহত রাখি ২০৬ জন নবজাতককে। হাইপোথার্মিয়া গ্রুপের ২০২ টি শিশুর মধ্যে ১৯৫ (৯৭%) এবং ২০৬ টি নিয়ন্ত্রণ গোষ্ঠীর শিশুদের ১৯৯(৯৭%) জনের প্রাথমিক ফলাফল পাওয়া গিয়েছিল। হাইপোথার্মিয়া পাওয়া

শিশুদের মধ্যে ৯৮(৫০%) এবং নিয়ন্ত্রণ গোষ্ঠীতে ৯৪(৪৭%) শিশুর মৃত্যু বা মাঝারি বা গুরুতর অক্ষমতা দেখা দেয়(ঝুঁকির অনুপাত ১.০৬; ৯৫% সি আই ০.৮৭ থেকে ১.৩০) (পি= ০.৫৫)। হাইপোথার্মিয়া পাওয়া শিশুদের মধ্যে ৮৪(৪২%) এবং নিয়ন্ত্রণ গ্রুপের মধ্যে ৬৩(৩১%; পি = ০.২২) জন মারা যায়, এদের মধ্যে ৭২(৩৬%) এবং ৪৯ জন ই (২৪%; পি = ০.০০৮৭) মারা যায় হাসপাতালে চিকিৎসাধীন অবস্থায়। পাঁচটি মারাত্মক বিরুপ ঘটনা পরিলক্ষিত হয়; হাইপোথার্মিয়া পাওয়া তিনটি (নিউমোনিয়া জনিত কারনে হাসপাতালে পুনঃ ভর্তি, সেপটিক আর্থরাইটিস, সম্ভাব্য ভেনাস থ্রম্বসিস) এবং দুইটি নিয়ন্ত্রণ গ্রুপে (এম আর আই করানোর সময় ডিস্যাচুর‍্যাশন এবং এম আর আই এর স্থানান্তরের সময় শ্বাসনালী র নল সরে যাওয়া)। গবেষণা ইন্টারভেনশনের সাথে কোন মারাত্মক বিরপ ঘটনার সংযোগ পাওয়া যায় নি।

ব্যাখ্যাঃ থেরাপিউটিক হাইপোথার্মিয়া নিম্ন ও মধ্যম আয়ের দেশগুলোতে নবজাতক এনসেফালোপ্যাথি পরবর্তী ১৮ মাসে সম্মিলিত মৃত্যু বা অক্ষমতা কমায় না বরং উল্লেখযোগ্যভাবে শুধুমাত্র মৃত্যু বাড়ায়। তাই, নিম্ন ও মধ্যম আয়ের দেশগুলোতে তৃতীয় স্তরের নিবিড় পরিচর্যা কেন্দ্রের সুবিধা থাকলেও নবজাতক এনসেফালোপ্যাথির চিকিৎসা হিসেবে হাইপোথার্মিয়া চিকিৎসা প্রদান করা উচিত না।

অর্থায়নে- ন্যাশনাল ইনস্টিটিউট অব হেলথ রিসার্চ, গারফিল্ড ওয়েস্টন ফাউন্ডেশন, বিল এন্ড মেলিন্ডা গেটস ফাউন্ডেশন।
