# Supplementary material for: Hypothermia for moderate or severe neonatal encephalopathy in low-income and middle-income countries (HELIX): a randomised controlled trial in India, Sri Lanka, and Bangladesh
Source: Lancet Glob Health. 2021 Aug 3;9(9):e1273–85. doi: 10.1016/S2214-109X(21)00264-3 (PMC8371331; doi:10.1016/S2214-109X(21)00264-3)
Supplement: Supplementary appendix 9 [file mmc9.pdf]

THE LANCET
Global Health

## Supplementary appendix 9

This appendix formed part of the original submission and has been peer reviewed. We post it as supplied by the authors.

Supplement to: Thayyil S, Pant S, Montaldo P, et al. Hypothermia for moderate or severe neonatal encephalopathy in low-income and middle-income countries (HELIX): a randomised controlled trial in India, Sri Lanka, and Bangladesh. *Lancet Glob Health* 2021; published online Aug 3. http://dx.doi.org/10.1016/S2214-109X(21)00264-3.

# Supplementary Appendix

## Index

Table 1. Haematological, biochemical, and clinical support at 24 hours ....... 2
Table 2. Haematological, biochemical, and clinical support at 48 hours ....... 3
Table 3. Haematological, biochemical, and clinical support at 72 hours ....... 4
Table 4. Haematological, biochemical, and clinical support at 96 hours ....... 5
Table 5. Severity of cerebral lesions seen on MRI in cooled and non-cooled infants ....... 6

Figure 1. Change in base excess ....... 7
Figure 2. Proportion of infants with mild or no encephalopathy in the hypothermic (blue) and control (red) groups ....... 8
Figure 3. Age of onset of seizures. ....... 9
Figure 4. Heart rate and blood pressure of the hypothermia and control groups ....... 10
Figure 5. Flow chart of the MR data analysis ....... 11
Figure 6. MRS analysis ....... 12
Figure 7. DTI analysis ....... 17
Figure 8. Effect of hypothermia on mortality at hospital discharge: Subgroup analysis based on place of delivery. ....... 18
Figure 9. Effect of hypothermia on mortality at hospital discharge: Subgroup analysis based on birth weight. ....... 18
Figure 10. Effect of hypothermia on mortality at hospital discharge: Subgroup analysis based on growth restriction 18
Figure 11. Effect of hypothermia on mortality at hospital discharge: Subgroup analysis based on co-existent sepsis. ....... 18
Figure 12. Effect of hypothermia on mortality at hospital discharge: Subgroup analysis based on perinatal sentinel events ....... 18
Figure 13. Effect of hypothermia on mortality at hospital discharge: Subgroup analysis based on socio-demographic index of the region ....... 19
Figure 14. Effect of hypothermia on mortality at hospital discharge: Subgroup analysis based on neonatal mortality rate of the region ....... 19
Figure 15. Effect of hypothermia on death or disability at 18 months: Subgroup analysis based on place of delivery. 20
Figure 16. Effect of hypothermia on death or disability at 18 months: Subgroup analysis based on birth weight. ....... 20
Figure 17. Effect of hypothermia on death or disability at 18 months: Subgroup analysis based on growth restriction ....... 20
Figure 18. Effect of hypothermia on death or disability at 18 months: Subgroup analysis based on co-existent sepsis. 20
Figure 19. Effect of hypothermia on death or disability at 18 months: Subgroup analysis based on perinatal sentinel events ....... 20
Figure 20. Effect of hypothermia on death or disability at 18 months: Subgroup analysis based on socio-demographic index (SDI) of the region. ....... 21
Figure 21. Effect of hypothermia on death or disability at 18 months: Subgroup analysis based on neonatal mortality rate of the region ....... 21
Figure 22. Relation of neonatal mortality (left forest plot) and moderate or severe disability (right forest plot) and therapeutic hypothermia. ....... 22

**Table 1. Haematological, biochemical, and clinical support at 24 hours**

| | Hypothermia group | | Control group | | Difference (95% CI) | p-value |
|---|---|---|---|---|---|---|
| | n | Summary | n | Summary | | |
| Haemoglobin, g per dL | 195 | 16·2 ± 2·5 | 201 | 16·2 ± 2·2 | 0·0 (-0·5, 0·5) | 0·95 |
| Hb <12 per dL | 195 | 8 (4·1%) | 201 | 6 (3·0%) | 1·1% (-2·5%, 4·8%) | 0·55 |
| White blood cells per µL | 193 | 20000 [15100, 26100] | 196 | 21200 [15600, 28000] | -900 (-2610, 1000) | 0·38 |
| WBC <5000 or >15,000 | 193 | 148 (76·7%) | 196 | 160 (81·6%) | -4·9% (-13·0%, 3·1%) | 0·23 |
| Platelets, 105 per µL | 195 | 2·2 ± 0·7 | 200 | 2·3 ± 0·8 | -0·1 (-0·2, 0·1) | 0·50 |
| Platelets <100 000 per µL | 195 | 10 (5·1%) | 200 | 10 (5·0%) | 0·1% (-4·2%, 4·5%) | 0·95 |
| CRP >10 mg per L | 179 | 23 (12·9%) | 178 | 19 (10·7%) | 2·2% (-4·5%, 8·9%) | 0·52 |
| pH | 184 | 7·22 ± 0·15 | 186 | 7·26 ± 0·15 | -0·03 (-0·06, 0·00) | 0·05 |
| pCO2, mm of Hg | 183 | 34·6 ± 17·6 | 183 | 33·0 ± 13·3 | 1·5 (-1·7, 4·7) | 0·35 |
| Base excess (mEq/L) | 174 | -12·5 ± 6·4 | 170 | -11·6 ± 6·1 | -0·9 (-2·2, 0·4) | 0·18 |
| Lowest blood sugar, mg per dL | 182 | 89 ± 38 | 180 | 85 ± 26 | 4 (-2, 11) | 0·21 |
| Blood sugar <40 mg per dl | 182 | 6 (3·3%) | 180 | 3 (1·7%) | 1·6% (-1·6%, 4·8%) | 0·32 |
| Highest blood sugar, mg per dL | 167 | 139 ± 87 | 175 | 115 ± 38 | 25 (10, 39) | <0·001 |
| Blood sugar >125 mg per dL | 167 | 65 (38·9%) | 175 | 54 (30·9%) | 8·1% (-2·0%, 18·1%) | 0·12 |
| Prothrombin time, seconds | 152 | 19·2 [16·5, 24·6] | 157 | 18·5 [15·9, 23·0] | 0·9 (-0·3, 2·0) | 0·17 |
| Activated partial thromboplastin time, seconds | 133 | 45·1 [36·0, 58·0] | 131 | 42·3 [33·5, 58·0] | 2·4 (-1·9, 7·0) | 0·26 |
| International normalised ratio (INR) | 98 | 1·5 [1·2, 2·0] | 111 | 1·4 [1·2, 1·8] | 0·1 (-0·1, 0·2) | 0·27 |
| INR > 1·2 | 98 | 71 (72·5%) | 111 | 84 (75·7%) | -3·2% (-15·1%, 8·7%) | 0·59 |
| Inotropes (any) | 202 | 140 (69·3%) | 206 | 114 (55·3%) | 14·0% (4·7%, 23·3%) | 0·004 |
| Dopamine | 202 | 69 (34·2%) | 206 | 51 (24·8%) | 9·4% (0·6%, 18·2%) | 0·04 |
| Dobutamine | 202 | 124 (61·4%) | 206 | 106 (51·5%) | 9·9% (0·4%, 19·5%) | 0·04 |
| Adrenaline | 202 | 21 (10·4%) | 206 | 14 (6·8%) | 3·6% (-1·8%, 9·0%) | 0·19 |
| Noradrenaline | 202 | 2 (1·0%) | 206 | 1 (0·5%) | 0·5% (-1·1%, 2·2%) | 0·55 |
| Other | 202 | 7 (3·5%) | 206 | 4 (1·9%) | 1·5% (-1·6%, 4·7%) | 0·34 |
| Breathing support | | | | | | |
| None | 202 | 39 (19·3%) | 206 | 59 (28·6%) | -9·3% (-17·5%, -1·1%) | 0·03 |
| Oxygen | 202 | 52 (25·7%) | 206 | 42 (20·4%) | 5·4% (-2·8%, 13·5%) | 0·20 |
| CPAP | 202 | 3 (1·5%) | 206 | 7 (3·4%) | -1·9% (-4·9%, 1·1%) | 0·21 |
| Invasive ventilation | 202 | 108 (53·5%) | 206 | 98 (47·6%) | 5·9% (-3·8%, 15·6%) | 0·23 |
| Sedation (any) | 202 | 65 (32·2%) | 206 | 44 (21·4%) | 10·8% (2·3%, 19·4%) | 0·01 |
| Chloral hydrate | 202 | 10 (5·0%) | 206 | 0 (0·0%) | 5·0% (2·0%, 7·9%) | 0·001 |
| Fentanyl | 202 | 33 (16·3%) | 206 | 25 (12·1%) | 4·2% (-2·6%, 11·0%) | 0·22 |
| Morphine | 202 | 18 (8·9%) | 206 | 12 (5·8%) | 3·1% (-2·0%, 8·2%) | 0·23 |
| Other | 202 | 6 (3·0%) | 206 | 10 (4·9%) | -1·9% (5·6%, 1·9%) | 0·33 |
| Anticonvulsants (any) | 202 | 167 (82·7%) | 206 | 173 (84·0%) | -1·3% (-8·5%, 5·9%) | 0·72 |
| Phenobarbitone | 202 | 165 (81·7%) | 206 | 172 (83·5%) | -1·8% (-9·2%, 5·5%) | 0·63 |
| Phenytoin | 202 | 15 (7·4%) | 260 | 23 (11·2%) | -3·7% (-9·4%, 1·9%) | 0·19 |
| Levetiracetam | 202 | 13 (6·4%) | 206 | 15 (7·3%) | -0·8% (-5·7%, 4·1%) | 0·75 |
| Midazolam | 202 | 6 (3·0%) | 206 | 11 (5·3%) | -2·4% (-6·2%, 1·5%) | 0·23 |
| Others | 202 | 3 (1·5%) | 206 | 5 (2·4%) | -0·9% (-3·6%, 1·7%) | 0·49 |
| Sedation and/or anti-convulsant (any) | 202 | 176 (87·1%) | 206 | 177 (85·9%) | 1·2% (-5·4%, 7·8%) | 0·72 |
| Clinical seizures | 202 | 176 (87·1%) | 206 | 185 (89·8%) | -2·7% (-8·9%, 3·5%) | 0·40 |
| Antibiotics | 201 | 176 (87·6%) | 206 | 181 (87·9%) | -0·3% (-6·7%, 6·1%) | 0·93 |
| Fluids therapy | | | | | | |
| IVF | 202 | 188 (93·1%) | 206 | 180 (87·4%) | 5·7% (0·0%, 11·4%) | 0·05 |
| IVF and NG feeds | 202 | 14 (6·9%) | 206 | 24 (11·7%) | -4·7% (-10·3%, 0·9%) | 0·10 |
| NG feeds | 202 | 0 (0·0%) | 206 | 2 (1·0%) | -1·0% (-2·3%, 0·4%) | 0·16 |
| Cup feeds | 202 | 0 (0·0%) | 260 | 0 (0·0%) | - | - |

Data are mean ± standard deviation, median [inter-quartile range] plus median change (95% confidence intervals), or number (percentage) plus risk difference (95% confidence intervals). The laboratory data are based on the worst value during the first 24 hours after birth and were analysed at standard laboratory conditions without any temperature correction. Seizures are based on the number of babies who had clinical seizures during the first 24 after birth.

WBC: white blood cell count; CRP: C-reactive protein; pCO2: partial pressure of carbon dioxide; CPAP: continuous positive airway pressure; IVF: intravenous fluids; NG: Nasogastric feeds

**Table 2. Haematological, biochemical, and clinical support at 48 hours**

| | Hypothermia group | | Control group | | Difference (95% CI) | p-value |
|---|---|---|---|---|---|---|
| | n | Summary | n | Summary | | |
| Haemoglobin, g per dL | 97 | 15·4 ± 2·6 | 59 | 14·9 ± 2·6 | 0·5 (-0·3, 1·4) | 0·22 |
| Hb <12 per dL | 97 | 8 (8·3%) | 59 | 6 (10·2%) | -1·9% (-11·4%, 7·5%) | 0·68 |
| White blood cells per µL | 95 | 15200 [10700, 19200] | 58 | 18865 [12800, 25000] | -3565 (-6520, -600) | 0·01 |
| WBC <5000 or >15,000 | 95 | 53 (55·8%) | 58 | 43 (74·1%) | -18·3% (-33·4%, -3·3%) | 0·02 |
| Platelets, 105 per µL | 96 | 1·7 ± 0·8 | 59 | 1·7 ± 0·7 | 0·0 (-0·2, 0·3) | 0·89 |
| Platelets <100 000 per µL | 96 | 17 (17·7%) | 59 | 7 (11·9%) | 5·8% (-5·4%, 17·1%) | 0·33 |
| CRP >10 mg per L | 56 | 16 (28·6%) | 39 | 12 (30·8%) | -2·2% (-20·9%, 16·5%) | 0·82 |
| pH | 119 | 7·29 ± 0·12 | 86 | 7·34 ± 0·11 | -0·05 (-0·08, -0·02) | 0·003 |
| pCO2, mm of Hg | 119 | 31·1 ± 10·9 | 86 | 29·5 ± 9·8 | 1·7 (-1·3, 4·6) | 0·26 |
| Base excess (mEq/L) | 114 | -10·4 ± 6·2 | 79 | -8·6 ± 4·7 | -1·8 (-3·4, -0·1) | 0·03 |
| Lowest blood sugar, mg per dL | 162 | 88 ± 28 | 162 | 87 ± 25 | 1 (-5, 7) | 0·75 |
| Blood sugar <40 mg per dl | 162 | 3 (1·9%) | 162 | 0 (0·0%) | 1·9% (-0·2%, 3·9%) | 0·08 |
| Highest blood sugar, mg per dL | 153 | 136 ± 91 | 151 | 111 ± 34 | 25 (10, 41) | 0·002 |
| Blood sugar >125 mg per dL | 153 | 58 (37·9%) | 151 | 33 (21·9%) | 16·1% (5·9%, 26·2%) | 0·002 |
| Prothrombin time, seconds | 48 | 18·4 [15·6, 22·7] | 29 | 15·9 [14·0, 18·5] | 2·3 (0·4, 4·5) | 0·03 |
| Activated partial thromboplastin time, seconds | 37 | 42·0 [38·9, 55·3] | 24 | 42·0 [34·5, 54·0] | 2·1 (-4·9, 11·1) | 0·53 |
| International normalised ratio | 27 | 1·7 [1·3, 2·0] | 14 | 1·3 [1·1, 1·5] | 0·3 (0·1, 0·6) | 0·03 |
| Haemoglobin, g per dL | 27 | 22 (81·5%) | 14 | 8 (57·1%) | 24·3% (-5·4%, 54·1%) | 0·10 |
| Inotropes (any) | 193 | 142 (73·6%) | 199 | 102 (51·3%) | 22·3% (13·0%, 31·6%) | <0·001 |
| Dopamine | 193 | 76 (39·4%) | 199 | 43 (21·6%) | 17·8% (8·8%, 26·7%) | <0·001 |
| Dobutamine | 193 | 132 (68·4%) | 199 | 93 (46·7%) | 21·7% (12·1%, 31·2%) | <0·001 |
| Adrenaline | 193 | 27 (14·0%) | 199 | 21 (10·6%) | 3·4% (-3·1%, 9·9%) | 0·30 |
| Noradrenaline | 193 | 5 (2·6%) | 199 | 3 (1·5%) | 1·1% (-1·7%, 3·9%) | 0·45 |
| Other | 193 | 4 (2·1%) | 199 | 5 (2·5%) | -0·4% (-3·4%, 2·5%) | 0·77 |
| Breathing support | | | | | | |
| None | 193 | 59 (30·6%) | 199 | 83 (41·7%) | -11·1% (-20·6%, -1·7%) | 0·02 |
| Oxygen | 193 | 34 (16·8%) | 199 | 25 (12·1%) | 4·7% (-2·1%, 11·5%) | 0·18 |
| CPAP | 193 | 3 (1·5%) | 199 | 7 (3·4%) | -1·9% (-4·9%, 1·1%) | 0·21 |
| Invasive ventilation | 193 | 97 (48·0%) | 199 | 85 (41·3%) | 6·8% (-2·9%, 16·4%) | 0·17 |
| Sedation (any) | 193 | 69 (35·8%) | 199 | 46 (23·1%) | 12·6% (3·7%, 21·6%) | 0·006 |
| Chloral hydrate | 193 | 10 (5·2%) | 199 | 0 (0·0%) | 5·2% (2·1%, 8·3%) | 0·001 |
| Fentanyl | 193 | 35 (18·1%) | 199 | 25 (12·6%) | 5·6% (-1·6%, 12·7%) | 0·13 |
| Morphine | 193 | 15 (7·8%) | 199 | 9 (4·5%) | 3·2% (-1·5%, 8·0%) | 0·18 |
| Other | 193 | 10 (5·2%) | 199 | 14 (7·0%) | -1·9% (-6·6%, 2·9%) | 0·44 |
| Anticonvulsants (any) | 193 | 155 (80·3%) | 199 | 169 (84·9%) | -4·6% (-12·1%, 2·9%) | 0·23 |
| Phenobarbitone | 193 | 149 (77·2%) | 199 | 166 (83·4%) | -6·2% (-14·1%, 1·6%) | 0·12 |
| Phenytoin | 193 | 18 (9·3%) | 199 | 39 (18·1%) | -8·8% (-15·5%, -2·0%) | 0·01 |
| Levetiracetam | 193 | 20 (10·4%) | 199 | 18 (9·1%) | 1·3% (-4·5%, 7·2%) | 0·66 |
| Midazolam | 193 | 8 (4·2%) | 199 | 8 (4·0%) | 0·1% (-3·8%, 4·0%) | 0·95 |
| Others | 193 | 1 (0·5%) | 199 | 4 (2·0%) | -1·5% (-3·7%, 0·7%) | 0·19 |
| Sedation and/or anti-convulsant (any) | 193 | 161 (83·4%) | 206 | 175 (87·9%) | -4·5% (-11·4%, 2·4%) | 0·20 |
| Clinical seizures | 192 | 34 (17·7%) | 199 | 49 (24·6%) | -6·9% (-15·0%, 1·1%) | 0·09 |
| Antibiotics | 191 | 176 (92·2%) | 176 | 176 (88·4%) | 3·7% (-2·2%, 9·6%) | 0·22 |
| Fluids therapy | | | | | | |
| IVF | 193 | 152 (78·8%) | 199 | 138 (69·4%) | 9·4% (0·8%, 18·0%) | 0·03 |
| IVF and NG feeds | 193 | 41 (21·2%) | 199 | 54 (27·1%) | -5·9% (-14·3%, 2·6%) | 0·17 |
| NG feeds | 193 | 0 (0·0%) | 199 | 7 (3·5%) | -3·5% (-6·1%, -1·0%) | 0·009 |
| Cup feeds | 193 | 0 (0·0%) | 199 | 1 (0·5%) | -0·5% (-1·5%, 0·5%) | 0·32 |

Data are mean (standard deviation), median [inter-quartile range] plus median change (95% confidence intervals), or number (percentage) plus risk difference (95% confidence intervals). The laboratory data are based on the worst value between 24 to 48 hours after birth and were analysed at standard laboratory conditions without any temperature correction. Seizures are based on the number of babies who had clinical seizures between 24 to 48 hours after birth.

WBC: white blood cell count; CRP: C-reactive protein; pCO2: partial pressure of carbon dioxide; CPAP: continuous positive airway pressure; IVF: intravenous fluids; NG: Nasogastric feeds

**Table 3. Haematological, biochemical, and clinical support at 72 hours**

| | Hypothermia group | | Control group | | Difference (95% CI) | p-value |
|---|---|---|---|---|---|---|
| | n | Summary | n | Summary | | |
| Haemoglobin, g per dL | 79 | 14·5 ± 2·7 | 62 | 14·7 ± 2·1 | -0·2 (-1·1, 0·6) | 0·61 |
| Hb <12 per dL | 79 | 13 (16·5%) | 62 | 4 (6·5%) | 10·0% (-0·2%, 20·2%) | 0·07 |
| White blood cells per µL | 79 | 10500 [7860, 15470] | 62 | 12450 [9300, 16300] | -2000 (-3800, -200) | 0·03 |
| WBC <5000 or >15,000 | 79 | 30 (38·0%) | 62 | 24 (38·7%) | -0·7% (-16·9%, 15·4%) | 0·93 |
| Platelets, 105 per µL | 79 | 1·4 ± 0·8 | 62 | 1·7 ± 0·7 | -0·3 (-0·5, 0·0) | 0·03 |
| Platelets <100 000 per µL | 79 | 18 (22·8%) | 62 | 10 (16·1%) | 6·7% (-6·4%, 19·7%) | 0·33 |
| CRP >10 mg per L | 38 | 17 (44·7%) | 37 | 13 (35·1%) | 9·6% (-12·5%, 31·7%) | 0·40 |
| pH | 89 | 7·32 ± 0·09 | 68 | 7·36 ± 0·09 | -0·04 (-0·07, -0·01) | 0·005 |
| pCO2, mm of Hg | 89 | 31·8 ± 10·8 | 68 | 33·3 ± 10·3 | -1·5 (-4·9, 1·9) | 0·37 |
| Base excess (mEq/L) | 84 | -8·7 ± 5·5 | 65 | -6·2 ± 3·8 | -2·5 (-4·1, -0·9) | 0·002 |
| Lowest blood sugar, mg per dL | 152 | 83 ± 22 | 146 | 84 ± 21 | -1 (-6, 4) | 0·78 |
| Blood sugar <40 mg per dl | 152 | 2 (1·3%) | 146 | 1 (0·7%) | 0·6% (-1·6%, 2·9%) | 0·59 |
| Highest blood sugar, mg per dL | 138 | 115 ± 45 | 135 | 102 ± 23 | 13 (4, 22) | 0·003 |
| Blood sugar >125 mg per dL | 138 | 31 (22·5%) | 135 | 15 (11·1%) | 11·4% (2·6%, 20·1%) | 0·01 |
| Prothrombin time, seconds | 34 | 17·5 [15·0, 23·2] | 21 | 15·0 [13·5, 16·6] | 2·5 (0·6, 4·9) | 0·01 |
| Activated partial thromboplastin time, seconds | 31 | 42·9 [38·8, 49·3] | 20 | 41·6 [33·1, 44·4] | 3·4 (-2·0, 9·2) | 0·23 |
| International normalised ratio | 15 | 1·4 [1·2, 2·3] | 8 | 1·4 [1·2, 1·5] | 0·2 (-0·1, 0·8) | 0·37 |
| Haemoglobin, g per dL | 15 | 11 (73·3%) | 8 | 6 (75·0%) | -1·7% (-39·1%, 35·8%) | 0·93 |
| Inotropes (any) | 182 | 122 (67·0%) | 189 | 72 (38·1%) | 28·9% (19·2%, 38·7%) | <0·001 |
| Dopamine | 182 | 59 (32·4%) | 189 | 33 (17·5%) | 15·0% (6·3%, 23·3%) | <0·001 |
| Dobutamine | 182 | 111 (61·0%) | 189 | 67 (35·5%) | 25·5% (15·7%, 35·4%) | <0·001 |
| Adrenaline | 182 | 28 (15·4%) | 189 | 12 (6·4%) | 9·0% (2·7%, 15·3%) | 0·005 |
| Noradrenaline | 182 | 7 (3·9%) | 189 | 2 (1·1%) | 2·8% (-0·4%, 5·9%) | 0·08 |
| Other | 182 | 6 (3·3%) | 189 | 6 (3·2%) | 0·1% (-3·5%, 3·7%) | 0·95 |
| Breathing support | | | | | | |
| None | 182 | 72 (39·6%) | 189 | 92 (48·7%) | -9·1% (-19·2%, 0·9%) | 0·08 |
| Oxygen | 182 | 22 (12·1%) | 189 | 25 (13·2%) | -1·1% (-7·9%, 5·6%) | 0·74 |
| CPAP | 182 | 8 (4·4%) | 189 | 10 (5·3%) | -0·9% (-5·3%, 3·5%) | 0·69 |
| Invasive ventilation | 182 | 80 (44·0%) | 189 | 62 (32·8%) | 11·2% (1·3%, 21·0%) | 0·03 |
| Sedation (any) | 182 | 59 (32·4%) | 189 | 27 (14·3%) | 18·1% (9·7%, 26·6%) | <0·001 |
| Chloral hydrate | 182 | 12 (6·6%) | 189 | 0 (0·0%) | 6·6% (3·0%, 10·2%) | <0·001 |
| Fentanyl | 182 | 28 (15·4%) | 189 | 16 (8·5%) | 6·9% (0·3%, 13·5%) | 0·04 |
| Morphine | 182 | 11 (6·0%) | 189 | 4 (2·1%) | 3·9% (-0·1%, 8·0%) | 0·06 |
| Other | 182 | 9 (5·0%) | 189 | 10 (5·3%) | -0·3% (-4·8%, 4·1%) | 0·88 |
| Anticonvulsants (any) | 182 | 149 (81·9%) | 189 | 152 (80·4%) | 1·4% (-6·5%, 9·4%) | 0·72 |
| Phenobarbitone | 182 | 145 (79·7%) | 189 | 149 (78·8%) | 0·8% (-7·4%, 9·1%) | 0·84 |
| Phenytoin | 182 | 19 (10·4%) | 189 | 27 (14·3%) | -3·8% (-10·5%, 2·8%) | 0·26 |
| Levetiracetam | 182 | 14 (7·7%) | 189 | 19 (10·1%) | -2·4% (-8·1%, 3·4%) | 0·42 |
| Midazolam | 182 | 5 (2·8%) | 189 | 6 (3·2%) | -0·4% (-3·9%, 3·0%) | 0·81 |
| Others | 182 | 0 (0·0%) | 189 | 1 (0·5%) | -0·5% (-1·6%, 0·5%) | 0·33 |
| Sedation and/or anti-convulsant (any) | 182 | 154 (84·6%) | 189 | 157 (83·1%) | 1·5% (-5·9%, 9·0%) | 0·69 |
| Clinical seizures | 181 | 11 (6·1%) | 188 | 12 (6·4%) | -0·3% (-5·2%, 4·6%) | 0·90 |
| Antibiotics | 181 | 171 (94·5%) | 189 | 164 (86·8%) | 7·7% (1·8%, 13·5%) | 0·01 |
| Fluids therapy | | | | | | |
| IVF | 182 | 129 (70·9%) | 188 | 98 (52·1%) | 18·8% (9·0%, 28·5%) | <0·001 |
| IVF and NG feeds | 182 | 53 (29·1%) | 188 | 72 (38·3%) | -9·2% (-18·7%, 0·4%) | 0·06 |
| NG feeds | 182 | 0 (0·0%) | 188 | 14 (7·5%) | -7·5% (-11·2%, -3·7%) | <0·001 |
| Cup feeds | 182 | 0 (0·0%) | 188 | 6 (2·9%) | -2·9% (-5·2%, -0·6%) | 0·01 |

Data are mean (standard deviation), median [inter-quartile range] plus median change (95% confidence intervals), or number (percentage) plus risk difference (95% confidence intervals). The laboratory data are based on the worst value between 48 to 72 hours after birth and were analysed at standard laboratory conditions without any temperature correction. Seizures are based on the number of babies who had clinical seizures between 48 to 72 hours after birth.

WBC: white blood cell count; CRP: C-reactive protein; pCO2: partial pressure of carbon dioxide; CPAP: continuous positive airway pressure; IVF: intravenous fluids; NG: Nasogastric feeds

**Table 4. Haematological, biochemical, and clinical support at 96 hours**

| | Hypothermia group | | Control group | | Difference (95% CI) | p-value |
|---|---|---|---|---|---|---|
| | n | Summary | n | Summary | | |
| Haemoglobin, g per dL | 83 | 14·3 ± 2·6 | 54 | 14·0 ± 2·3 | 0·3 (-0·6, 1·2) | 0·48 |
| Hb <12 per dL | 83 | 14 (16·9%) | 54 | 7 (13·0%) | 3·9% (-8·1%, 16·0%) | 0·54 |
| White blood cells per µL | 81 | 9500 [6500, 12500] | 53 | 11500 [7300, 14100] | -1500 (-3210, 200) | 0·08 |
| WBC <5000 or >15,000 | 81 | 19 (23·5%) | 53 | 13 (24·5%) | -1·1% (-15·9%, 13·7%) | 0·89 |
| Platelets, 105 per µL | 81 | 1·4 ± 0·8 | 55 | 1·6 ± 1·1 | -0·2 (-0·5, 0·1) | 0·20 |
| Platelets <100 000 per µL | 81 | 25 (30·9%) | 55 | 17 (30·9%) | 0·0% (-15·9%, 15·8%) | 0·99 |
| CRP >10 mg per L | 43 | 19 (44·2%) | 40 | 19 (47·5%) | -3·3% (-24·8%, 18·1%) | 0·76 |
| pH | 77 | 7·32 ± 0·09 | 54 | 7·34 ± 0·09 | -0·02 (-0·05, 0·01) | 0·21 |
| pCO2, mm of Hg | 77 | 36·4 ± 13·1 | 55 | 34·6 ± 10·2 | 1·7 (-2·5, 5·9) | 0·42 |
| Base excess (mEq/L) | 71 | -6·2 ± 6·1 | 50 | -5·3 ± 5·3 | -1·0 (-3·1, 1·2) | 0·37 |
| Lowest blood sugar, mg per dL | 131 | 83 ± 20 | 125 | 83 ± 21 | -1 (-6, 5) | 0·83 |
| Blood sugar <40 mg per dl | 131 | 1 (0·8%) | 125 | 1 (0·8%) | 0·0% (-2·2%, 2·1%) | 0·97 |
| Highest blood sugar, mg per dL | 117 | 105 ± 27 | 111 | 102 ± 21 | 3 (-4, 9) | 0·44 |
| Blood sugar >125 mg per dL | 117 | 18 (15·4%) | 111 | 11 (9·9%) | 5·5% (-3·1%, 14·1%) | 0·21 |
| Prothrombin time, seconds | 30 | 16·9 [14·4, 20·9] | 17 | 14·0 [12·6, 16·3] | 2·7 (0·4, 5·1) | 0·03 |
| Activated partial thromboplastin time, seconds | 24 | 57·6 [43·9, 87·7] | 18 | 36·8 [30·4, 44·0] | 18·2 (7·0, 32·9) | 0·004 |
| International normalised ratio | 22 | 1·6 [1·3, 2·0] | 11 | 1·1 [1·1, 1·3] | 0·3 (0·0, 0·6) | 0·04 |
| Haemoglobin, g per dL | 22 | 17 (77·3%) | 11 | 4 (36·4%) | 40·9% (7·5%, 74·3%) | 0·02 |
| Inotropes (any) | 170 | 90 (52·9%) | 184 | 50 (27·2%) | 25·8% (15·9%, 35·6%) | <0·001 |
| Dopamine | 170 | 49 (28·8%) | 184 | 27 (14·7%) | 14·1% (5·6%, 22·3%) | 0·001 |
| Dobutamine | 170 | 78 (45·9%) | 184 | 45 (24·5%) | 21·4% (11·7%, 31·2%) | <0·001 |
| Adrenaline | 170 | 18 (10·6%) | 184 | 13 (7·1%) | 3·5% (-2·4%, 9·4%) | 0·24 |
| Noradrenaline | 170 | 2 (1·2%) | 184 | 2 (1·1%) | 0·1% (-2·1%, 2·3%) | 0·94 |
| Other | 170 | 6 (3·5%) | 184 | 5 (2·7%) | 0·8% (-2·8%, 4·4%) | 0·66 |
| Breathing support | | | | | | |
| None | 170 | 74 (43·5%) | 183 | 103 (56·3%) | -12·8% (-23·1%, -2·4%) | 0·02 |
| Oxygen | 170 | 19 (11·2%) | 183 | 18 (9·8%) | 1·3% (-5·1%, 7·7%) | 0·68 |
| CPAP | 170 | 14 (8·2%) | 183 | 9 (4·9%) | 3·3% (-1·9%, 8·5%) | 0·21 |
| Invasive ventilation | 170 | 63 (37·1%) | 183 | 53 (29·0%) | 8·1% (-1·7%, 17·9%) | 0·11 |
| Sedation (any) | 170 | 32 (18·8%) | 184 | 24 (13·0%) | 5·8% (-1·8%, 13·4%) | 0·14 |
| Chloral hydrate | 170 | 4 (2·4%) | 184 | 1 (0·5%) | 1·8% (-0·7%, 4·3%) | 0·15 |
| Fentanyl | 170 | 23 (13·5%) | 184 | 16 (8·7%) | 4·8% (-1·7%, 11·4%) | 0·15 |
| Morphine | 170 | 3 (1·2%) | 184 | 6 (0·5%) | 0·6% (-1·3%, 2·6%) | 0·52 |
| Other | 170 | 10 (1·8%) | 184 | 14 (3·3%) | -1·5% (-4·7%, 1·7%) | 0·37 |
| Anticonvulsants (any) | 170 | 131 (77·1%) | 184 | 145 (78·8%) | -1·7% (-10·4%, 6·9%) | 0·69 |
| Phenobarbitone | 170 | 128 (75·3%) | 184 | 140 (76·1%) | -0·8% (-9·7%, 8·2%) | 0·86 |
| Phenytoin | 170 | 16 (9·4%) | 184 | 23 (12·5%) | -3·1% (-9·6%, 3·4%) | 0·35 |
| Levetiracetam | 170 | 18 (10·6%) | 184 | 17 (9·2%) | 1·3% (-4·9%, 7·6%) | 0·67 |
| Midazolam | 170 | 1 (0·6%) | 184 | 4 (2·2%) | -1·6% (-4·0%, 0·8%) | 0·21 |
| Others | 170 | 1 (0·6%) | 184 | 2 (1·1%) | -0·5% (-2·4%, 1·4%) | 0·61 |
| Sedation and/or anti-convulsant (any) | 170 | 138 (81·2%) | 184 | 149 (81·0%) | 0·2% (-8·0%, 8·4%) | 0·96 |
| Clinical seizures | 170 | 4 (2·4%) | 184 | 3 (1·6%) | 0·7% (-2·2%, 3·6%) | 0·63 |
| Antibiotics | 169 | 153 (90·5%) | 155 | 155 (84·2%) | 6·3% (-0·5%, 13·2%) | 0·08 |
| Fluids therapy | | | | | | |
| IVF | 170 | 87 (51·2%) | 184 | 71 (38·6%) | 12·6% (2·3%, 22·9%) | 0·02 |
| IVF and NG feeds | 170 | 75 (44·1%) | 184 | 77 (41·9%) | 2·3% (-8·1%, 12·6%) | 0·67 |
| NG feeds | 170 | 5 (2·9%) | 184 | 29 (15·8%) | -12·8% (-18·7%, -7·0%) | <0·001 |
| Cup feeds | 170 | 3 (1·8%) | 184 | 9 (4·9%) | -3·1% (-6·8%, 0·6%) | 0·10 |

Data are mean (standard deviation), median [inter-quartile range] plus median change (95% confidence intervals), or number (percentage) plus risk difference (95% confidence intervals). The laboratory data are based on the worst value between 72 to 96 hours after birth and were analysed at standard laboratory conditions without any temperature correction. Seizures are based on the number of babies who had clinical seizures between 72 to 96 hours after birth.

WBC: white blood cell count; CRP: C-reactive protein; pCO2: partial pressure of carbon dioxide; CPAP: continuous positive airway pressure; IVF: intravenous fluids; NG: Nasogastric feeds

**Table 5. Severity of cerebral lesions seen on MRI in cooled and non-cooled infants**

| | **Hypothermia group (n=122)** | **Control group (n=145)** | **Odds Ratio (95% CI)** | **p** |
|---|---|---|---|---|
| Basal ganglia and thalami | | | | |
| 0 | 96 (78·7%) | 108(74·5%) | 0·80 (0·45-1·41) | 0·44 |
| 1 | 7 (5·8%) | 10 (6·9%) | | |
| 2 | 10 (8·1%) | 15(10·4%) | | |
| 3 | 9 (7·4%) | 12(8·2%) | | |
| Posterior limb of internal capsule | | | | |
| Normal | 101 (82·8%) | 112 (77·2 %) | 0·70 (0·38-1·28) | 0·25 |
| Equivocal | 5 (4·1%) | 6 (4·1%) | | |
| Abnormal | 16 (13·1%) | 27 (18·6%) | | |
| White matter | | | | |
| Normal | 22 (18·0%) | 33(22·8%) | 1·00 (0·64-1·57) | 0·98 |
| 1 | 30 (24·6%) | 28 (19·3%) | | |
| 2 | 58(47·5%) | 66 (45·5%) | | |
| 3 | 12 (9·8%) | 18 (12·4%) | | |
| Cortex | | | | |
| 0 | 91 (74·6%) | 99 (68·3%) | 0·74 (0·43-1·25) | 0·26 |
| 1 | 19 (15·6%) | 26 (17·9%) | | |
| 2 | 3(2·5%) | 9 (6·2%) | | |
| 3 | 9 (7·4%) | 11 (7·6%) | | |
| Basal ganglia/thalami (>0) AND white matter (>0) OR cortical (>0) injury | 23 (18·9%) | 33 (22·8%) | 0·79 (0·43-1·43) | 0·44 |

Data are number (%) or Odds ratio (OR) (95% Confidence interval: CI).
*Odds ratio for MRI abnormalities score in cooled relative to non-cooled infants from ordinal logistic regression analysis.
Basal ganglia and thalamic score: 0=normal, 1=mild (focal abnormal signal intensity), 2=moderate (multifocal abnormal signal intensity), 3=severe (widespread abnormal signal intensity).
White matter score: 0=normal, 1=mild (exaggerated long T1 and long T2 in periventricular white matter only), 2=moderate (long T1 and long T2 extending out to subcortical white matter and /or focal punctate lesions or focal area of infarction), 3=severe widespread abnormalities including overt infarction, haemorrhage, and long T1 and long T2.
Cortical involvement was scored as the presence of abnormal signal intensity, usually decreased T1 or cortical highlighting. 0=normal, 1=mild (1–2 sites involved), 2=moderate (3 sites involved), 3=severe (more than 3 sites involved).

**Figure 1. Change in base excess**

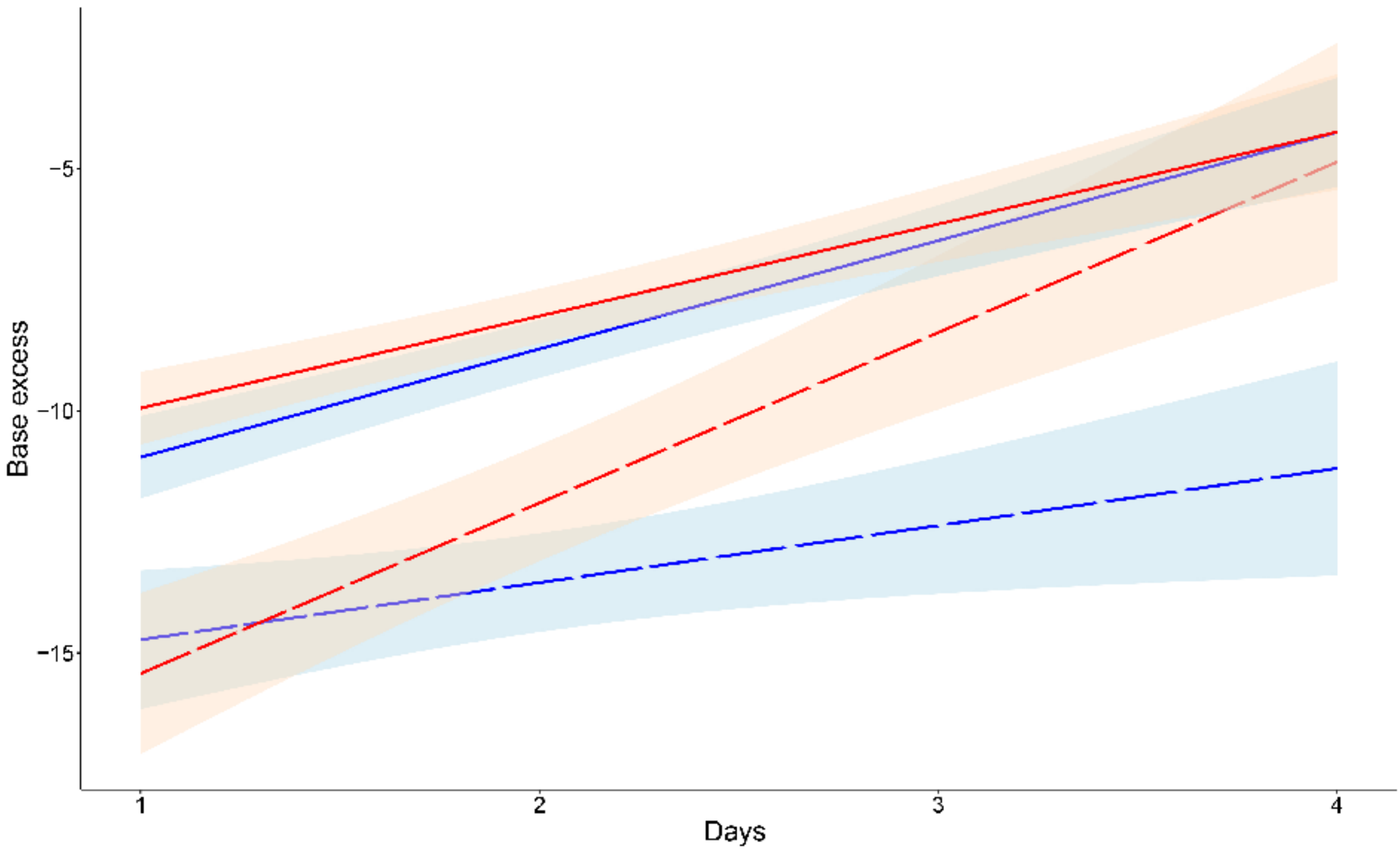


Mean (95% confidence interval) base excess (mEq/L) in the hypothermia (blue line) and control group (red line). The worst base excess for each 24 hour period was fitted using a linear regression model. Continuous line indicates survivors and broken line indicates non-survivors.

**Figure 2. Proportion of infants with mild or no encephalopathy in the hypothermic (blue) and control (red) groups.**

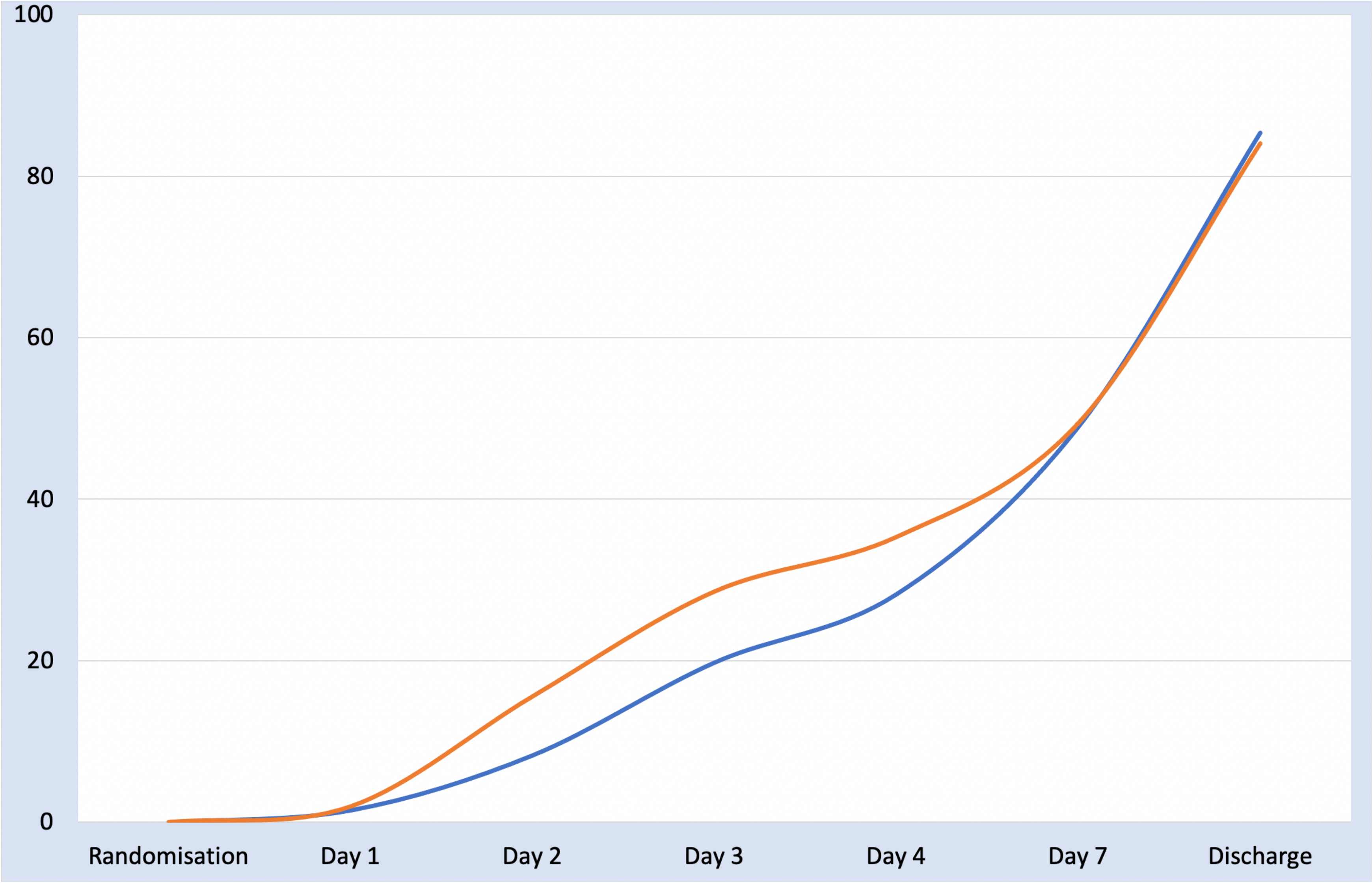

**Figure 3. Age of onset of seizures.**

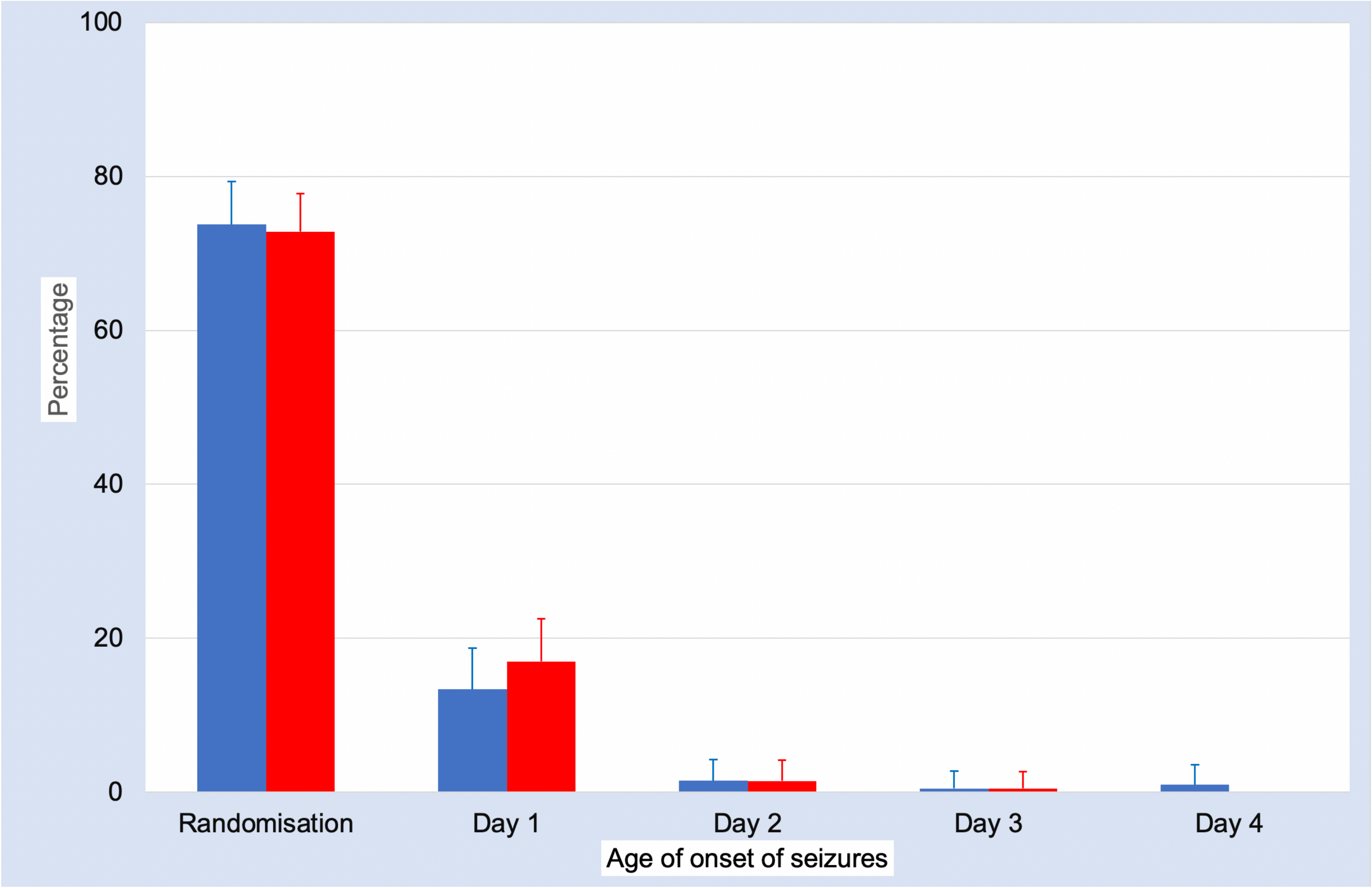


Error bars indicate 95% confidence intervals in the hypothermic (blue) and control (red) group.

**Figure 4. Heart rate and blood pressure of the hypothermia and control groups**

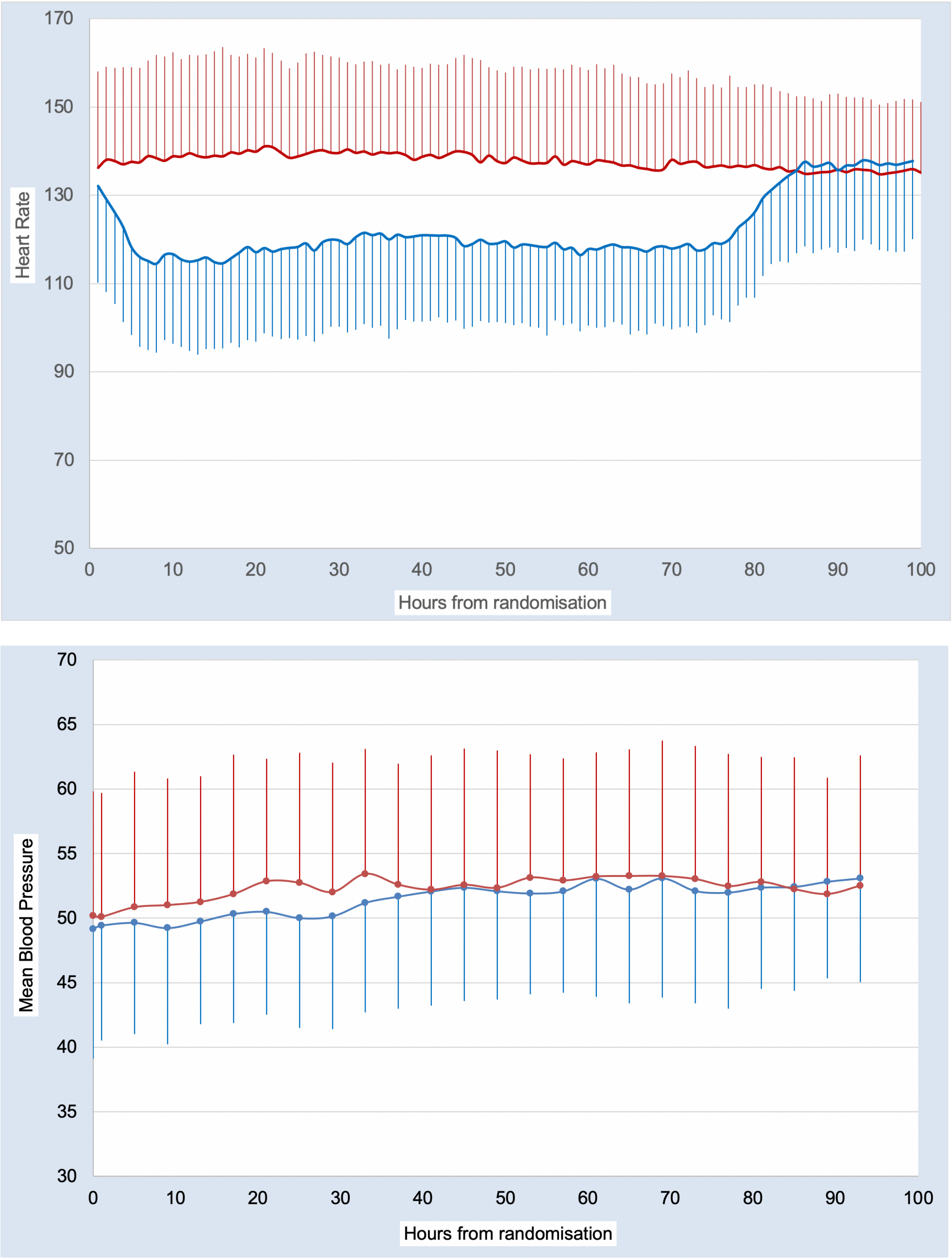


Mean (standard deviation) of hourly heart rate (middle panel) and 4 hourly non-invasive blood pressure (bottom panel) in the hypothermia group (blue) and control group (red).

**Figure 5. Flow chart of the MR data analysis**

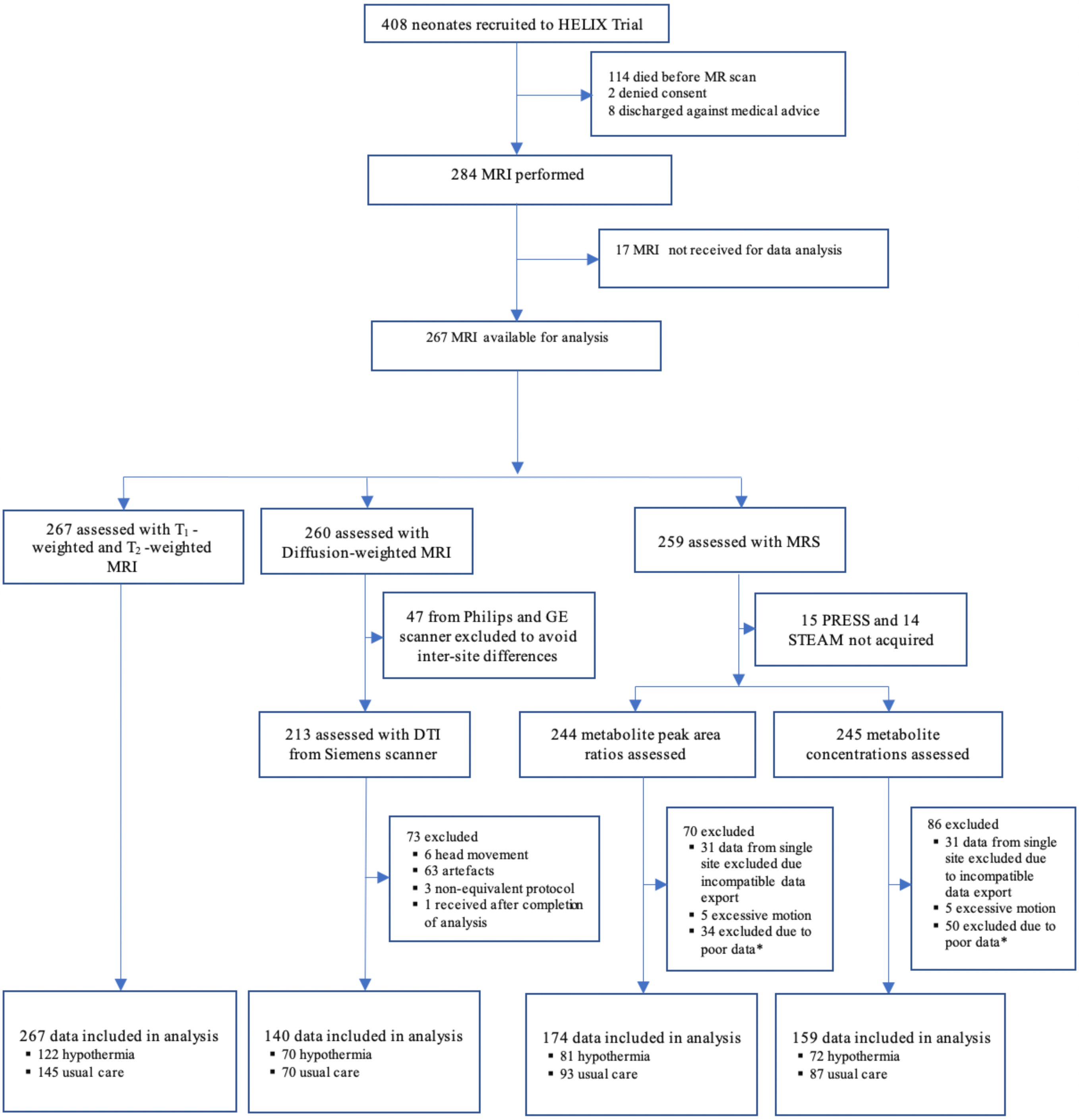


*Rejection criteria for MR spectroscopy included data artefacts, wild baseline, bad shimming, and poor eddy current correction.

**Figure 6. MRS analysis**

**In-vivo MR Spectroscopy Data Acquisition Protocol:**

**Table S1: PRESS Protocol: Peak Area Ratios (PARs) estimation**

| Step | TE (ms) | TR (ms) | Water suppression | Phase cycling | Sub-spectra | Dummy scans | Centre frequency (ppm) | Acquisition time (min:sec) |
|---|---|---|---|---|---|---|---|---|
| 1 | 288 | 2290 | On | 8 | 16 | 1 | 2·01 | 6:43 |
| 2 | 288 | 2290 | Off | 8 | 1 | 1 | 4·67 | 0:21 |

**Table S2: STEAM Protocol: tNAA concentration estimation**

| Step | TE (ms) | TM (ms) | TR (ms) | Water suppression | Phase cycling | Sub-spectra | Dummy scans | Centre frequency (ppm) | Acquisition time (min:sec) |
|---|---|---|---|---|---|---|---|---|---|
| 1 | 20 | 20 | 1500 | On | 4 | 24 | 1 | 2·01 | 3:00 |
| 2 | 20 | 20 | 1500 | Off | 4 | 1 | 1 | 4·67 | 0:12 |
| 3 | 20 | 20 | 3500 | On | 4 | 20 | 1 | 2·01 | 5:50 |
| 4 | 20 | 20 | 3500 | Off | 4 | 1 | 1 | 4·67 | 0:18 |
| OR | | | | | | | | | |
| 3 | 20 | 20 | 5000 | On | 4 | 20 | 1 | 2·01 | 8:20 |
| 4 | 20 | 20 | 5000 | Off | 4 | 1 | 1 | 4·67 | 0:40 |
| 5 | 20 | 20 | 9030 | Off | 1 | 1 | 1 | 4·67 | 0:18 |
| 6 | 40 | 20 | 9040 | Off | 1 | 1 | 1 | 4·67 | 0:18 |
| 7 | 80 | 20 | 9060 | Off | 1 | 1 | 1 | 4·67 | 0:18 |
| 8 | 140 | 20 | 9090 | Off | 1 | 1 | 1 | 4·67 | 0:18 |
| 9 | 220 | 20 | 9130 | Off | 1 | 1 | 1 | 4·67 | 0:18 |
| 10 | 300 | 20 | 9170 | Off | 1 | 1 | 1 | 4·67 | 0:18 |

**Quality assurance:**

The inter-site variation in MRS measurements was quantified across all scanners using a spherical phantom containing a buffered solution of 10mM N-acetylaspartate (NAA) and 10mM lactate (Lac). *In vivo* quality assurance included the visual assessment of motion from imaging before and after MRS; protocol adherence; manual rejection of motion corrupted sub-spectra; automated spectral corrections for both frequency and phase; and the rejection of spectra with linewidths outside the normal distribution of the full dataset.

**Representative *in-vivo* spectra:**

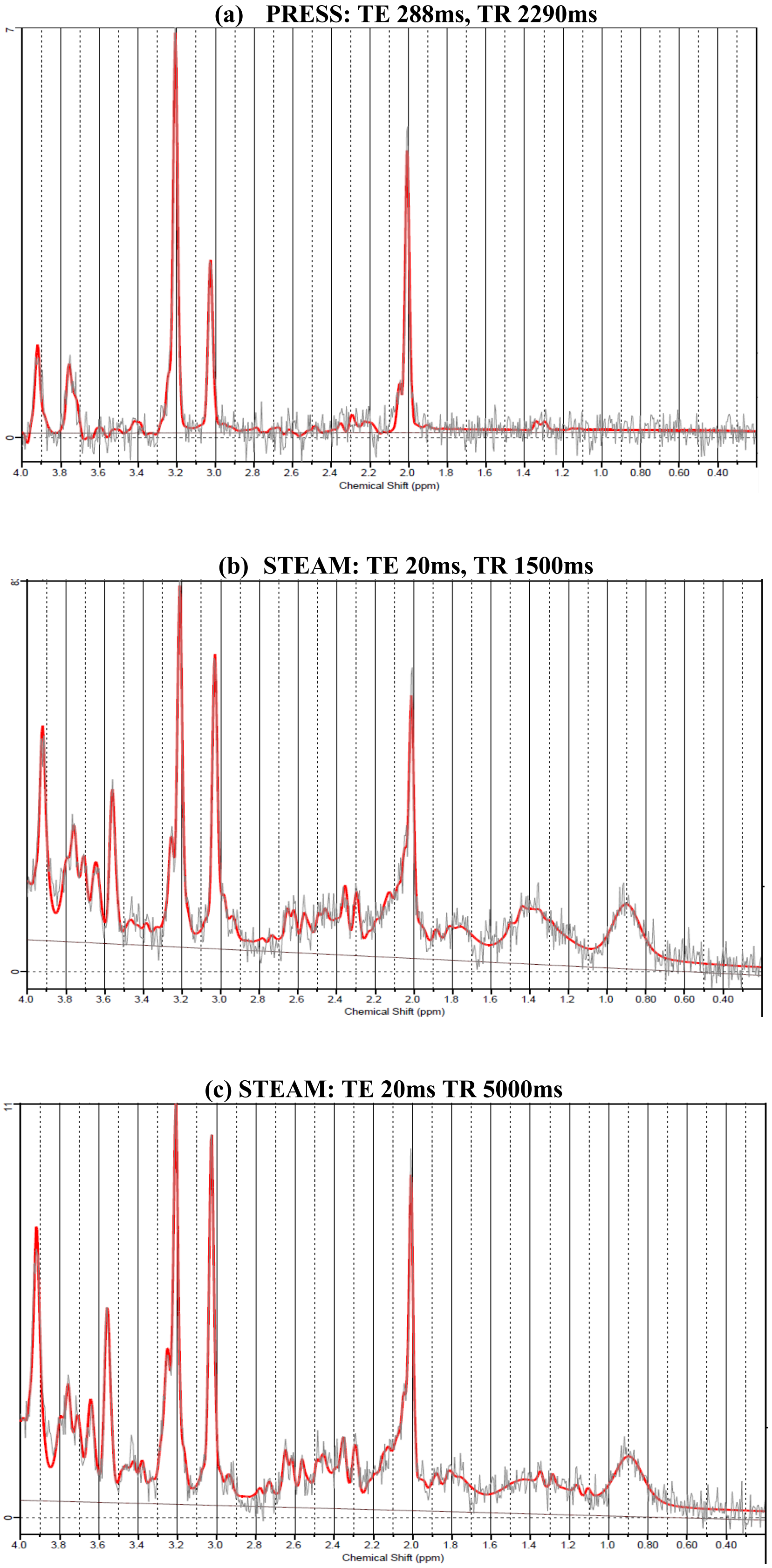


**Figure S1: Spectra collected according to the protocol, with the LCModel fit overlaid (in red).** (a) The PRESS TE 288ms, TR 2290ms acquisition protocol for peak area ration (Lac/NAA, NAA/Cr, NAA/Cho) incorporated the $T_1$ or $T_2$ relaxation effects in the observed metabolites. (b) STEAM TE 20ms TR 1500ms (c) STEAM TE 20ms TR 5000ms data acquired on the same participant for the tNAA concentration estimation.

**Analysis:**

MR spectroscopy data was first analysed in LCModel (Provencher, 2001) with the basis set, optimised for the timings of the Philips implementation and with ideal RF pulses. This was then repeated in LCModel with the STEAM protocol, generating a basis set with the same 1Hz Gaussian lineshape in VeSPA-Simulate (Soher et al., 2011), and including the same list of metabolites (with NAA, NAAG, Cho, Cr and PCr methyl peaks). From the STEAM series, the same relaxivity corrections were applied to the metabolite signals, and water signals.

All water suppressed spectra were analysed using LCModel (v6·3-1J), with basis sets simulated using VeSPA (v0·9·11) with ideal RF pulses according to the PRESS (TE = 288 ms) and STEAM (TE = 20 ms) sequence timings employed by each vendor for each acquisition (personal communication). The following metabolites were included in the simulations and analyses: acetate (Act), alanine (Ala), ascorbate (Asc), betaine (Bet), aspartate (Asp), choline (Cho), phosphocholine (PCh), glycerophosphocholine (GPC), creatine (Cr), phosphocreatine (PCr), gamma-aminobutyric acid (GABA), glucose (Glc), glutamate (Glu), glutamine (Gln), glutathione (GSH), glycine (Glyc), lactate (Lac), myo-inositol (mIns), N-acetylaspartate (NAA), N-acetylaspartyl glutamate (NAAG), phosphoethanolamine (PE), propylene glycol (PGC), scyllo-inositol (Scyllo), taurine (Tau), and threonine (Thr).

Specific LCModel control parameters were, for PRESS (TE=288 ms):

NSIMUL=0
NCOMBI=17
CHCOMB(17)='Lac+Thr'
PPMST=4·0(default)
PPMEND=0·2 (default)

And for STEAM (TE=20 ms):

NSIMUL=11
NCOMBI=17
CHCOMB(17)='Lac+Thr'
PPMST=4·0 (default)
PPMEND=0·2 (default)

The methyl peaks of NAA, NAAG, choline (Cho), phosphocreatine (PCr), and creatine (Cr) were separated from other groups in the basis spectra to allow quantification of individual relaxation rates. NAA+NAAG methyl peaks at ~2·0ppm were combined and referred to as 'NAA', and PCr+Cr methyl peaks at ~3·0ppm were combined and referred to as 'Cr' due to strong covariance. Lac+Thr were combined and referred to as 'Lac' due to strong covariance. A single peak was used to fit the choline signal at ~3·2ppm and referred to as 'Cho'. Water unsuppressed signals were quantified using HLSVD.

NAA/Cho, NAA/Cr and Lac/NAA were all derived from the LCModel fitted results of first water suppressed PRESS acquisition only (TR/TE=2290ms/288ms) (Figure S1a). [tNAA] was calculated from the fitted NAA methyl singlets through two STEAM experiments (TE=20 ms; TM= 20ms, TR=1500; 5000 (or 3500 ms) acquired for T1 and T2 corrections) (Figure S1 b and c), comparing the relaxation corrected NAA signal to the relaxation corrected unsuppressed water signal. This process is outlined in the following Figure S2:

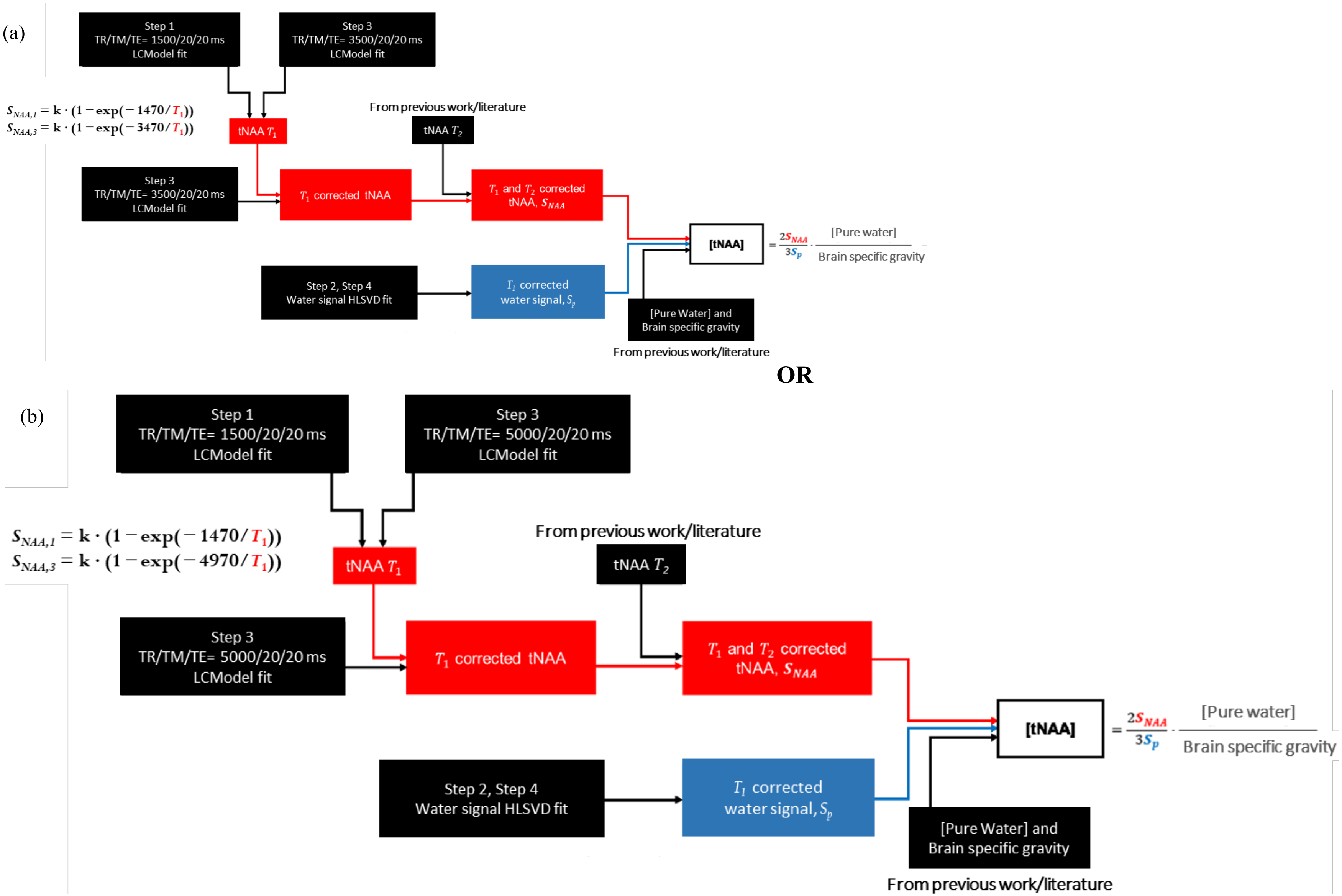


**Figure S2: Calculation steps to derive thalamic NAA concentration, [NAA], from the data collected in the various steps of the STEAM protocol** (a) TE/TM/TR = 20/20/3500 ms, (b) TE/TM/TR = 20/20/5000 ms. Black boxes show the input data, either obtained from the LCModel/HLSVD fits or reference values. Red boxes and text denote calculated quantities from the NAA resonance, and blue boxes and text denote calculated quantities from the water signal. Equations accompany the different calculation steps. $S_{NAA,step}$ denotes the NAA signal intensity in the corresponding protocol step, with the relaxation corrected intensities for NAA and parenchyma water given by $S_{NAA}$ and $S_p$ respectively.

**STEAM PROTOCOL SELECTION:**

*Two-Point Saturation Recovery STEAM*: To accurately measure changes in the parenchyma water concentration, $T2$ relaxometry of the water signal was performed using saturation recovery experiment. *In-vivo* experiments, were optimised with two-point saturation recovery by choosing an appropriate pair of TRs for accurate and precise measurement of metabolite concentrations. A pair of TRs sufficiently long to ensure near fully recovered Lip/MM signals (i.e. TR≥1500ms) were used.

*Choice of acquisition scheme for tNAA concentration*: This protocol comprises 10 separate STEAM acquisitions in the same $15\times15\times15mm^3$ thalamic voxel. In this study scan duration (approximately 13 minutes) for NAA concentration estimation is significantly shorter than in the PRESS protocol (~25 min) for the previous MARBLE study.

In the present two-point saturation recovery STEAM protocol (Table S2), steps 1 and 3 comprise a two-point $T1$ relaxometry experiment with TR 1500ms and 5000ms (or 3500ms) for each metabolite, allowing the calculation of fully $T1$ relaxed signal intensities. The short TE of 20ms ensures that differential $T2$ decay effects between metabolites and water are minimised. Steps 2 and 4 allow eddy current correction of steps 1 and 3, and can be combined to determine the fully $T1$ relaxed brain water signal under the same phase cycling scheme as steps 1 and 3.

Although STEAM acquisitions provide a lower inherent signal-to-noise ratio than PRESS, they are less susceptible to chemical shift displacement artefacts, and can achieve a shorter TE to minimise variation due to the variance $T2$ relaxation effects. Though, the use of a short TE complicates the fitting of key metabolites due to overlapping with broader resonances from lipids and macromolecules, their short $T1$ ensures that they are fully relaxed throughout the present experiment. The estimates of the NAA concentration and $T1$ of acetate were in agreement between PRESS (used in our earlier MARBLE study) and present STEAM protocols. Within normal variation and mild pathology, the PRESS and STEAM protocols are comparable in their measurements of [tNAA] in the neonatal brain, although STEAM tends to overestimate this.

*Optimising Two-Point Saturation Recovery:* Initial few STEAM acquisitions (total 07 included in analysis) were taken with TR =1500 ms and 3500ms. The protocol was further optimised by increasing TR from 3500ms to 5000ms. By increasing the TR of the second acquisition in the two-point saturation recovery experiment, the estimates of fully relaxed signals and $T1$s are less biased and more precise, which is a desirable property for robust estimation of metabolite concentrations. With the longer TR at 3500ms, estimates from the two acquisitions were sensitive to the signal to noise ratio, however, with 5000ms, fully $T1$ relaxed signal intensities are largely independent of the error at the shorter TR. In the proposed STEAM protocol, the Lip/MM resonances are fully relaxed due to $T1$ effects throughout the experiment. A TR of 5000ms was also observed as a reasonable balance of signal-to-noise ratio efficiency and overall fitting accuracy, while also allowing effective post-hoc motion correction on the collected sub-spectra.

**Figure 7. DTI analysis**

**DTI data acquisition protocol:**
DTI acquisition in 30 diffusion gradient directions were carried out using 2D spin-echo echo-planar imaging with 31 total images per slice (30 $b$ = 750 s/mm$^2$ and 1 $b$ = 0 s/mm$^2$), TE = 79 ms, 1·95×1·95×2 mm$^3$ voxel size and parallel imaging acceleration factor of 2·0.

**Quality assurance and processing:**
Data was assessed for excessive motion and acquisition artefacts, both before and after isolating the brain signal and undertaking corrections for eddy currents and motion using FSL.

Diffusion tensors were then calculated in FSL, and datasets of sufficient quality were spatially normalised to skull stripped JHU_neonate_SS_fass FA map. Major white matter tracts were selected by thresholding according to fractional anisotropy (FA > 0·15) and skeletonised using FSL.

To select a region of interest in the posterior limbs of the internal capsule, a binarized mask was extracted using JHU neonatal brain atlas and placed over mean FA map, as shown below:

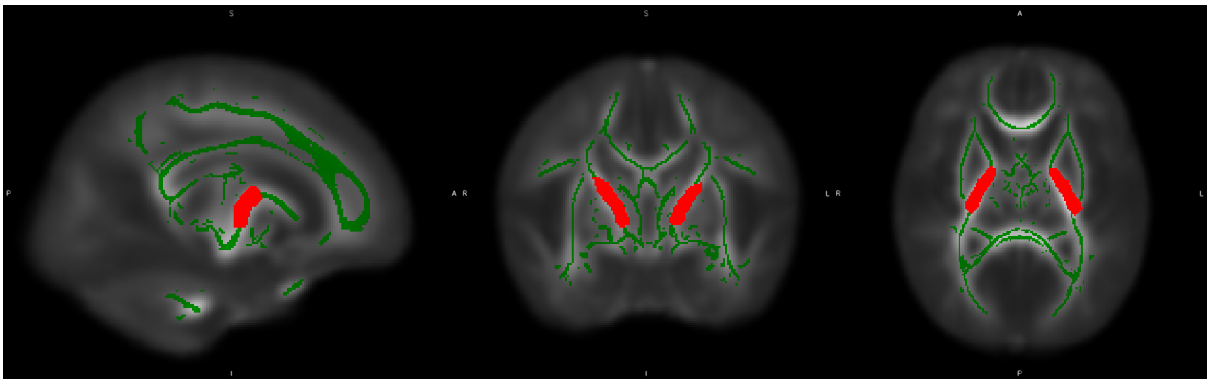

***Mean FA skeleton thresholded to include only major white matter tracts (green). Binarized mask at PLICs are shown overlaid (red)***

The mean FA and standard deviation within this region of interest was then used for assessing prognostic accuracy.

Tract-based spatial statistics (FSL) was then used to examine relationships between FA values in Hypothermia vs control study groups, estimated across all of the major white matter tracts included in the mean skeleton. These analyses were corrected for multiple comparisons using threshold-free cluster enhancement.

**Figure 8. Effect of hypothermia on mortality at hospital discharge: Subgroup analysis based on place of delivery.**

| Study or Subgroup | Cooling Events | Cooling Total | Usual Care Events | Usual Care Total | Weight | Risk Ratio M-H, Random, 95% CI |
|---|---|---|---|---|---|---|
| Inborn | 24 | 62 | 19 | 61 | 39.4% | 1.24 [0.76, 2.02] |
| Outborn | 48 | 140 | 30 | 145 | 60.6% | 1.66 [1.12, 2.45] |
| **Total (95% CI)** | | **202** | | **206** | **100.0%** | **1.48 [1.09, 2.01]** |
| Total events | 72 | | 49 | | | |

Heterogeneity: Tau² = 0.00; Chi² = 0.82, df = 1 (P = 0.37); I² = 0%
Test for overall effect: Z = 2.51 (P = 0.01)

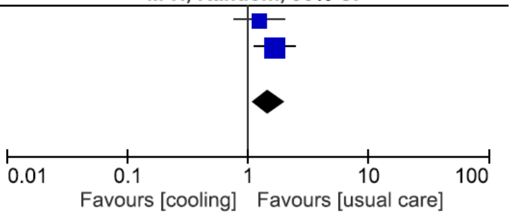


**Figure 9. Effect of hypothermia on mortality at hospital discharge: Subgroup analysis based on birth weight.**

| Study or Subgroup | Cooling Events | Cooling Total | Usual Care Events | Usual Care Total | Weight | Risk Ratio M-H, Random, 95% CI |
|---|---|---|---|---|---|---|
| Birth weight between 1.8-2.49 Kg | 20 | 37 | 8 | 29 | 23.8% | 1.96 [1.01, 3.79] |
| Birth weight between 2.5-3 Kg | 30 | 101 | 25 | 95 | 44.2% | 1.13 [0.72, 1.77] |
| Birth weight more than 3 kg | 22 | 64 | 16 | 82 | 31.9% | 1.76 [1.01, 3.07] |
| **Total (95% CI)** | | **202** | | **206** | **100.0%** | **1.48 [1.05, 2.10]** |
| Total events | 72 | | 49 | | | |

Heterogeneity: Tau² = 0.02; Chi² = 2.45, df = 2 (P = 0.29); I² = 19%
Test for overall effect: Z = 2.23 (P = 0.03)

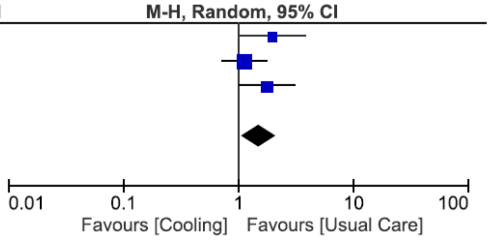


**Figure 10. Effect of hypothermia on mortality at hospital discharge: Subgroup analysis based on growth restriction**

| Study or Subgroup | Cooling Events | Cooling Total | Usual Care Events | Usual Care Total | Weight | Risk Ratio M-H, Fixed, 95% CI |
|---|---|---|---|---|---|---|
| Adequate for gestational age | 48 | 157 | 38 | 171 | 74.6% | 1.38 [0.95, 1.98] |
| Small for gestational age | 24 | 45 | 11 | 35 | 25.4% | 1.70 [0.97, 2.97] |
| **Total (95% CI)** | | **202** | | **206** | **100.0%** | **1.46 [1.07, 1.98]** |
| Total events | 72 | | 49 | | | |

Heterogeneity: Chi² = 0.38, df = 1 (P = 0.54); I² = 0%
Test for overall effect: Z = 2.41 (P = 0.02)

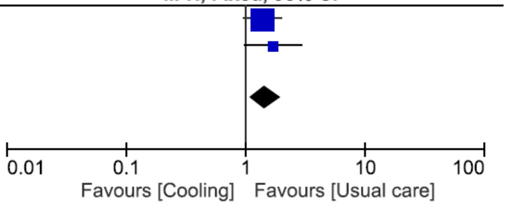


Small for gestational age was defined as birth weight less than 2 standard deviations on the World Health Organisation growth chart

**Figure 11. Effect of hypothermia on mortality at hospital discharge: Subgroup analysis based on co-existent sepsis.**

| Study or Subgroup | Cooling Events | Cooling Total | Usual Care Events | Usual Care Total | Weight | Risk Ratio M-H, Fixed, 95% CI |
|---|---|---|---|---|---|---|
| Adequate for gestational age | 48 | 157 | 38 | 171 | 74.6% | 1.38 [0.95, 1.98] |
| Small for gestational age | 24 | 45 | 11 | 35 | 25.4% | 1.70 [0.97, 2.97] |
| **Total (95% CI)** | | **202** | | **206** | **100.0%** | **1.46 [1.07, 1.98]** |
| Total events | 72 | | 49 | | | |

Heterogeneity: Chi² = 0.38, df = 1 (P = 0.54); I² = 0%
Test for overall effect: Z = 2.41 (P = 0.02)

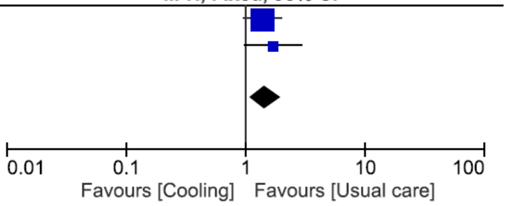


**Figure 12. Effect of hypothermia on mortality at hospital discharge: Subgroup analysis based on perinatal sentinel events**

| Study or Subgroup | Cooling Events | Cooling Total | Usual Care Events | Usual Care Total | Weight | Risk Ratio M-H, Random, 95% CI |
|---|---|---|---|---|---|---|
| No perinatal sentinel events | 65 | 185 | 43 | 180 | 88.5% | 1.47 [1.06, 2.04] |
| Presence of perinatal sentinel events | 7 | 17 | 6 | 26 | 11.5% | 1.78 [0.72, 4.40] |
| **Total (95% CI)** | | **202** | | **206** | **100.0%** | **1.50 [1.11, 2.04]** |
| Total events | 72 | | 49 | | | |

Heterogeneity: Tau² = 0.00; Chi² = 0.16, df = 1 (P = 0.69); I² = 0%
Test for overall effect: Z = 2.61 (P = 0.009)

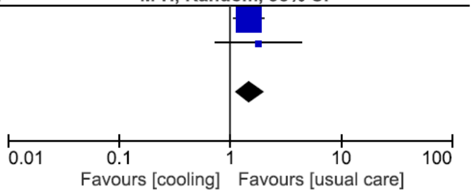

**Figure 13. Effect of hypothermia on mortality at hospital discharge: Subgroup analysis based on socio-demographic index of the region.**

| Study or Subgroup | Cooling Events | Cooling Total | Usual Care Events | Usual Care Total | Weight | Risk Ratio M-H, Random, 95% CI |
|---|---|---|---|---|---|---|
| **2.7.1 High-middle SDI** | | | | | | |
| ICH Chennai | 27 | 58 | 17 | 61 | 37.8% | 1.67 [1.02, 2.72] |
| IOG Chennai | 11 | 26 | 11 | 27 | 22.1% | 1.04 [0.55, 1.97] |
| KLN Sri Lanka | 6 | 15 | 3 | 13 | 6.6% | 1.73 [0.54, 5.59] |
| **Subtotal (95% CI)** | | **99** | | **101** | **66.6%** | **1.43 [0.99, 2.07]** |
| Total events | 44 | | 31 | | | |
| Heterogeneity: Tau² = 0.00; Chi² = 1.46, df = 2 (P = 0.48); I² = 0% | | | | | | |
| Test for overall effect: Z = 1.91 (P = 0.06) | | | | | | |
| **2.7.2 Middle SDI** | | | | | | |
| ICH Bangalore | 9 | 54 | 7 | 52 | 10.9% | 1.24 [0.50, 3.08] |
| LTMGH Mumbai | 11 | 28 | 6 | 29 | 12.6% | 1.90 [0.81, 4.44] |
| SAT, Trivandrum | 2 | 5 | 1 | 7 | 2.0% | 2.80 [0.34, 23.06] |
| **Subtotal (95% CI)** | | **87** | | **88** | **25.5%** | **1.63 [0.90, 2.96]** |
| Total events | 22 | | 14 | | | |
| Heterogeneity: Tau² = 0.00; Chi² = 0.73, df = 2 (P = 0.69); I² = 0% | | | | | | |
| Test for overall effect: Z = 1.61 (P = 0.11) | | | | | | |
| **2.7.3 Low-middle SDI** | | | | | | |
| BSMMU Dhaka | 6 | 16 | 4 | 17 | 8.0% | 1.59 [0.55, 4.62] |
| **Subtotal (95% CI)** | | **16** | | **17** | **8.0%** | **1.59 [0.55, 4.62]** |
| Total events | 6 | | 4 | | | |
| Heterogeneity: Not applicable | | | | | | |
| Test for overall effect: Z = 0.86 (P = 0.39) | | | | | | |
| **Total (95% CI)** | | **202** | | **206** | **100.0%** | **1.49 [1.11, 2.02]** |
| Total events | 72 | | 49 | | | |
| Heterogeneity: Tau² = 0.00; Chi² = 2.34, df = 6 (P = 0.89); I² = 0% | | | | | | |
| Test for overall effect: Z = 2.61 (P = 0.009) | | | | | | |
| Test for subgroup differences: Chi² = 0.15, df = 2 (P = 0.93), I² = 0% | | | | | | |

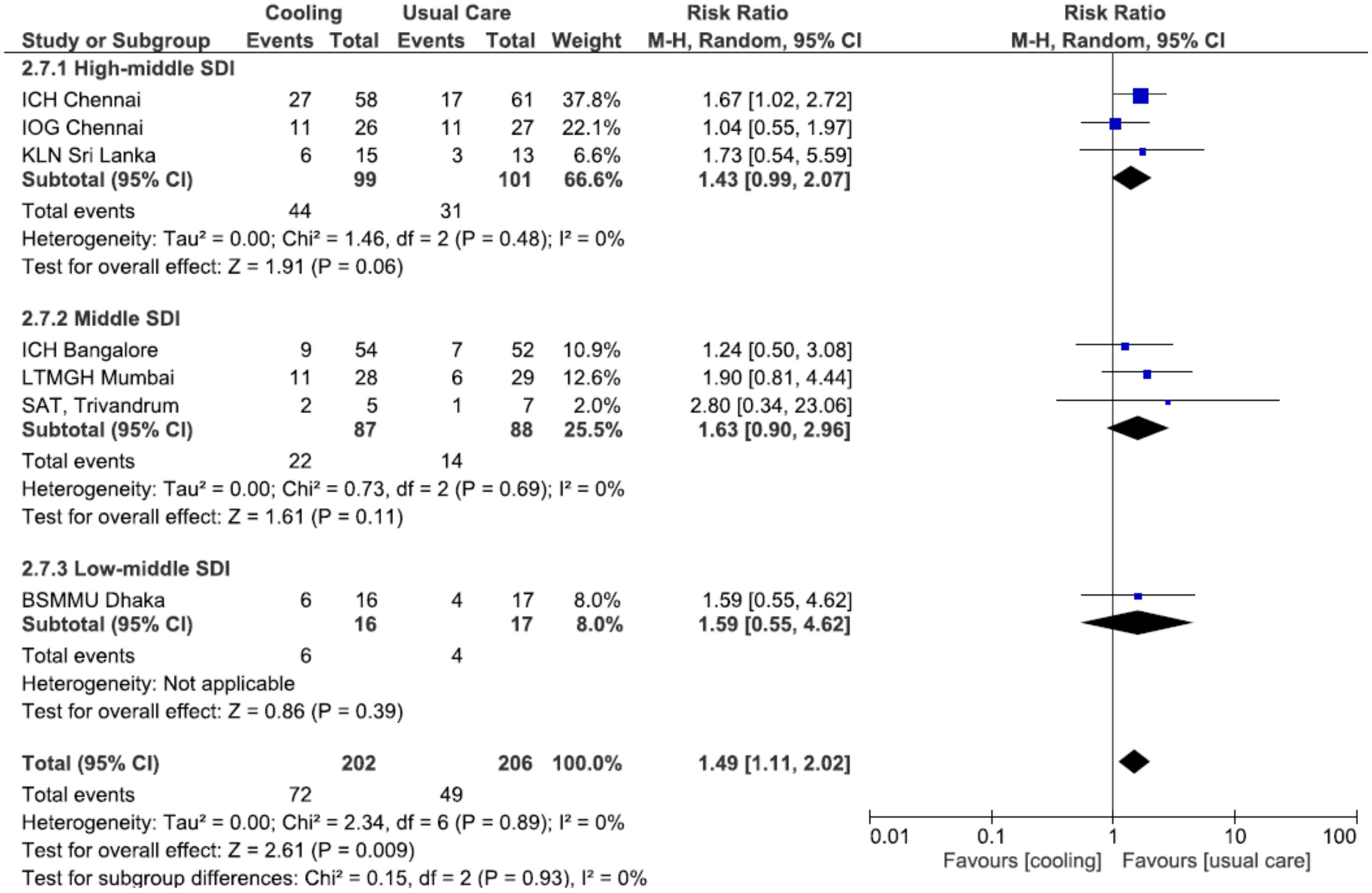


**Figure 14. Effect of hypothermia on mortality at hospital discharge: Subgroup analysis based on neonatal mortality rate of the region**

| Study or Subgroup | Cooling Events | Cooling Total | Usual Care Events | Usual Care Total | Weight | Risk Ratio M-H, Random, 95% CI |
|---|---|---|---|---|---|---|
| **2.6.1 High** | | | | | | |
| BSMMU Dhaka | 6 | 16 | 4 | 17 | 8.0% | 1.59 [0.55, 4.62] |
| ICH Bangalore | 9 | 54 | 7 | 52 | 10.9% | 1.24 [0.50, 3.08] |
| ICH Chennai | 27 | 58 | 17 | 61 | 37.8% | 1.67 [1.02, 2.72] |
| IOG Chennai | 11 | 26 | 11 | 27 | 22.1% | 1.04 [0.55, 1.97] |
| LTMGH Mumbai | 11 | 28 | 6 | 29 | 12.6% | 1.90 [0.81, 4.44] |
| **Subtotal (95% CI)** | | **182** | | **186** | **91.4%** | **1.46 [1.06, 1.99]** |
| Total events | 64 | | 45 | | | |
| Heterogeneity: Tau² = 0.00; Chi² = 1.91, df = 4 (P = 0.75); I² = 0% | | | | | | |
| Test for overall effect: Z = 2.34 (P = 0.02) | | | | | | |
| **2.6.3 Low** | | | | | | |
| KLN Sri Lanka | 6 | 15 | 3 | 13 | 6.6% | 1.73 [0.54, 5.59] |
| SAT, Trivandrum | 2 | 5 | 1 | 7 | 2.0% | 2.80 [0.34, 23.06] |
| **Subtotal (95% CI)** | | **20** | | **20** | **8.6%** | **1.94 [0.70, 5.40]** |
| Total events | 8 | | 4 | | | |
| Heterogeneity: Tau² = 0.00; Chi² = 0.15, df = 1 (P = 0.70); I² = 0% | | | | | | |
| Test for overall effect: Z = 1.27 (P = 0.20) | | | | | | |
| **Total (95% CI)** | | **202** | | **206** | **100.0%** | **1.49 [1.11, 2.02]** |
| Total events | 72 | | 49 | | | |
| Heterogeneity: Tau² = 0.00; Chi² = 2.34, df = 6 (P = 0.89); I² = 0% | | | | | | |
| Test for overall effect: Z = 2.61 (P = 0.009) | | | | | | |
| Test for subgroup differences: Chi² = 0.28, df = 1 (P = 0.60), I² = 0% | | | | | | |

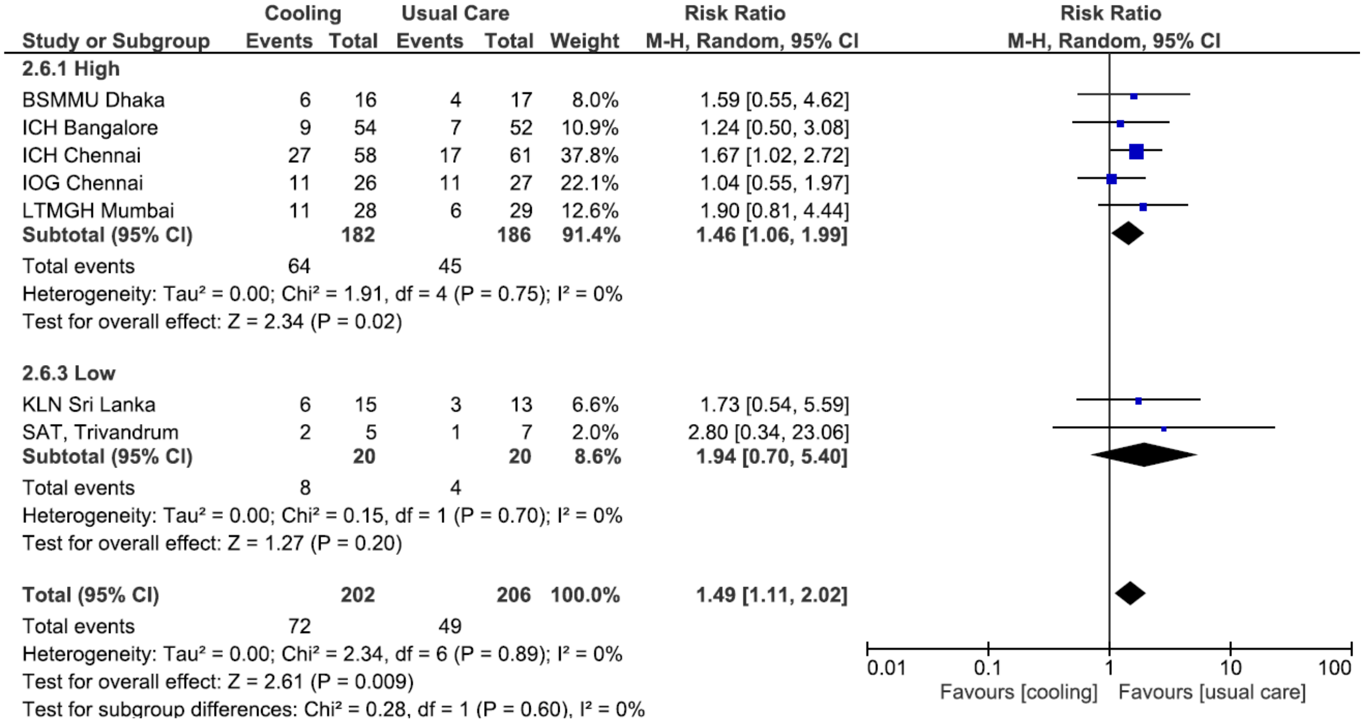

**Figure 15. Effect of hypothermia on death or disability at 18 months: Subgroup analysis based on place of delivery.**

| Study or Subgroup | Cooling Events | Cooling Total | Usual Care Events | Usual Care Total | Weight | Risk Ratio M-H, Random, 95% CI |
|---|---|---|---|---|---|---|
| Inborn | 34 | 58 | 34 | 60 | 41.7% | 1.03 [0.76, 1.41] |
| Outborn | 64 | 137 | 60 | 139 | 58.3% | 1.08 [0.83, 1.41] |
| **Total (95% CI)** | | **195** | | **199** | **100.0%** | **1.06 [0.87, 1.30]** |
| Total events | 98 | | 94 | | | |

Heterogeneity: Tau² = 0.00; Chi² = 0.05, df = 1 (P = 0.83); I² = 0%
Test for overall effect: Z = 0.59 (P = 0.55)

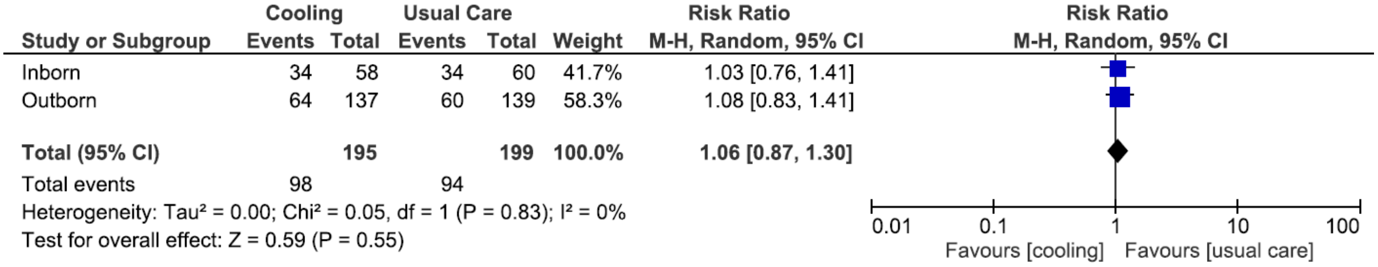


**Figure 16. Effect of hypothermia on death or disability at 18 months: Subgroup analysis based on birth weight.**

| Study or Subgroup | Cooling Events | Cooling Total | Usual Care Events | Usual Care Total | Weight | Risk Ratio M-H, Random, 95% CI |
|---|---|---|---|---|---|---|
| Birth weight between 1.8-2.49 Kg | 26 | 37 | 19 | 29 | 33.6% | 1.07 [0.77, 1.50] |
| Birth weight between 2.5-3 Kg | 44 | 96 | 43 | 92 | 40.2% | 0.98 [0.72, 1.33] |
| Birth weight more than 3 kg | 28 | 62 | 32 | 78 | 26.1% | 1.10 [0.75, 1.61] |
| **Total (95% CI)** | | **195** | | **199** | **100.0%** | **1.04 [0.86, 1.27]** |
| Total events | 98 | | 94 | | | |

Heterogeneity: Tau² = 0.00; Chi² = 0.26, df = 2 (P = 0.88); I² = 0%
Test for overall effect: Z = 0.41 (P = 0.68)

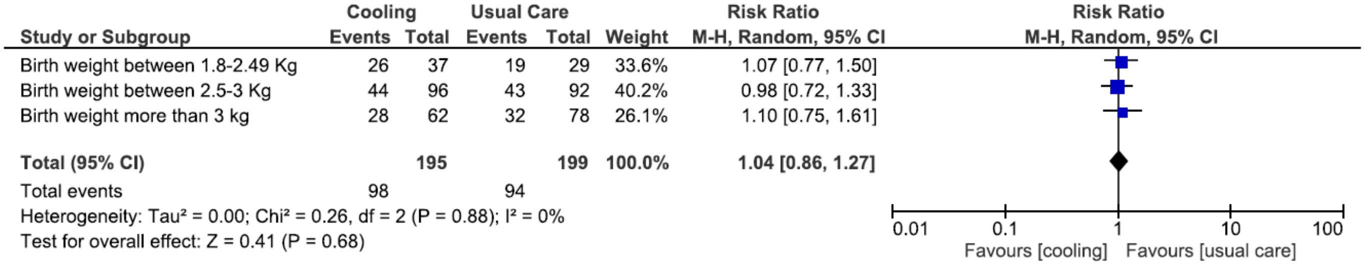


**Figure 17. Effect of hypothermia on death or disability at 18 months: Subgroup analysis based on growth restriction**

| Study or Subgroup | Cooling Events | Cooling Total | Usual Care Events | Usual Care Total | Weight | Risk Ratio M-H, Fixed, 95% CI |
|---|---|---|---|---|---|---|
| Adequate for gestational age | 68 | 151 | 70 | 164 | 71.5% | 1.06 [0.82, 1.35] |
| Small for gestational age | 30 | 44 | 24 | 35 | 28.5% | 0.99 [0.74, 1.34] |
| **Total (95% CI)** | | **195** | | **199** | **100.0%** | **1.04 [0.85, 1.27]** |
| Total events | 98 | | 94 | | | |

Heterogeneity: Chi² = 0.09, df = 1 (P = 0.76); I² = 0%
Test for overall effect: Z = 0.37 (P = 0.72)

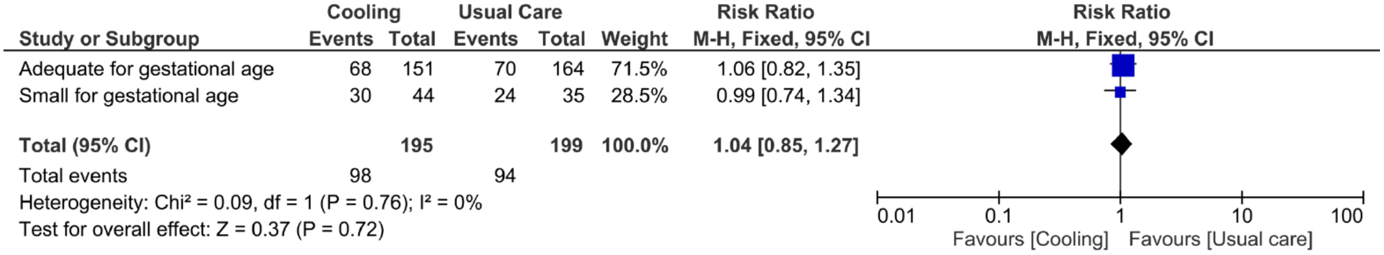


Small for gestational age was defined as birth weight less than 2 standard deviations on the World Health Organisation growth chart

**Figure 18. Effect of hypothermia on death or disability at 18 months: Subgroup analysis based on co-existent sepsis.**

| Study or Subgroup | Cooling Events | Cooling Total | Usual Care Events | Usual Care Total | Weight | Risk Ratio M-H, Random, 95% CI |
|---|---|---|---|---|---|---|
| Early or late onset sepsis present | 39 | 69 | 40 | 61 | 50.2% | 0.86 [0.65, 1.14] |
| No sepsis | 59 | 126 | 54 | 138 | 49.8% | 1.20 [0.91, 1.58] |
| **Total (95% CI)** | | **195** | | **199** | **100.0%** | **1.01 [0.73, 1.41]** |
| Total events | 98 | | 94 | | | |

Heterogeneity: Tau² = 0.04; Chi² = 2.79, df = 1 (P = 0.10); I² = 64%
Test for overall effect: Z = 0.09 (P = 0.93)

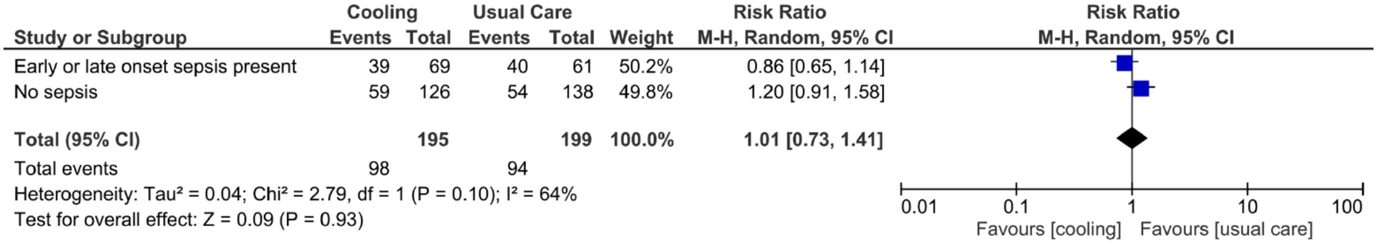


**Figure 19. Effect of hypothermia on death or disability at 18 months: Subgroup analysis based on perinatal sentinel events**

| Study or Subgroup | Cooling Events | Cooling Total | Usual Care Events | Usual Care Total | Weight | Risk Ratio M-H, Random, 95% CI |
|---|---|---|---|---|---|---|
| No perinatal sentinel events | 90 | 178 | 78 | 174 | 70.8% | 1.13 [0.91, 1.41] |
| Presence of perinatal sentinel events | 8 | 17 | 16 | 25 | 29.2% | 0.74 [0.41, 1.32] |
| **Total (95% CI)** | | **195** | | **199** | **100.0%** | **1.00 [0.68, 1.46]** |
| Total events | 98 | | 94 | | | |

Heterogeneity: Tau² = 0.04; Chi² = 1.81, df = 1 (P = 0.18); I² = 45%
Test for overall effect: Z = 0.02 (P = 0.98)

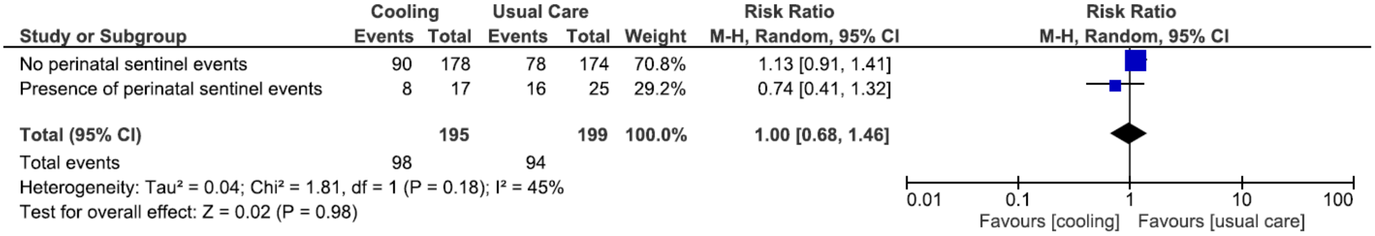

**Figure 20. Effect of hypothermia on death or disability at 18 months: Subgroup analysis based on socio-demographic index (SDI) of the region.**

| Study or Subgroup | Cooling Events | Cooling Total | Usual Care Events | Usual Care Total | Weight | Risk Ratio M-H, Random, 95% CI |
|---|---|---|---|---|---|---|
| **2.12.1 High-middle SDI** | | | | | | |
| ICH Chennai | 27 | 55 | 23 | 57 | 22.6% | 1.22 [0.80, 1.84] |
| IOG Chennai | 16 | 26 | 14 | 27 | 17.3% | 1.19 [0.74, 1.91] |
| KLN Sri Lanka | 7 | 15 | 7 | 13 | 7.1% | 0.87 [0.41, 1.81] |
| **Subtotal (95% CI)** | | **96** | | **97** | **47.1%** | **1.15 [0.86, 1.53]** |
| Total events | 50 | | 44 | | | |
| Heterogeneity: Tau² = 0.00; Chi² = 0.65, df = 2 (P = 0.72); I² = 0% | | | | | | |
| Test for overall effect: Z = 0.92 (P = 0.36) | | | | | | |
| **2.12.2 Middle SDI** | | | | | | |
| ICH Bangalore | 21 | 54 | 20 | 51 | 17.0% | 0.99 [0.61, 1.60] |
| LTMGH Mumbai | 16 | 24 | 16 | 27 | 21.9% | 1.13 [0.74, 1.72] |
| SAT, Trivandrum | 3 | 5 | 4 | 7 | 4.2% | 1.05 [0.40, 2.75] |
| **Subtotal (95% CI)** | | **83** | | **85** | **43.1%** | **1.06 [0.79, 1.44]** |
| Total events | 40 | | 40 | | | |
| Heterogeneity: Tau² = 0.00; Chi² = 0.16, df = 2 (P = 0.92); I² = 0% | | | | | | |
| Test for overall effect: Z = 0.40 (P = 0.69) | | | | | | |
| **2.12.3 Low-middle SDI** | | | | | | |
| BSMMU Dhaka | 8 | 16 | 10 | 17 | 9.8% | 0.85 [0.45, 1.60] |
| **Subtotal (95% CI)** | | **16** | | **17** | **9.8%** | **0.85 [0.45, 1.60]** |
| Total events | 8 | | 10 | | | |
| Heterogeneity: Not applicable | | | | | | |
| Test for overall effect: Z = 0.50 (P = 0.61) | | | | | | |
| **Total (95% CI)** | | **195** | | **199** | **100.0%** | **1.08 [0.88, 1.31]** |
| Total events | 98 | | 94 | | | |
| Heterogeneity: Tau² = 0.00; Chi² = 1.52, df = 6 (P = 0.96); I² = 0% | | | | | | |
| Test for overall effect: Z = 0.74 (P = 0.46) | | | | | | |
| Test for subgroup differences: Chi² = 0.72, df = 2 (P = 0.70), I² = 0% | | | | | | |

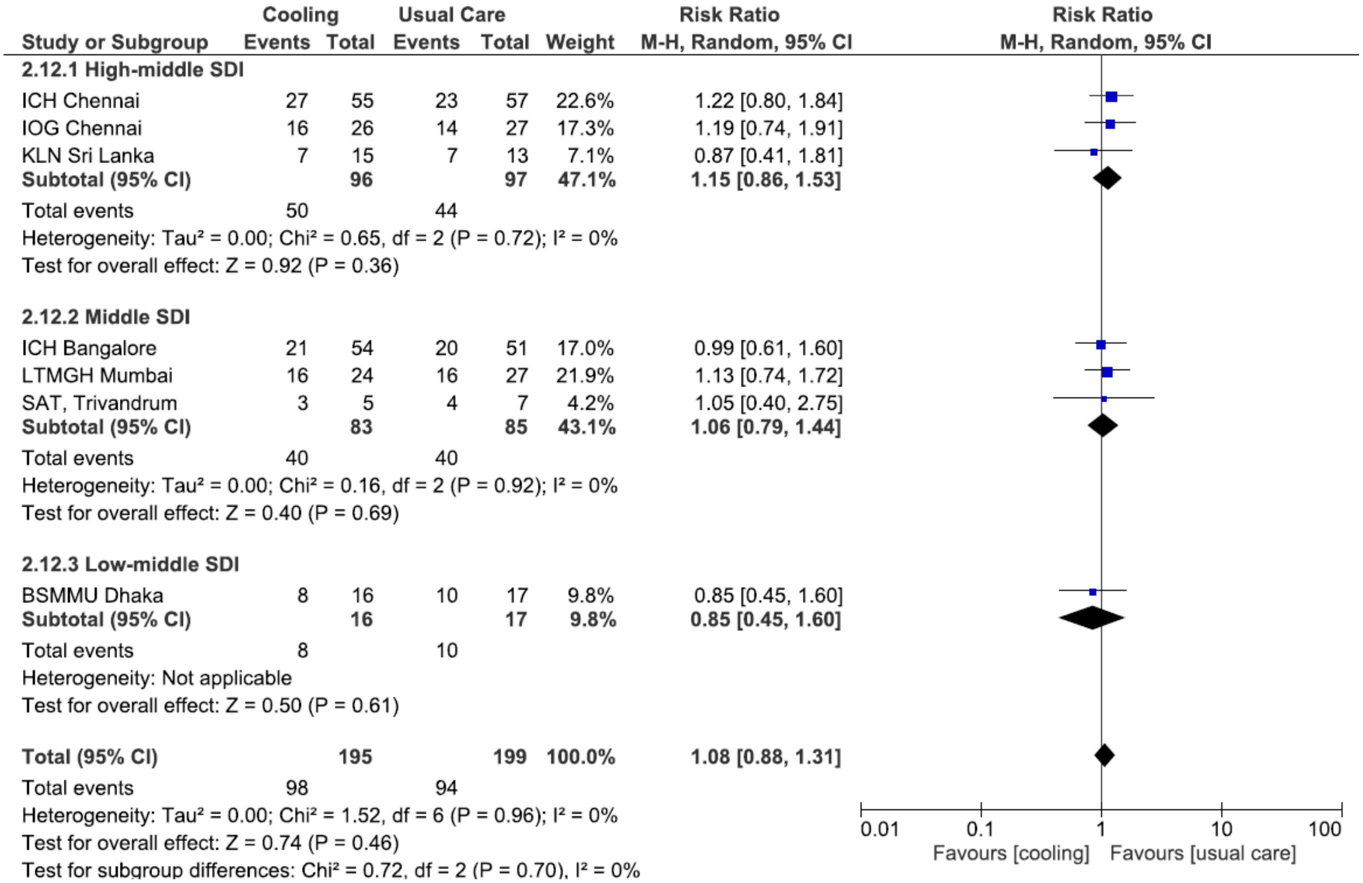


**Figure 21. Effect of hypothermia on death or disability at 18 months: Subgroup analysis based on neonatal mortality rate of the region**

| Study or Subgroup | Cooling Events | Cooling Total | Usual Care Events | Usual Care Total | Weight | Risk Ratio M-H, Random, 95% CI |
|---|---|---|---|---|---|---|
| **2.14.1 High** | | | | | | |
| BSMMU Dhaka | 8 | 16 | 10 | 17 | 9.8% | 0.85 [0.45, 1.60] |
| ICH Bangalore | 21 | 54 | 20 | 51 | 17.0% | 0.99 [0.61, 1.60] |
| ICH Chennai | 27 | 55 | 23 | 57 | 22.6% | 1.22 [0.80, 1.84] |
| IOG Chennai | 16 | 26 | 14 | 27 | 17.3% | 1.19 [0.74, 1.91] |
| LTMGH Mumbai | 16 | 24 | 16 | 27 | 21.9% | 1.13 [0.74, 1.72] |
| **Subtotal (95% CI)** | | **175** | | **179** | **88.7%** | **1.10 [0.89, 1.35]** |
| Total events | 88 | | 83 | | | |
| Heterogeneity: Tau² = 0.00; Chi² = 1.16, df = 4 (P = 0.88); I² = 0% | | | | | | |
| Test for overall effect: Z = 0.87 (P = 0.38) | | | | | | |
| **2.14.2 Low** | | | | | | |
| KLN Sri Lanka | 7 | 15 | 7 | 13 | 7.1% | 0.87 [0.41, 1.81] |
| SAT, Trivandrum | 3 | 5 | 4 | 7 | 4.2% | 1.05 [0.40, 2.75] |
| **Subtotal (95% CI)** | | **20** | | **20** | **11.3%** | **0.93 [0.52, 1.67]** |
| Total events | 10 | | 11 | | | |
| Heterogeneity: Tau² = 0.00; Chi² = 0.10, df = 1 (P = 0.76); I² = 0% | | | | | | |
| Test for overall effect: Z = 0.24 (P = 0.81) | | | | | | |
| **Total (95% CI)** | | **195** | | **199** | **100.0%** | **1.08 [0.88, 1.31]** |
| Total events | 98 | | 94 | | | |
| Heterogeneity: Tau² = 0.00; Chi² = 1.52, df = 6 (P = 0.96); I² = 0% | | | | | | |
| Test for overall effect: Z = 0.74 (P = 0.46) | | | | | | |
| Test for subgroup differences: Chi² = 0.27, df = 1 (P = 0.60), I² = 0% | | | | | | |

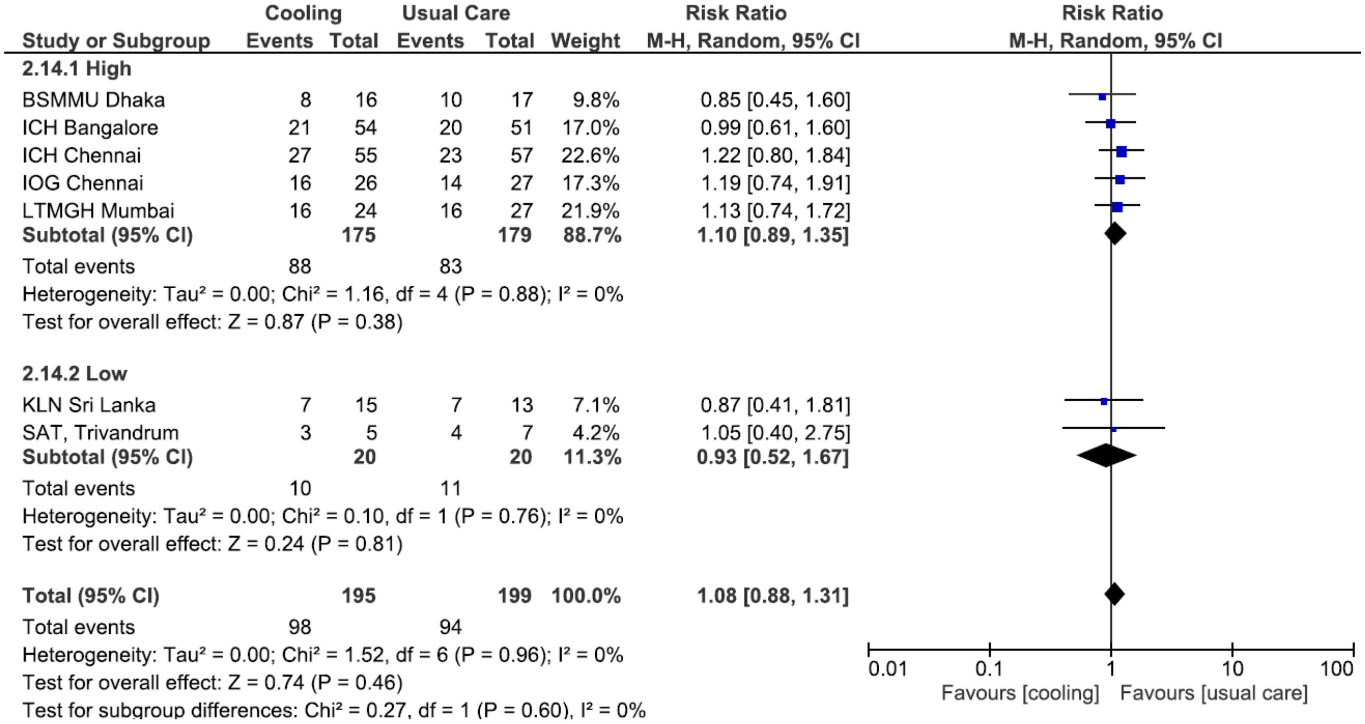


High neonatal mortality: 10 to 20 per 1000 live births; Low neonatal mortality <10 per 1000 live birth

**Figure 22. Relation of neonatal mortality (left forest plot) and moderate or severe disability (right forest plot) and therapeutic hypothermia.**

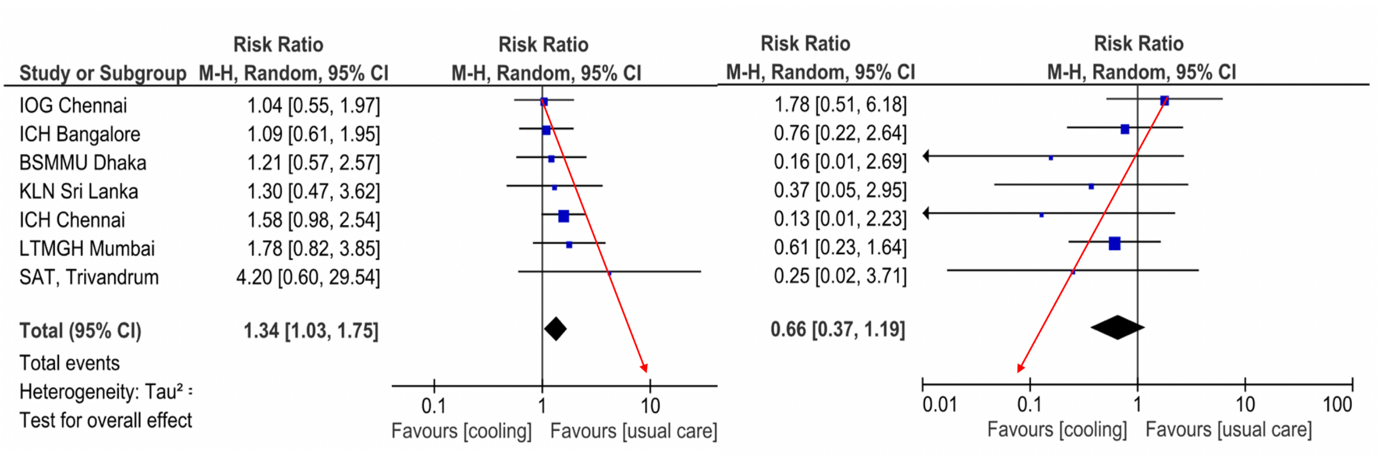


The centres are arranged in the order of increasing mortality in the left panel. The centers with higher mortality appears to have lower rates of disability.
